# Probing the Electronic Properties and Interaction Landscapes in a Series of *N*-(Chlorophenyl)pyridinecarboxamides

**DOI:** 10.1021/acs.cgd.2c00153

**Published:** 2022-04-13

**Authors:** John F. Gallagher, Niall Hehir, Pavle Mocilac, Chloé Violin, Brendan F. O’Connor, Emmanuel Aubert, Enrique Espinosa, Benoît Guillot, Christian Jelsch

**Affiliations:** † School of Chemical Sciences, Dublin City University, Dublin D09 DXA0, Ireland; ‡ School of Biotechnology, Dublin City University, Dublin D09 DXA0, Ireland; § CRM^2^, CNRS UMR 7036, Faculté des Sciences et Technologies, Université de Lorraine, BP 70239, Boulevard des Aiguillettes, 54506 Vandoeuvre-lès-Nancy, France

## Abstract

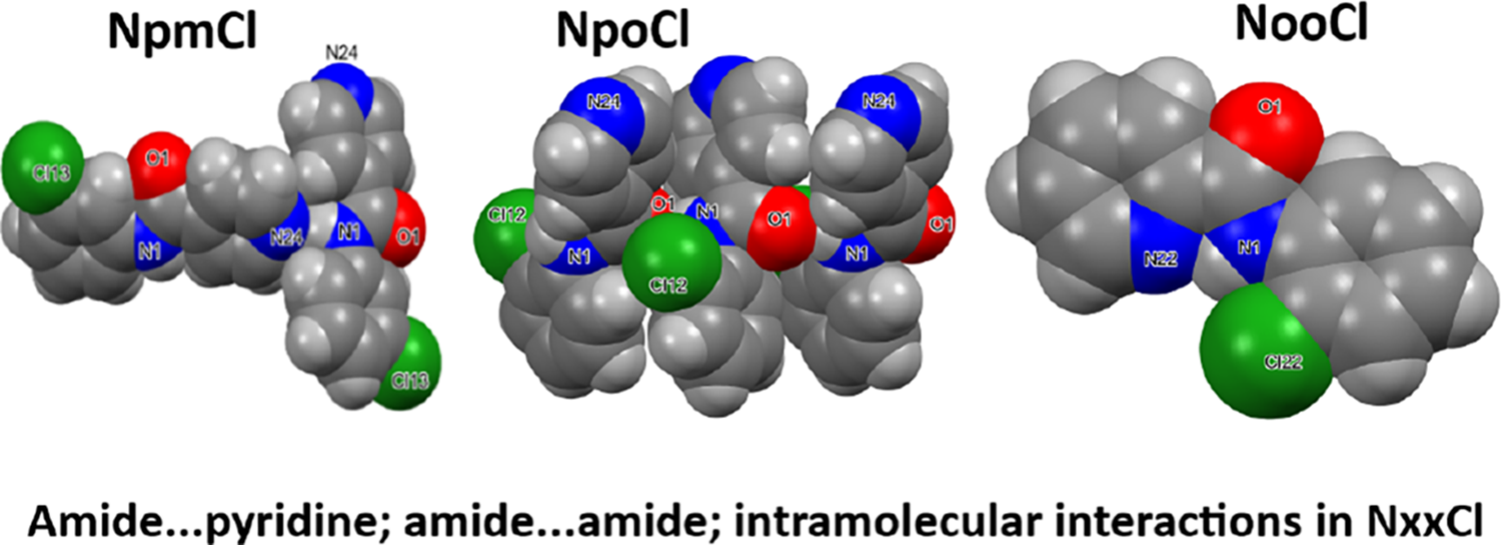

A 3 × 3 isomer grid of nine *N*-(chlorophenyl)pyridinecarboxamides (**NxxCl**) is reported with physicochemical studies and single crystal structures (**Nx** = pyridinoyl moiety; **xCl** = aminochlorobenzene ring; **x** = *para*-/*meta*-/*ortho*-), as synthesized by the reaction of the substituted *p*-/*m*-/*o*-pyridinecarbonyl chlorides (**Nx**) with *p*-/*m*-/*o*-aminochlorobenzenes (**xCl**). Several of the nine **NxxCl** crystal structures display structural similarities with their halogenated **NxxX** and methylated **NxxM** relatives (**x** = *p*-/*m-*/*o*-substitutions; **X** = F, Br; **M** = methyl). Indeed, five of the nine **NxxCl** crystal structures are isomorphous with their **NxxBr** analogues as the **NpmCl**/**Br**, **NpoCl**/**Br**, **NmoCl**/**NmoBr**, **NopCl**/**Br**, and **NooCl**/**Br** pairs. In the **NxxCl** series, the favored hydrogen bonding mode is aggregation by N–H···N_pyridine_ interactions, though amide···amide intermolecular interactions are noted in **NpoCl** and **NmoCl**. For the **NoxCl** triad, intramolecular N–H···N_pyridine_ interactions influence molecular planarity, whereas **NppCl·H_2_O** (as a monohydrate) exhibits O–H···O, N–H···O1W, and O1W-H···N interactions as the primary hydrogen bonding. Analysis of chlorine-containing compounds on the CSD is noted for comparisons. The interaction environments are probed using Hirshfeld surface analysis and contact enrichment studies. The melting temperatures (*T*_m_) depend on both the lattice energy and molecular symmetry (Carnelley’s rule), and the melting points can be well predicted from a linear regression of the two variables. The relationships of the *T*_m_ values with the total energy, the electrostatic component, and the strongest hydrogen bond components have been analyzed.

## Abstract 2

Physicochemical properties, conformational analyses, and crystal structures of a 3 × 3 isomer grid of *N*-(chlorophenyl)pyridinecarboxamides (**NxxCl**) (**x** = *para*-/*meta*-/*ortho*-) are reported. Interaction environments are probed using Hirshfeld surface analysis and contact enrichment studies. Melting temperatures (*T*_m_) depend on the lattice energy and molecular symmetry (Carnelley’s rule). Relationships of *T*_m_ with the total energy, electrostatic component, and strongest hydrogen bond components are analyzed.

## Introduction

Organohalogens (as a class of organic chemicals) have seen a dramatic increase in research activity over the past 3 decades in a range of scientific fields such as atmospheric chemistry, pharmaceuticals, and agrochemicals.^1−14^ These research studies include both basic and applied research together with industrial applications.^5,9^ Ongoing structural chemistry research on organohalogens includes investigations on halogen bonding and intermolecular interactions;^14^ these studies have led to considerable developments and insights into our understanding of bonding and aggregation modes.^14−32^ Extensive structural studies have been undertaken on series of organohalogens. Examples include the investigation of fluorine in benzamides^33−36^ and potential uses of bromine and iodine in agrochemicals.^37−39^

Organochlorines have attracted considerable interest in the pharmaceutical sector^40−46^ and especially in agrochemicals (herbicides and pesticides) with uses as antihelminthic drugs such as niclosamide (an orally bioavailable chlorinated salicylanilide).^41^ Some of these have raised public concern mainly due to their disposal, waste treatment, and environmental problems.^4,47,48^ In tandem with drug development, there has been a surge in the study and use of halogens in new drugs and especially fluorine and chlorine in pharmaceuticals.^33−36,40−46^

The role and importance of the Cambridge Structural Database (CSD) as a tool for understanding structural systematics have been noted.^49^ As such, the development of structural systematics in pharmaceutical sciences is critical as one seeks to establish correlations in physicochemical relationships between series of molecules.^14^ In analyzing the electronic properties of series of compounds (such as *n* × *m* carboxamide isomer grids), the ability to observe trends in fundamental properties is essential. As advances in this area continue to be made, it is essential that our ability to assess tens, hundreds, or thousands of related structures is made easier.^49^ A key is to reduce the number of parameters and elucidate genuine relationships and correlations to aid in the development of new pharmaceutical drugs.^7,8,41−43,49^

**Figure 1:**
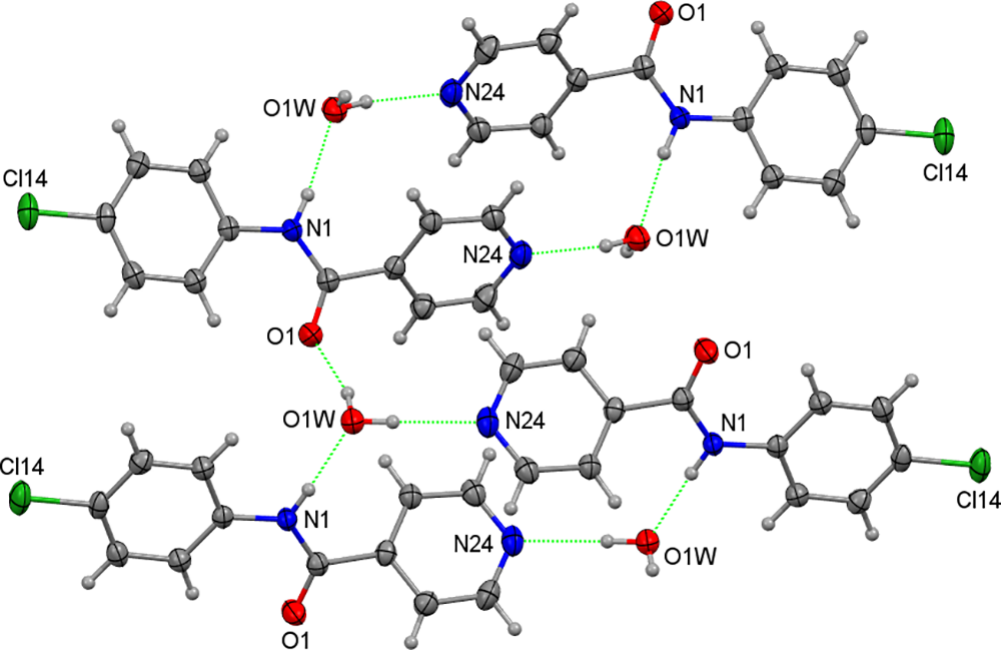
A view of [**NppCl·H_2_O**]_2_ linked by an O1W–H2W···O1 interaction.

**Figure 2:**
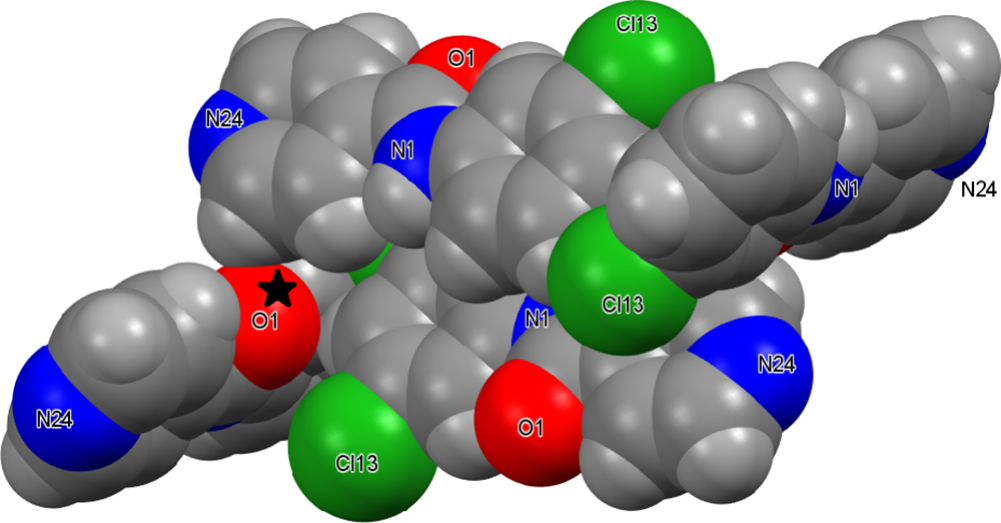
A view of the C–H···O1=C1 interactions in **NpmCl**.

**Figure 3:**
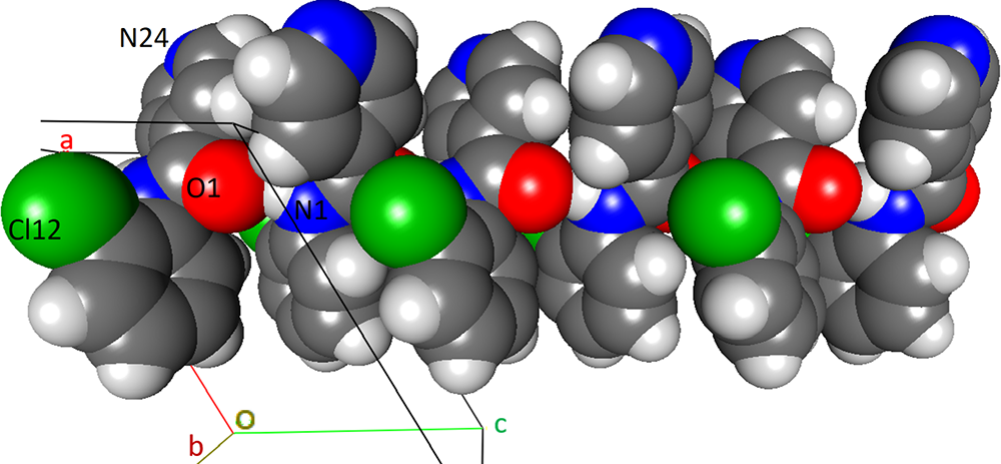
Crystallographic autostereogram of the 1D amide···amide chains along the *c* axis in **NpoCl** (atoms drawn as van der Waals spheres).

**Figure 4:**
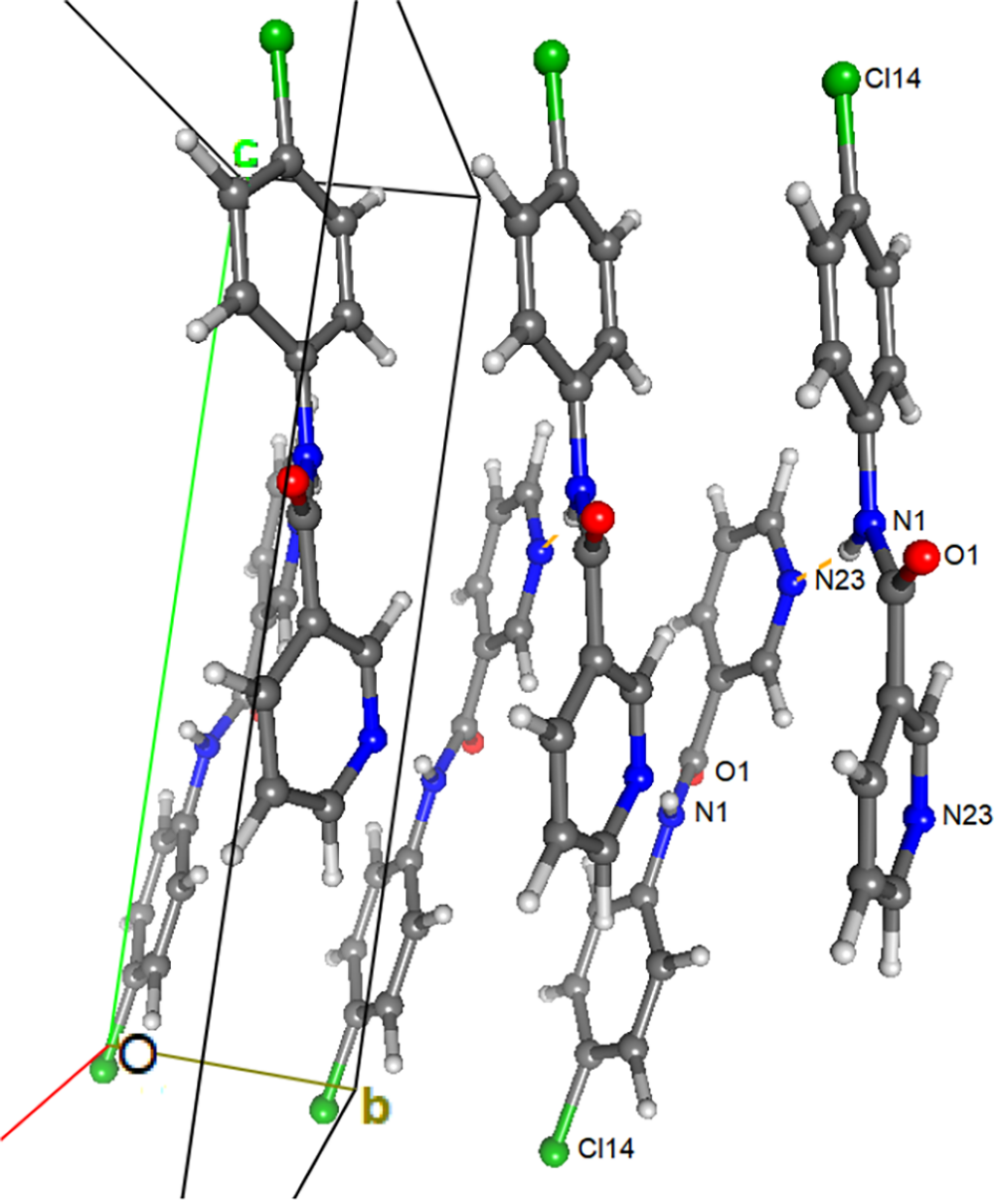
Crystallographic autostereogram of the _amide_N–H···N_pyridine_*zig-zag* chains in **NmpCl**.

**Figure 5:**
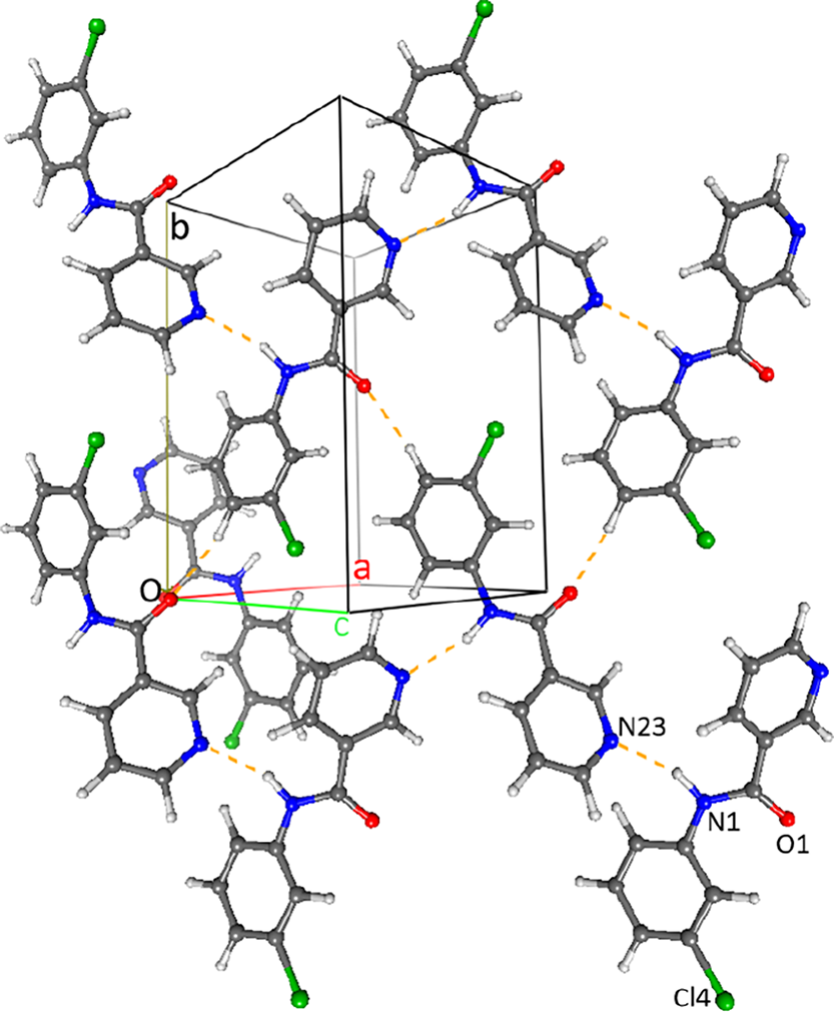
Crystallographic autostereogram showing the 1D *zig-zag* N–H···N chains as linked by C–H···O interactions in **NmmCl**.

**Figure 6:**
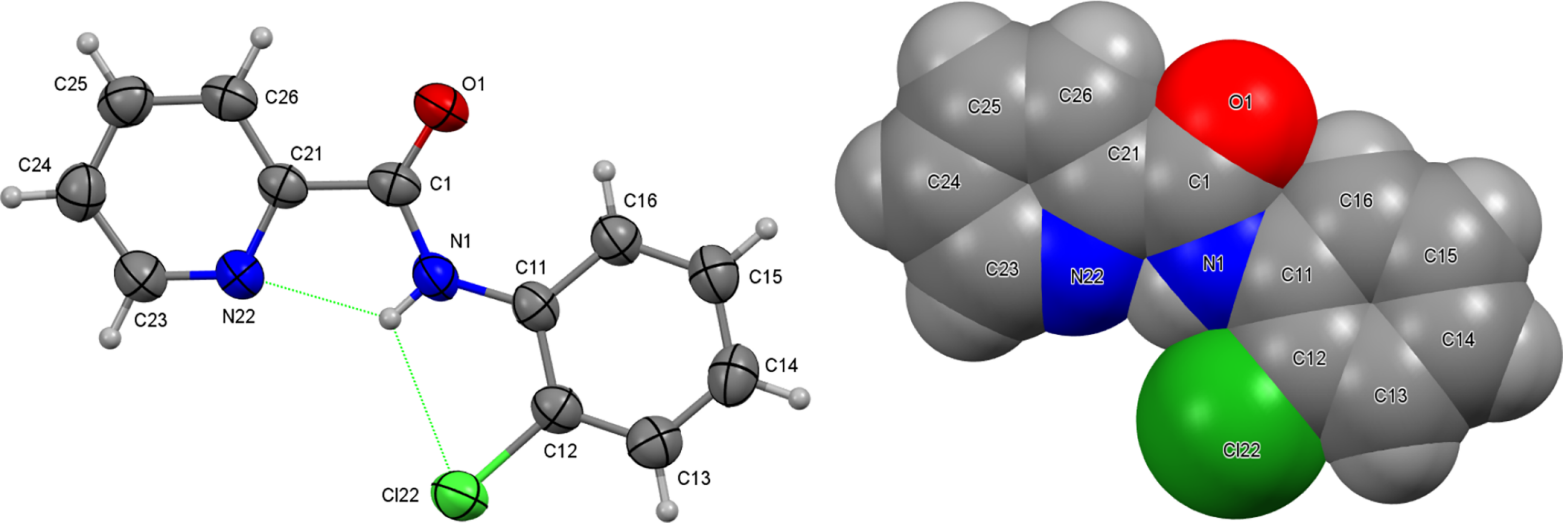
Molecular structure and intramolecular hydrogen bonding in **NooCl**.

**Figure 7:**
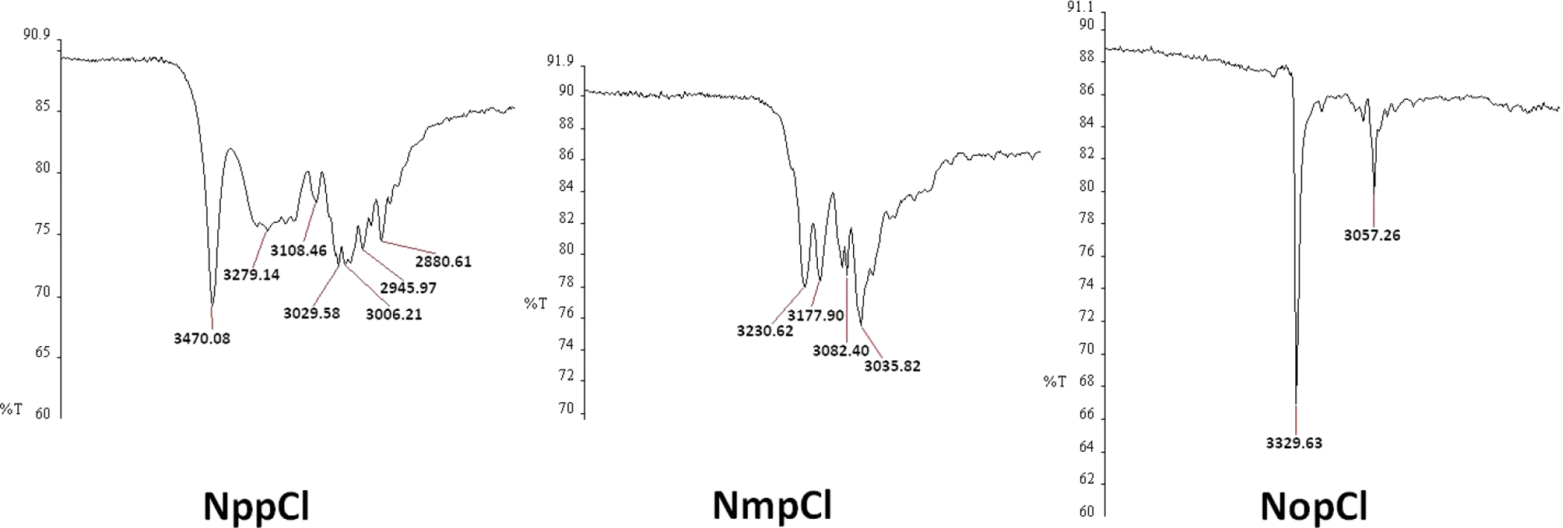
ATR-IR spectra of the **NxpCl** triad.

**Figure 8:**
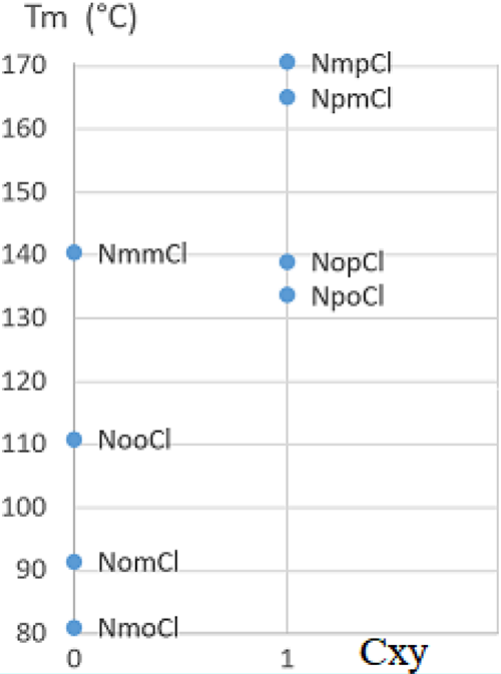
Melting point in the isomers classified according to descriptor *C*_XY_ derived from Carnelley’s rule: *C*_XY_ = 1 when x or y is *p* (*para*), *C*_XY_ = 0 (remainder).

**Figure 9:**
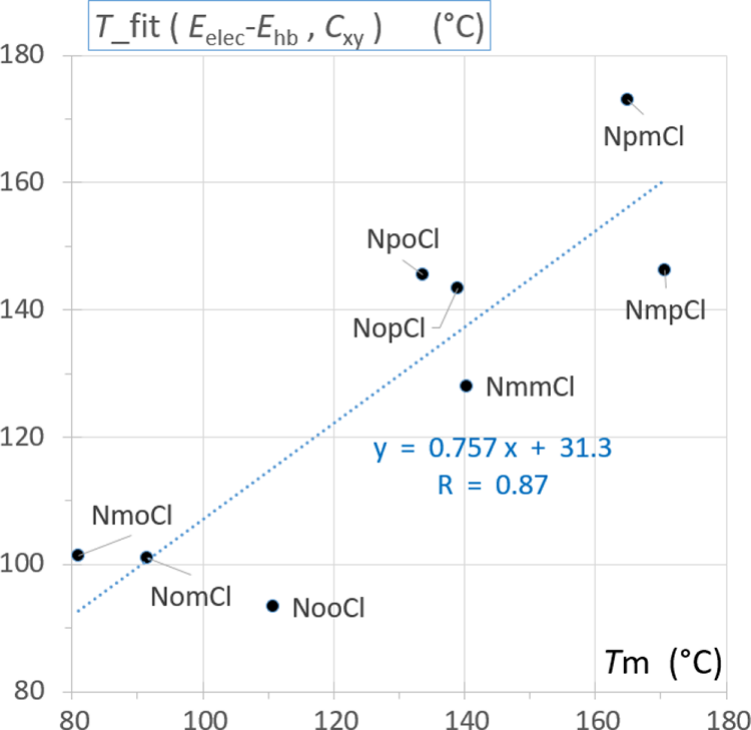
Double linear regression of the melting point *T*_m_ on the Carnelley molecule symmetry descriptor *C*_XY_ and the *E*_elec_-*E*_HB_ value, the electrostatic lattice energy diminished by the strongest hydrogen-bond electrostatic energy.

**Figure 10:**
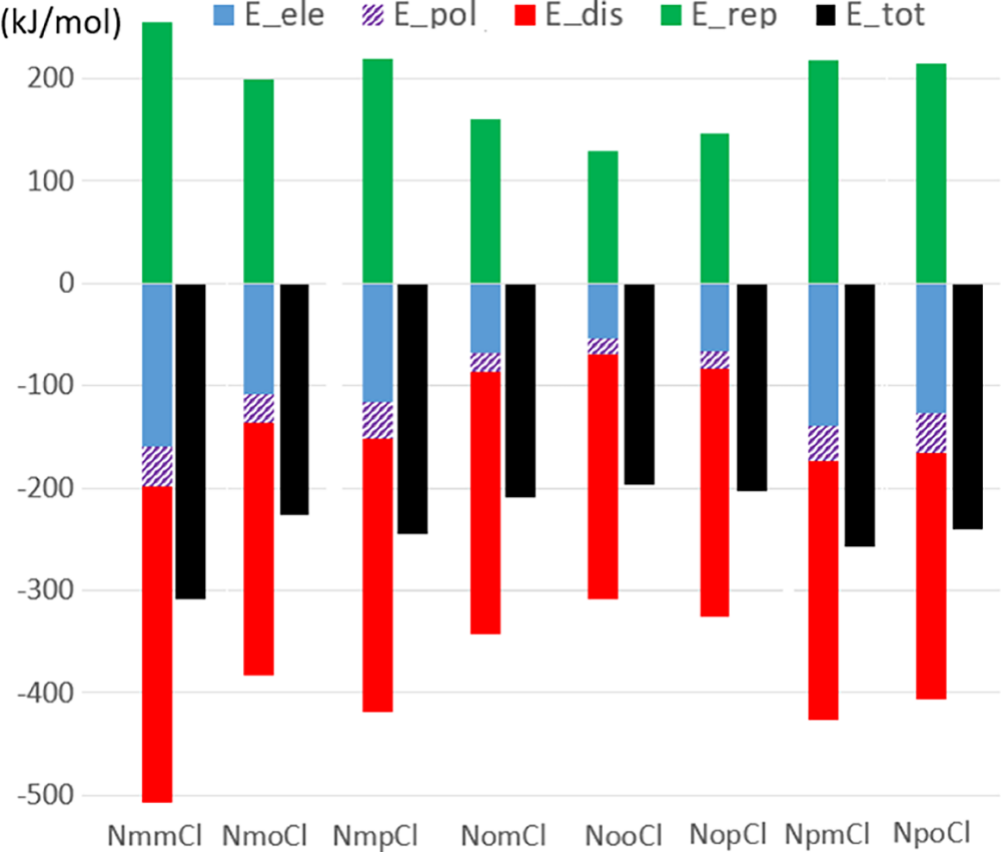
The components of the total lattice interaction energy of the **NxxCl** molecules computed on a cluster of surrounding molecules with CrystalExplorer using CE-B3LYP. B3LYP/6-31G(d,p) electron densities.^71^ In the summation of *E*__tot_ values, the electrostatic, polarization, dispersion, and repulsion components were scaled (coefficients 1.057, 0.74, 0.871, and 0.618) according to benchmarked energy models.^71^

**Figure 11:**
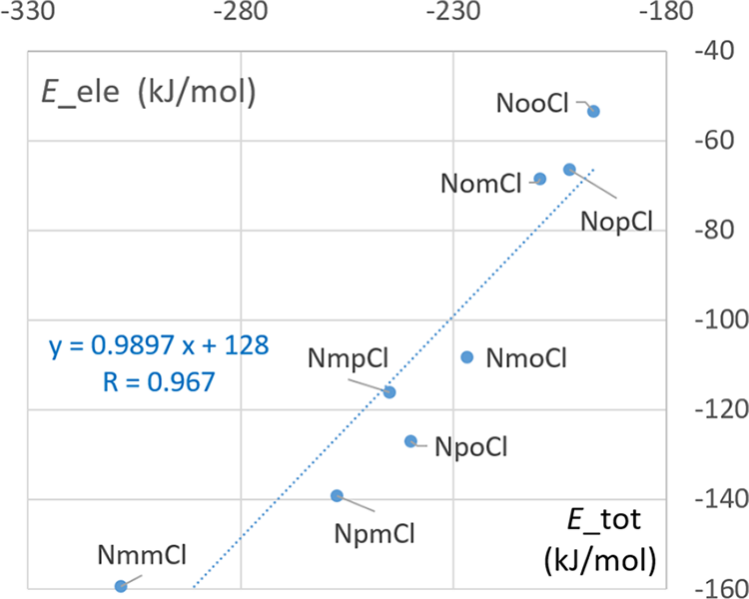
Scatterplot of total and electrostatic energy from CrystalExplorer.^71^

**Figure 12:**
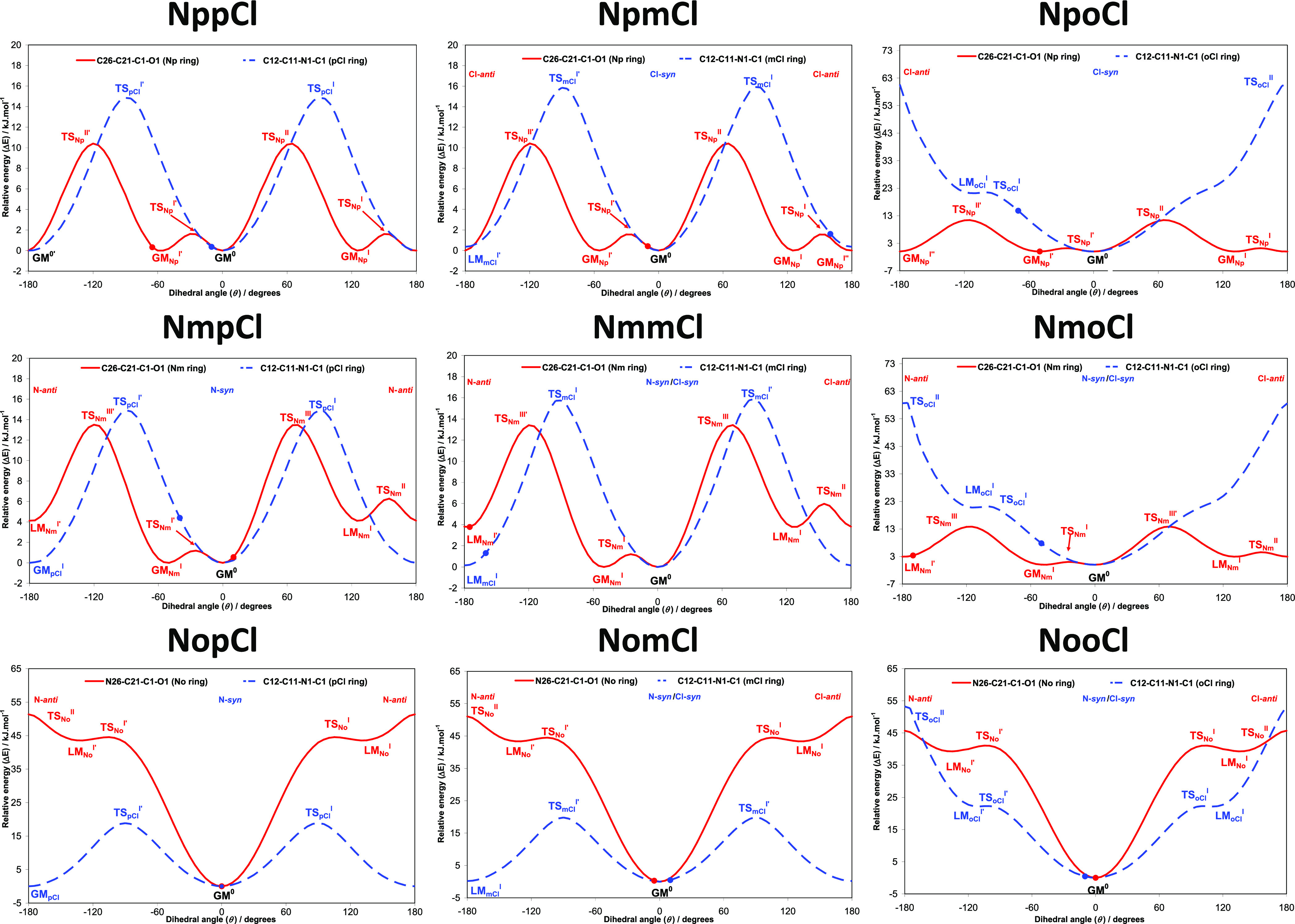
The potential energy surface (PES) conformational analysis for the **NxxCl** isomers optimized in the gas phase: the equivalent solid-state angle is depicted by (**·**). Transition states (TS) and global minima (GM) are indicated and labeled. Enlarged high-resolution figures are provided in the Supporting Information.

**Figure 13:**
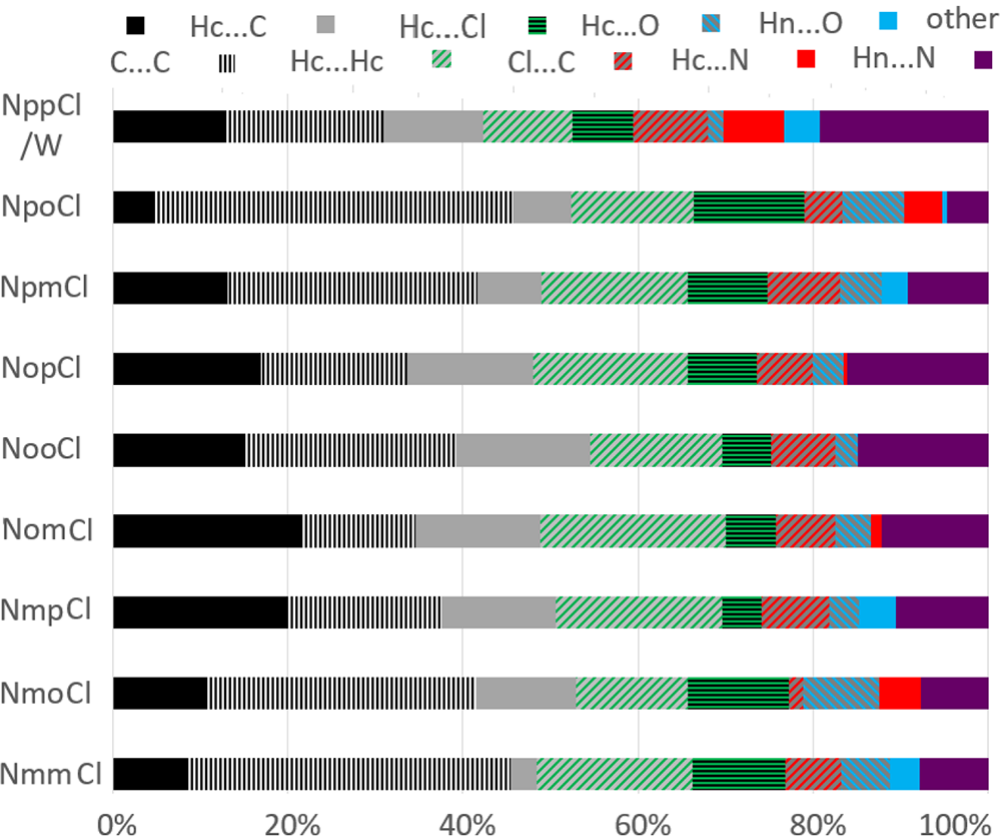
Contact proportions in the nine **NxxCl** isomer crystals.

**Scheme 1:**
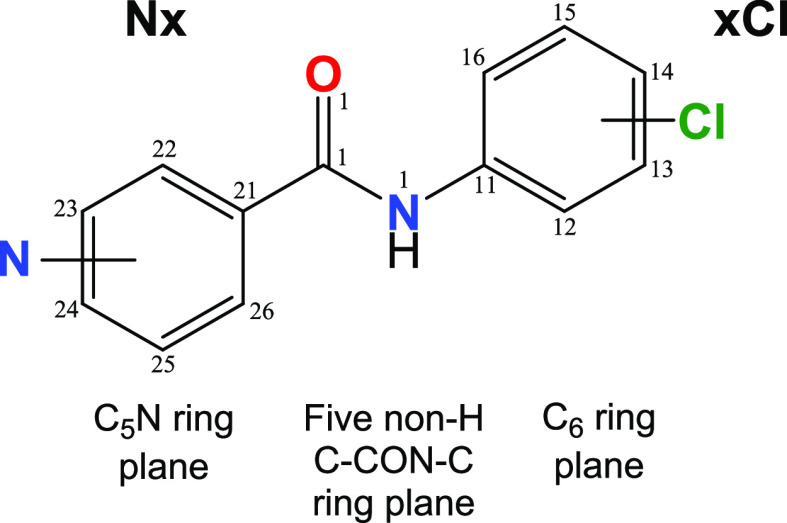
The **NxxCl** Series of Molecules with **Nx** Representing the C_5_H_4_NC=O (Pyridinoyl) and **xCl** the −HNC_6_H_4_Cl (Aminochlorobenzene) Moieties (**x** = *Para*-/*Meta*-/*Ortho*-substitutions) The numbering scheme as used in the interplanar calculations (non-H atoms only) and Figures 1–6 is shown.

We have previously reported several isomer grids of benzamides and carbamates including the mono-substituted, methyl-, fluoro-, and chlorobenzamides and the related methyl and methoxycarbamates.^50−59^ In expanding the isomer grid series, the increased numbers of compounds for analysis and for comparisons can be appreciated with what is already available for study on the CSD.^49^ In analyzing the electronic structure, one can ascertain the effects at the intramolecular and intermolecular level and derive trends and correlations in isoelectronic series of molecules such as the nine-member *N*-(chlorophenyl)pyridinecarboxamide **NxxCl** series (Scheme 1) described herein. This series is used for comparisons with related benzamide isomer grids.^50−54^ These benzamides are readily synthesized from the three *p*-/*m*-/*o*-pyridinoyl chlorides and three *p*-/*m*-/*o*-aminochlorobenzene isomers using standard synthetic and purification procedures.^50,51^ They are chemical analogues of the related *N*-(fluorophenyl)pyridinecarboxamides (**NxxF**).^50^ Nine **NxxCl** single crystal structures (Figures 1–6) and their conformational analyses and physicochemical properties are described (Figures 7–13). Together, these are analyzed and compared to highlight correlations with crystal properties and molecular charge densities and also to make notable comparisons with related series of isomers.^50−53^

## Experimental Section

### Materials, Methods, and Equipment

All chemicals, materials, vendors, and spectroscopic and crystallographic methods together with computational programs and equipment are as reported previously.^50−53^ Chemicals and silica (Davisil) were used as purchased from Sigma Aldrich; TLC alumina and silica plates were from Fluka. Melting points were measured using a Stuart Scientific SMP40 automated melting point apparatus. IR spectroscopy was recorded using a Perkin Elmer Spectrum GX FTIR spectrometer by the attenuated total reflection (ATR) method: spectral bands are quoted in cm^–1^. NMR spectroscopy was performed on a Bruker BioSpin UltraShield NMR spectrometer (293 ± 1 K) at 400 or 600 MHz for ^1^H and 100.62 MHz for ^13^C resonance. The ^1^H spectra were recorded in CDCl_3_ and DMSO-*d*_6_ with the ^13^C spectra analyzed in CDCl_3_. The NMR chemical shift values (δ) are in ppm referenced to TMS, and coupling constants (*J*) are quoted in Hz.

Single crystal X-ray data collections for the nine **NxxCl** crystal structures (Scheme 1) together with data reduction, structure solution, and refinements^60−62^ are as described for the previously reported 3 × 3 isomer grids^56,59^ and are fully detailed in the Supporting Information (Table S1). Selected crystallographic and structural information is analyzed and compiled with pertinent structural details provided in the main paper in Tables 1 and 2. Molecular structures and hydrogen bonding diagrams (Figures 1–6) are depicted with displacement ellipsoids drawn at the 30% probability level.^63,64^ The computational calculations^65−67^ were performed as described previously.^56^ Optimizations and conformational analyses in the gas phase were performed using the DFT method [B3LYP/6-311++G(d,p)].^66,67^ All calculations were performed using Gaussian09^65^ for Linux/Unix operating on an SGI Altix ICE 8200EX high-performance computing system at the ICHEC (Galway, Ireland). The gas phase data are presented in a diagram as a 3 × 3 grid to highlight trends in the position of the substituent and displayed from the **pp** to **oo** (Figure 12; in Supporting Information Section II as enlarged diagrams).

**Table 1:** Selected Crystallographic Data for **NxxCl** (Full Details Available; Table S1, Supporting Information)

structure	crystal system; space group	*Z′*	volume (Å^3^)	*R*, w*R*_2_*R*-factors,^a^ GoF
**NppCl·H_2_O**	orthorhombic; *Pbca*	1	2344.89(8)	0.042, 0.108, 1.03
**NpmCl**	monoclinic; *P*2_1_/*n*	1	1090.45(4)	0.035, 0.102, 1.07
**NpoCl**	monoclinic; *Cc*	1	1108.06(11)	0.026, 0.067, 1.10
**NmpCl**	monoclinic; *P*2_1_	1	531.27(3)	0.043, 0.114, 1.03
**NmmCl**	monoclinic; *P*2_1_/*n*	1	1068.45(4)	0.038, 0.115, 1.11
**NmoCl**	monoclinic; *P*2_1_/*c*	1	1078.89(9)	0.066, 0.149, 1.08
**NopCl**	triclinic; *P*1̅	1	543.73(3)	0.038, 0.105, 1.05
**NomCl**	triclinic; *P*1̅	1	532.00(5)	0.038. 0.124, 1.08
**NooCl**	orthorhombic; *Pbca*	1	2226.2(11)	0.051, 0.120, 0.89

a *R*-factor definitions as *R*[*F*^2^ > 2σ(*F*^2^)], *wR*(*F*^2^).^60^

**Table 2:** Salient **NxxCl** Structural Features (Interplanar Angles, Distances, and Interactions in Å or °)^a^

structure	C_6_/C_5_N (°)	C_6_/amide (°)	C_5_N/amide (°)	N···N/O^c^ (Å)	primary H bonds
**NppCl·H_2_O**	47.68(5)	7.58(7)	40.43(5)	2.831(2)^b^	hydrate packing (2 × N···O/O···O)
				2.838(3)^b^	
				2.903(2)^b^	
**NpmCl**	1.52(9)	17.96(6)	18.23(7)	3.1373(17)	amide···pyridine
**NpoCl**	83.24(7)	69.59(8)	27.43(11)	2.797(2)^c^	amide···amide
**NmpCl**	7.65(14)	32.11(10)	32.02(9)	3.079(3)	amide···pyridine
**NmmCl**	56.53(4)	30.44(4)	27.03(5)	3.0842(13)	amide···pyridine
**NmoCl**	16.30(17)	38.87(17)	25.54(15)	2.884(3)^c^	amide···amide
**NopCl**	2.86(7)	1.26(7)	1.75(7)	2.6631(16)	intra as (N–H···N)
**NomCl**	1.07(6)	7.86(5)	6.91(5)	2.6536(13)	intra as (N–H···N)
**NooCl**	8.6(2)	9.7(2)	1.2(2)	2.624(4)	intra as (N–H···N)

a C_6_ is the (C11, ..., C16) benzene plane, C_5_N is the (C21, ..., C26) pyridine ring plane, and the amide is represented by the five atom C21–C1(=O1)N1–C11 plane (Scheme 1) and with reference to Figures 1–6.

b **NppCl** monohydrate structure with N1···O1W, O1W···O1, and O1W···N1 hydrogen bonding.

c Represents N···O (amide···amide) with the intramolecular N···N interactions underlined.

The average **NxxCl** molecular volume (*i.e*., cell volume (Å^3^)/*Z*) is 273 Å^3^, discounting the **NppCl** monohydrate. The largest molecular volumes are for **NooCl** (278 Å^3^) and **NpoCl** (277 Å^3^). The smallest volumes are for the **NmpCl** and **NomCl** structures (both 266 Å^3^). The calculation for **NppCl·H_2_O** is at ∼255 Å^3^ per **NppCl**, taking into account the volume of the tightly bound monohydrate molecule (as ∼38 Å^3^).^63^

### Methods^68−74^

The electrostatic energy *E*_elec_ was computed from the charge density models transferred from the ELMAM2 database of multipolar atoms^68^ using the MoProSuite software.^69^ The structures as obtained from SHELX refinement were modified by elongation of the N–H and C–H bonds to standard distances retrieved from neutron diffraction studies.^70^ The molecules were rendered electrically neutral after charge density transfer by applying a uniform valence population shift to all atoms. The electrostatic energy between interacting molecules was obtained by the summation over pairs of multipolar charged atoms belonging to each entity. The lattice electrostatic energy was computed with the VMoPro module in real space. The energy was summed over successive parallelepiped shells surrounding the unit cell. The summations were carried over the [−9**a**,9**a**] × [−9**b**,9**b**] × [−9**c**,9**c**] space around the molecule containing 19^3^ unit cells where convergence is largely achieved.

The total energy was computed with the CrystalExplorer19 software^71^ between the asymmetric unit and a cluster of surrounding molecules within a distance of 3.80 Å. The energy components calculated within this procedure are electrostatic, polarization, dispersion, exchange-repulsion, and finally the total interaction energy. These energy calculations were performed at the B3LYP/6-31G** level of theory.^66,67^ The structures used were the same as for the electrostatic energy calculation on the multipolar model. Diagrams are included in the main paper text as Figures 8–11 and in the Supporting Information (Section IV pp 56–68) as Figures S01–S06.

## Results and Discussion

### **NxxCl** Crystal Structures

The nine *N*-(chlorophenyl)pyridinecarboxamide crystal structures (**NxxCl**) are grouped in triads for structural comparisons with pertinent structural data presented in Tables 1 and 2. Comparisons are made with the **Clxx** series^56^ (as their amide-bridge reversed isomers) together with the related **NxxF**,^50^ (methyl) **NxxM**,^51^ and **NxxBr** analogues.^54^

#### The **NpxCl** Triad

**NppCl** crystallizes as a monohydrate with the amide N–H donor, O=C, and N_pyridine_ acceptor groups engaged in hydrogen bonding interactions with the water molecule O1W. In the crystal structure, two **NppCl·H_2_O** aggregate through (_amide_N1–H1···O1W–H2W···N24_pyridine_) hydrogen bonds and form *R*^4^_4_(18) hydrogen bonded rings about inversion centers (Figure 1). The (**NppCl·H_2_O**)_2_ units are linked by 2 × (O1W–H1W···O1=C1) and 2 × (C1=O1···H1W–O1W) hydrogen bonds per aggregate. These four strong intermolecular interactions form a 2D sheet that is effectively ∼21 Å wide. Overall, 2D sheets interlock into a 3D structure by using 2 × (C13–H13···O1=C1) and 2 × (C1=O1···H13–C13) weak H-bonds per aggregate. This hydrate aggregation is similar to related benzamide hydrates with all strong hydrogen bonding donors and acceptors used (*e.g*., in **Clpm·**2**H_2_O**^75^ and **Clmm·H_2_O**(56)). The closest contacts with the *para*-chlorine Cl14 atom involve three H atoms (H23, H25, and H26) on symmetry related molecules but with all of the H···Cl14 distances larger than 3.0 Å.

### **NpmCl** and **NpoCl** Structures: Isomorphous Behavior^49,76−83^

**NpmCl** is isomorphous with **NpmM**(51) and **NpmBr**(54) in the monoclinic space group *P*2_1_/*n* but is not isomorphous with **NpmF**(50) (see below). The hydrogen bonded N–H···N chains in **NpmCl** contrast with conventional N–H···O=C (amide···amide) interactions in **NpoCl**. Furthermore, two C–H···O=C contacts are noted in **NpmCl** in the absence of N–H···O=C interactions (Figure 2). The amide···pyridine N–H···N_pyr_ hydrogen bonded chains are augmented by two weaker C–H···N_pyr_ interactions. There are up to six Cl···H–C close contacts at *d*_HCl_ < 3.6 Å with symmetry related molecules, though the shortest distance, H15···Cl13, is larger than 3.2 Å. In **NpoCl**, amide···amide hydrogen bonding as 1D chains along the *c*-axis direction is the primary interaction mode (Figure 3). Chains are weakly linked by C–H···N_pyridine_ contacts. **NpoCl** is isomorphous with both **NpoM**(51) and **NpoBr**(54) in space group *Cc* but differs slightly from the **NpoF** and **NpmF** structures where N–H···N interactions dominate. However, both **NpxF** structures also crystallize in space group *Cc* and the series of structures can be considered as being on the continuum of isomorphic behavior.^49,50,52^ In **NpoCl**, the closest contacts between the chlorine Cl12 and H atoms involve H13 on a symmetry related molecule (though with H13···Cl12 > 3.1 Å). Therefore, in summary, both **NpmCl** and **NpoCl** exhibit an isomorphous behavior with their methylated (**M**)^51^ and brominated (**Br**)^54^ congeners but not with their fluorinated (**F**) analogues.^50^

#### The **NmxCl** Triad

The **NmxCl** triad structures are not isomorphous with any of their **NmxF**(50) and **NmxM**(51) congeners, although there is an isostructural relationship between **NmmCl** and **NmmF**. **NmpCl** aggregates by *zig-zag*_amide_N–H···N_pyridine_ chains of interactions along the *b*-axis direction in the monoclinic space group *P*2_1_ and forms a 2D herringbone structure ∼16.5 Å wide (Figure 4). Chains are linked by C–H···O=C interactions and form a rumpled sheet. Short C22–H22···C22^27,29^ interactions form relays of contacts in tandem with _amide_N–H···N_pyridine_. The Cl14 atoms are not involved in any strong hydrogen or halogen bonding and are positioned in the lattice while involved in multiple aromatic H atom contacts. The closest contact involves the Cl14 and H25 atoms on symmetry related molecules (with H25···Cl14 at ∼3.0 Å).

**NmmCl** with 1D *zig-zag* N–H···N chains is (at least) isostructural with **NmmF** in space group *P*2_1_/*n*. Aggregation is assisted by the alignment of 1D chains *via* C14–H14···O1 interactions and formation of 2D sheets (Figure 5). In doing so, series of tetrameric units are generated in the **NmmCl** crystal structure and with C–H···π(arene) interactions generate ruffled sheets. In contrast, **NmoCl** is isomorphous with **NmoBr**(84) (**TICDOZ01**)^49^ with N–H···O=C intermolecular interactions along the *b*-axis direction and short intramolecular interactions between the *ortho*-Cl12 and the N–H group. In addition, there are Cl12···C14 contacts between the *ortho*-Cl12 and symmetry related chlorinated aromatic rings.

#### The **NoxCl** Triad: Relatively Planar Molecules with Aromatic Stacking^32^

All three **NoxCl** have their benzene and pyridine rings aligned close to co-planarity (Table 2); this is largely influenced by two intramolecular N–H···N and C–H···O interactions. Both **NopCl** (reported previously as **GEPQIC**)^85^ and **NooCl** are isomorphous with the **NopBr** and **NooBr** congener structures,^54^ respectively.^83^ The **NopCl** crystal structure with an intramolecular N1–H1···N22_pyridine_ contact has aromatic stacking and long-distance C–H···O/Cl interactions (*d*_HCl_ = 2.88 Å) resulting in 2D sheet formation. Likewise, **NomCl** has two intramolecular H-bonds per molecule: the short N1–H1···N22 and a weaker C–H···O contact. Consequently, there are no strong intermolecular hydrogen bonds but only two C–H···O and one C–H···Cl (*d*_HCl_ ∼2.90 Å) weak H-bonds; the closest C···C aromatic stacking distance is 3.4873(17) Å.^32^**NooCl**, isomorphous with **NooBr**,^54^ is relatively planar due to the intramolecular Cl22···H1(N1)···N22 bifurcated hydrogen bonding arrangement (Figure 6) and is similar in structure to **NooF**,^50^**NooM**,^51^ and **Cl-NooM** (a side-product from the **NooM** synthesis).^51^ The intermolecular interactions are typically weak and comprise C–H···O and C–H···π(arene) contacts (with C···C aromatic stacking distances ≥3.60 Å). Overall, the relatively planar **NoxCl** triad compares well with the **NoxM**, **NoxF**, and **Cl-NoxM** series, and in each of these series, it is usually the *para*-derivative that has its arene rings twisted most from co-planarity.^50,51^

### Isomorphous Relationships: Summary and Analysis of **NxxCl** and **NxxBr**(18,49,54,83–87)

Isomorphous relationships between structures in the 3 × 3 isomer grids show an overlap between five **NxxCl** isomers and their **NxxBr** analogues (as the **NpmCl**/**Br**, **NpoCl**/**Br**, **NmoCl**/**Br**,^84^**NopCl**^85^/**Br**, and **NooCl**/**Br** pairs). This correlates well with what has been noted with five of the **Clxx**/**Brxx** amide-bridge reversed analogues (see Table 3).^54,56^ Furthermore, for the ’**pm**’ or ’**po**’ sets of crystal structures, the methylated analogues **NpmM** and **NpoM** are isomorphous with the **Cl**/**Br** pairs and further extend the structural series overlap.^54^

**Table 3:** Isomorphous Relationships between **NxxCl** (this work) and **NxxBr**:^54,84^ Comparisons with the Amide-Bridge Reversed **Clxx**(56) and **Brxx**(54,83)^a^

**NxxCl** and **NxxBr** isomer grids			**Clxx** and **Brxx** isomer grids		
**NxxCl**	space group	**NxxBr**(54)	**Clxx**(56)	space group	**Brxx**(54)
NppCl**·**H_2_O	*Pbca* ≠ *P*2_1_	NppBr	**Clpp**	*P*2_1_/*c*	**Brpp**
**NpmCl**	***P*2_1_/*n***	**NpmBr**	Clmp (*Z*′ = 4)	*P*1̅ ≠ *P*1̅	Brmp (*Z′ =* 2)
**NpoCl**	***Cc***	**NpoBr**	**Clop**	***Pbca***	**Brop**
NmpCl	*P*2_1_ ≠ *C*2/*c*	NmpBr	Clpm	*P*1̅ ≠ *C*2/*c*	Brpm
NmmCl	*P*2_1_/*n* ≠ *P*1̅	NmmBr	**Clmm·H_2_O**	*P*2_1_/*c*	**Brmm·H_2_O**
**NmoCl**	***P*2_1_/*c* –***P*2_1_/*a*	**NmoBr**(84)	**Clom**	**C**2*/*c**	**Brom**
**NopCl**(85)	****P**1̅**	**NopBr**	Clpo	*C*2/*c* ≠ *P*1̅	Brpo
NomCl	*P*1̅ ≠ *C*2/*c*	NomBr	Clmo	*P*1̅ ≠ *P*2_1_/*c*	Brmo
**NooCl**	***Pbca***	**NooBr**	**Cloo**	**C**2/**c**	**Broo**

a Isomorphous pairs are highlighted in bold with their common space group in italics. The ≠ symbol is for crystal structures that are not isomorphous; for **Clmp** and **Brmp**, *Z*′ is also noted. **Clxx** and **Brxx** are understood as **ClxxN** and **BrxxN** but are noted this way in refs (56, 54).

In Table 3, 10 of the 18 structural pairs from the **Clxx**/**Brxx** and **NxxCl**/**NxxBr** isomer grids are isomorphous.^82,83^ These results support an extensive Cambridge Structural Database (CSD) study by Mukherjee and Desiraju^18,49^ where they noted a significant degree of similarity between pairs of structures presenting C–**X** bonds (**X** = Cl or Br).^18^ Such pairs are observed to adopt the same space group, number of molecules in the unit cell, and reduced unit cell parameters (within 1 Å). Using this, our study aimed to compare the **Clxx** grid^56^ with the **NxxCl** series and make structural comparisons with the **Brxx** and **NxxBr** analogues.^54^

The extent of isomorphous behavior between pairs of structures in the **Cl**/**Br** series is much greater than that noted for the **Me**/**F** or **F**/**Cl** analogous pairs (**NmmF** and **NmmCl** are isostructural in *P*2_1_/*n*).^50,51,54^ Moreover, there are examples where three structures exhibit an isomorphous behavior; *e.g*., the **NpmM**, **NpmCl**, and **NpmBr** triad is isomorphous in the monoclinic space group *P*2_1_/*n*. Furthermore, **NpoM**, **NpoCl**, and **NpoBr** are isomorphous in space group *Cc* and are aggregating by amide···amide interactions.^50,51,54^ However, **NpoF** (and **NpmF**) differs in structure using amide···pyridine interactions, though also crystallizing in space group *Cc*.^50^ Of note are the isostructural **Clmp** (*Z*′ = 4) and **Brmp** (*Z*′ = 2) with two sets of similar unit cell axes in **Clmp** (*a*, *b*) and **Brmp** (*b*, *c*) and with the third axis (*a*) halved in **Brmp** (Supporting Information Table S4b). This represents the extent of overlap within these classes of functionalized benzamides.^52−54^ There is an extensive ’**pp**’ series with several closely related crystal structures.^52,56^ There are, however, no pairs of isomorphous **NxxCl**/**Clxx** structures involving amide-bridge swapped isomers,^88,89^*e.g*., **NomCl**/**Clmo**, as noted in the **NmmM** and **Mmm** crystal structures.^51,53^ Ojala and co-workers have commented on bridge-flipped isomers in an extensive series of benzylideneanilines and phenylhydrazones.^88,89^ In our series, the amide group N–H dominates as a pivot in the crystal structures and together with the N_pyridine_ and halogen **X** reduces the possibility of bridge-flipping or amide-bridge swapping.^50,51,54^

The general trend for **Cl**/**Br** pairs of isomorphous structures is interesting,^18,49^ and in some cases, a methyl analogue (**NxxM**)^51^ is isomorphous with the **Cl**/**Br** pairs (**NmoCl**/**NmoBr**;^84^**NmoM**/**NmoBr**^86^ with **NmoBr** polymorphs^84,86^^,^^87^). However, it is also notable that the fluorinated **Fxx**(34,52)/**NxxF**^50^ do not tend to form isomorphous relationships with **Me**, **F**, or **Cl** to the same extent as the **Clxx**(56)/**Brxx**^54^ and **NxxCl**^this work^/**NxxBr**^54^ groups of structures.

### Structural Aspects of Organic Chlorine

Fluorine has been extensively analyzed in terms of intermolecular interactions and contacts by using the CSD and other analytical methods.^33−36,49,90,91^ Chlorine contrasts with fluorine as it is often present as a chlorinated solvent such as a CH_2_Cl_2_ or CHCl_3_ solvate in crystal structures.

Analysis of C–H···Cl intermolecular interactions in molecular crystals as a function of the hybridization of the donor atom and acceptor atoms shows the C_(sp2)_–H···Cl–C_(sp2)_ to be prevalent. Furthermore, upon cone correction, this type of C_(sp2)_-H···Cl intermolecular interaction exhibits a clear preference for angularity of ∼120° with the area approaching linearity also dominant. Analysis of N–H···Cl–C and O–H···Cl–C intermolecular interactions shows that they are less common than C–H···Cl–C interactions based on a statistical analysis as noted by the decrease in observed ’hits’.^49^ This was also noted in the analysis of several families of halogenated organic molecules by the contact enrichment ratio^91^ that confirmed that organic halogen atoms prefer to interact with the lowly charged H_C_ hydrogen atoms (bound to a carbon atom) rather than with H_O_ atoms (bound to O). On the other hand, O and N atoms that are stronger H-bond acceptors tend to form H-bonds with the more polar H_N_ and H_O_ hydrogen atoms.

The methodology described could be extended to investigate intermolecular interactions involving various other halogenated organic molecules containing bromine or iodine atoms. This is expected to provide a deeper understanding into the nature of such contacts and into the characteristics of interactions as a whole. Such analyses should be viewed in tandem with the structural similarity approach used by Mukherjee and Desiraju in their in-depth CSD study.^18,49^

### Infrared Analysis

**Scheme 2:**
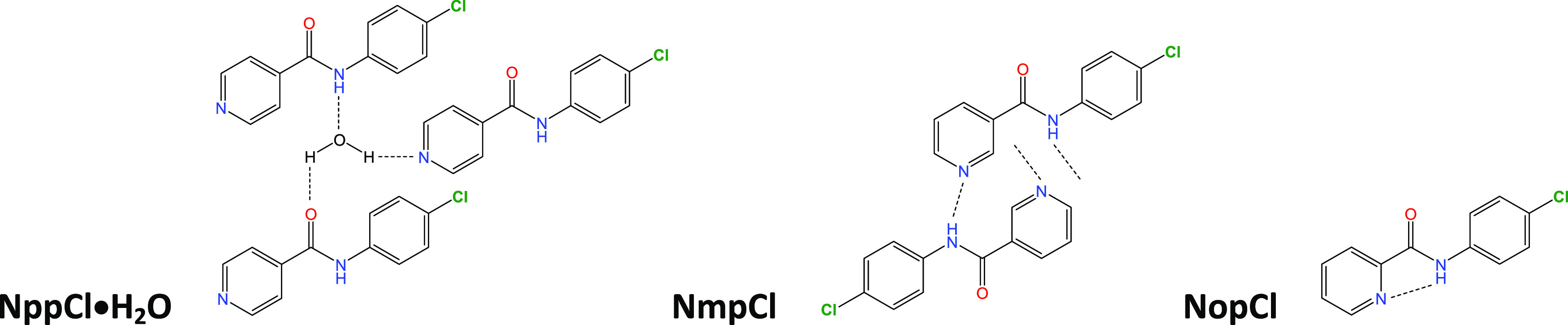
Intermolecular Hydrogen Bonding in **NppCl·H_2_O** and **NmpCl** and Intramolecular Hydrogen Bonding in **NopCl**

The ATR-IR spectra of all **NxxCl** derivatives can be correlated with their solid-state structures. For example, in comparison of the **NxpCl** spectra (Figure 7; Supporting Information p 55, ATR-IR diagram), three distinct spectra are observed as would be expected from calculated results. Indeed, **NppCl·H_2_O** forms N–H···O–H···O=C and O–H···N_pyridine_ intermolecular hydrogen bonds involving **NppCl** and the water molecule (Scheme 2). Its spectrum contains a band at 3470 cm^–1^ indicating the water of crystallization (in the crystal structure as **NppCl·H_2_O**).^75^

As expected, the **NppCl** molecule has two potentially strong acceptor groups, N_pyridine_ and amide C=O, with one donor N–H group. It interacts with water having two potential O–H donors and either one or two acceptors as the O atom electron lone pairs. This effectively balances the total number of donors/acceptors in the crystal structure of **NppCl·H_2_O**.

Two distinct IR bands at 3231 and 3178 cm^–1^ in the **NmpCl** spectrum reveal an intermolecular hydrogen bond. This is as expected from the crystal structure results for the catemeric N–H···N_pyridine_ chains arising in **NmpCl** (Figure 4). This is further highlighted for the **NoxCl** triad due to the presence of an intramolecular N–H···N_pyridine_ interaction. In the **NopCl** spectrum, the very sharp strong band at 3330 cm^–1^ indicates that there is no strong intermolecular hydrogen bonding. For the **NoxCl** triad, the spectrum is indicative of an intramolecular hydrogen bond (Scheme 2) as noted in the **NoxF**(50) and **NoxM**(51) triads. Indeed, as shown in the Supporting Information (ATR-IR figure), there is a high degree of correlation between the interactions in the nine **NxxCl** crystal structures and their respective ATR-IR spectra. In structures with similar primary hydrogen bonding, *e.g.,***NoxCl**, the ATR-IR spectra show similar features.

### Melting Point Analysis^92−99^

Comparisons between the **Clxx** and **NxxCl** melting points are essential especially where there are structural relationships between the two series of isomers (Table 4). In previous work on related systems (**NxxF**, **NxxM**, **Fxx**, and **Mxx**),^50−53^ it has been shown that there is a general adherence to Carnelley’s rule that relates higher molecular symmetry and increased melting points.^50−54,56^

**Table 4:** Melting Point Ranges (°C) of the **NxxCl** (this work) and **Clxx** Isomers^56^,^a^

**NxxCl**	**NpxCl**	**NmxCl**	**NoxCl**
**NxpCl**	139.0–140.0 (**W**)	168.3–172.9	138.0–140.0
**NxmCl**	164.0–166.0	139.2–141.6	90.8–92.1
**NxoCl**	132.7–134.7	80.0–81.9	110.1–111.5

**Clxx**	**Clxp**	**Clxm**	**Clxo**
**Clpx**	206.2–208.4	150.1–151.3	131.5–134.5
**Clmx**	185.4–187.2	112.4–113.9 (**W**)	95.0–105.0
**Clox**	167.9–169.9	134.5–137.8	134.4–138.0

a Monohydrates are marked as (**W**).

The **Clxx** series provides an illustration of Carnelley’s rule^93−95,98^ (Table 4).^56^ An empirical function based on substituent positions and the lattice electrostatic energy was introduced and allowed a multilinear fit of the melting temperatures yielding a correlation coefficient with experimental values larger than 95%. The correlation coefficient between the melting points of **Clxy**(56) and **NxyCl** series is 51% (with **x**, **y** indicating *o*-/*m*-/*p*-substitution). Given the high degree of correlation, this model can be further refined in series of related benzamides and its possible predictive behavior evaluated.

The **NxxCl** melting points have been measured in a similar fashion to previous measurements and also independently compared using a blind test. Of interest in Table 4 is that the average **NxxCl** melting point is 130 °C, and this is ∼20°C less than the corresponding amide-bridged reversed **Clxx** isomers.^56^ How does this difference in melting points arise for isomers that differ by so little (as amide-bridge reversed structures)? The highest melting points are for **NmpCl** (170 °C) and **NpmCl** (165 °C), and the lowest are for **NmoCl** (68 °C) and **NomCl** (91 °C). The **NppCl** crystal as a monohydrate is kept separate and recorded for the sake of completion. The observed trends are what would be expected from molecular symmetry based on Carnelley’s rule^93^ and similar to our related series.^50−54^

As seen previously in the **Clxx** series^56^ (average melting point of 148 °C), the effect of chlorine substitution (compared to fluorine or methyl) is to bestow an average higher melting point of 17 °C compared to **Fxx** (131 °C), which is 15 °C greater than **Mxx** (116 °C) (in a trend of Br ≈ Cl > F > Me).^93−98^ Overall, the **Clxx**,^56^**Fxx**,^52^ and **Mxx**(53) series have higher average melting points than their corresponding amide-bridge reversed **NxxCl** (130 °C), **NxxF** (117 °C),^50^ and **NxxM** (113 °C)^51^ isomer grids. One partial answer must lie in the presence of intramolecular N_pyr_···H–N_amide_ hydrogen bonds in **NoxCl** structures. The equivalent but weaker Cl_pyr_···H–N_amide_ hydrogen bonds are not formed in the **Clox** structures. The average *T*_m_ is 114 °C for **NoxCl** and 123 K for **Clox**.^56^ Globally, these subsets of structures with intramolecular H-bonds have lower melting points than their **NmxCl** and **Clmx** counterparts that have the same molecular symmetry level (Table 4). The presence of the intramolecular H-bond results in weaker intermolecular interactions and electrostatic energy, and consequently, *T*_m_ is decreased, as discussed in the next paragraph. The rest of the answer must lie in intramolecular interactions and how the molecules pack in their respective crystal structures.

### Melting Points and Electrostatic Energy

To relate the melting point temperatures (*T*_m_) to energies, additional analyses were conducted to identify correlations. The **NppCl·**H_2_O crystal structure, which has a different chemical content, was not included in the analysis.

The Gibbs free energy of a system depends on the temperature *T* and the enthalpy (Δ*H*) and entropy (Δ*S*) variations: Δ*G* = Δ*H* – *T*Δ*S*. The free energy of melting vanishes at the temperature *T*_m_, and therefore,
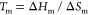
1

According to eq 1, the melting point temperature is expected to increase when the enthalpy change Δ*H*_m_ is large. The crystal enthalpy is closely related to the computed lattice energy (the mechanical energy to separate the molecules to infinity while keeping their crystalline electron distributions and their nonrelaxed geometry). The electrostatic component (*E*_elec_) can be estimated directly using the multipolar atom model transferred from the ELMAM2 electron density database.^68^ The relationship between *T*_m_ and the lattice or electrostatic energy is investigated here.

To see some trends, Table 5 shows the correlation between the melting points and several energetic and molecular symmetry descriptors of the nonhydrated **NxxCl** crystals. The quantities *T*_m_ and −*E*_elec_ indeed show a small correlation (*R* = 37%) in Figure S01 (Supporting Information). The three **NoxCl** compounds, with the intramolecular N–H···N hydrogen bond, have the weakest *E*_elec_ values. In our previous study of **Clxx** isomers,^56^ the two properties showed a higher correlation of *R* = 0.47, and the compounds with the strongest electrostatic lattice energy tended to have the highest melting points.

**Table 5:** Correlation Coefficients between the Experimental *T*_m_ Values and Computed Properties^a^

*C*_xy_ (Carnelley’s rule)	0.76
–*E*_elec_	0.37
–(*E*_elec_-*E*_HB_)	0.44
–*E*__tot_	0.46
–(*E*__tot_-*E*_HB_)	0.64
*T*__fit_(*E*_elec_, *C*_XY_)	0.807
*T*__fit_(*E*_elec_-*E*_HB_, *C*_XY_)	0.870
*T*__fit_(*E*__tot_, *C*_XY_)	0.887
*T*__fit_(*E*__tot_-*E*_hb_, *C*_XY_)	0.873
*T*__fit_(*E*__tot_, *E*_hb_, *C*_XY_)	0.888

a Correlations of *T*_m_ with *T_*_fit_ melting points fitted by multiple regression are shown.

In the **Clxx** isomer series, it was observed that *E*_HB_, the electrostatic energy between acceptor and donor atoms of the strongest hydrogen bond in the crystal, has an influence on the melting point. The *T*_m_ values were more correlated (*R* = 0.63) with the −(*E*_elec_-*E*_HB_) values than by considering −*E*_elec_ exclusively. This suggested that contributions to Δ*H*_m_ are rather due to the weaker intermolecular interactions, as the strongest hydrogen bonds might subsist in the molten phases. The (*E*_elec_-*E*_HB_) quantity refers to the total electrostatic energy corrected by removing the strongest hydrogen bond contribution. In the **NxxCl** series as presented herein, this correlation *R* = 0.44 is more moderate but still stronger than *R*(*T*_m_,-*E*_elec_) = 0.37 (Table 5).

Entropy is another key factor that plays a significant role in the melting point temperature in eq 1. Hence, according to Carnelley’s rule,^92,93^ a molecule with a higher rotational symmetry is expected to show a smaller increase in entropy */*Δ*S*_m_ when the crystal melts and, consequently, an increased *T*_m_ temperature.

The *para-*substituted **NxxCl** compounds have a higher symmetry than the unsymmetrical *ortho-* and *meta-*substituted isomers. In Figure 8, the compounds with a *para*-substitution clearly show, on average, higher *T*_m_ values than the other isomers (105.9 *vs* 152.1 °C). To model Carnelley’s rule by accounting for its dependence on the substituent positions, the *C*_XY_ descriptor was defined for the **NxyCl** isomers: *C*_XY_ = 1 when one of the substitution positions is located as *para*; *C*_XY_ = 0 when there is no *para*-position. The resulting correlation between *T*_m_ and *C*_XY_ is 0.76.

A double linear regression to fit *T*_m_ against the Carnelley-derived *C*_XY_ function and the lattice energy was also undertaken. This model accounts simultaneously for the enthalpic and the entropic contributions to the melting point *T*_m_. The scatterplot of the experimental *T*_m_ and of the ones fitted from (*C*_XY_,*E*_elec_-*E*_HB_) data shows a correlation of 0.87 (Figure 9), which is lower than the high value of *R* = 0.961 observed for the **Clxx** benzamide series.^56^ The same double regression using (*C*_XY_,*E*_elec_) properties leads to a fit of lower quality at *R* = 0.81 (Figure S02 Supporting Information). As observed also for the **Clxx** series,^56^ when the *E*_elec_ and *C*_XY_ properties are combined, taking into account the *E*_HB_ energies of the strongest H-bond as a third variable does not significantly improve the linear fitting (Table 5).

The total lattice energy *E*__tot_ and its components have been computed with CrystalExplorer and are shown in Figure 10.^71^ The electrostatic energy *E*__ele_ as derived from CrystalExplorer^71^ and *E*_elec_ derived from the ELMAM2^68^ electron density database show an excellent correlation (*R* = 0.967) (Figure 11), but the former values are on average 25% higher than the ELMAM2-derived ones (Figure S03).^68^ For the **NxxCl** series, the average electrostatic *E*__ele_ (from CrystalExplorer^71^) and dispersion *E*__disp_ values are −105 ± 38 and −257 ± 23 kJ/mol, which show that most of the lattice energy comes mostly from the dispersion component. This is related to the mostly hydrophobic character of the **NxxCl** molecules. The *E*__disp_ values show however low variations among the compounds, and as a result, the ranking of the *E*__tot_ values originates mostly from differences in *E*__ele_ values. This is confirmed by the scatterplot as depicted in Figure S03 (Supporting Information), which shows globally increasing *E*__tot_ values as *E*__ele_ is augmented. The *E*__tot_ values can be approximated from the *E*__ele_ ones by a well-defined linear equation that has a slope close to unity and has an intercept value of approximately 128 kJ/mol. The *T*_m_ melting points are much more correlated with the total energy −*E*__tot_ and −(*E*__tot_-*E*_HB_) (*R* reaching 0.64) compared to the equivalent values issued from electrostatic energy −*E*_elec_ (Table 5; Figures S04 and S05; Supporting Information). The double linear fit of *T*_m_ on *E*__tot_ and *C*_XY_ values yields a high *R* = 0.887 value (Figure S06; Supporting Information).

### *Ab Initio* Modeling Studies and Conformational Analysis of the NxxCl Isomer Grid

The molecular model geometries of the **NxxCl** isomers have been investigated and *ab initio* geometry optimizations undertaken using the DFT method (B3LYP/6-311++G(d,p)) with the Gaussian09 software.^65^ The three resulting optimized torsion angles α, β, and δ are tabulated in Table 6.

**Table 6:** Torsion Angles (°) of the Optimized **NxxCl** isomers^a^

	α (°)	β (°)	δ (°)
**NppCl**	23.71	4.24	1.47
**NpmCl**	25.72	4.97	1.39
**NpoCl**	23.27	3.61	2.20
**NmpCl**	23.13	4.93	2.26
**NmmCl**	22.93	4.23	2.14
**NmoCl**	21.42	4.15	2.73
**NopCl**	0.00	0.00	0.00
**NomCl**	0.00	0.00	0.00
**NooCl**	0.00	0.00	0.00

a Angle C26–C21–C1=O1 (**N-ring**) refers to α, C1–N1–C11–C12 (or **Cl-ring**) refers to β, and the O1=C1–N1–C11 amide linkage is the δ angle. All geometries are based on B3LYP/6-311++G(d,p) optimization in the gas phase.^66,67^ The **NppCl** optimization was undertaken on the molecule but not the hydrate.

The optimized geometries of the nine **NxxCl** isomers (Table 6) closely resemble the geometries of their equivalent isomer grids, *i.e.,***NxxF** and **NxxM**.^50,51^ The **NoxCl** triad is completely planar with all torsion angles at 0.00°; the planarity of the **NoxCl** triad is assumed by the intramolecular N1–H1···N22 interaction.^50,51^ On the other hand, the **NpxCl** and **NmxCl** triads have torsion angles more or less deviating from planarity. On average, the α angle (*para-*/*meta*-pyridinyl ring, **N-ring**) is 23.36° (σ = 1.27°), whereas the β torsion angle (chlorophenyl ring, **Cl-ring**) is 4.36° (σ = 0.47°) and the δ torsion angle (amide linkage) is 2.03° (σ = 0.47°).

The conformational analysis was undertaken using the B3LYP/6–311++G(d,p) method and basis set.^66,67^ The PES conformational analysis diagrams (Figure 12) for the 3 × 3 **NxxCl** isomer grid reveal a significant similarity with their related **NxxF** and **NxxM** series.^50,51^ The N-ring and most of the Cl-ring PES profiles are similar with rotational barriers having comparable heights. However, the *ortho*-chlorophenyl ring (**oCl**-ring) shows a higher rotational barrier (53–60 kJ/mol) as compared to the **oM**-ring (35 kJ/mol) (**NxoM** triad)^51^ and the **oF**-ring (50 kJ/mol) (**NxoF**).^50^ This is rationalized by factoring in the larger atomic radius of chlorine compared to fluorine or the methyl group. Other differences are for **NxoM/F/Cl** triads and the effect of the *ortho*-methyl group on the shape and height of the β torsion angle C1–N1–C11–C12(Me) compared to both F and Cl that can be explained on both steric (size) and electronic grounds (intramolecular hydrogen bonding involving F and Cl).

**Scheme 3:**
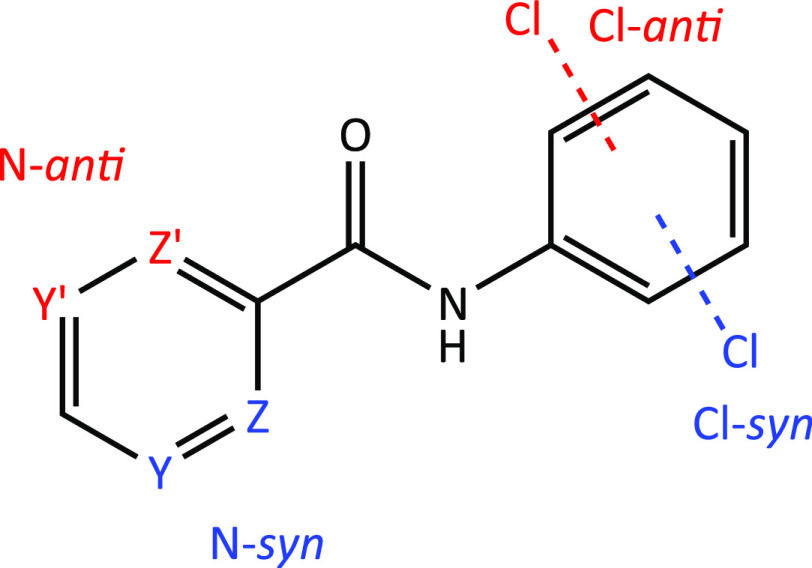
Possible Conformations of **NxxCl** as Applied to the *Ortho*-/*Meta*-substitutions

Conformational analysis suggests that the **N*-syn*** conformation (Scheme 3) of the N-ring is more stable (by 3.9 kJ/mol) while the **mCl**-ring is just slightly stable (by 0.2 kJ/mol), making the **Cl-*anti*** conformation a possibility (Scheme 3). In addition, the **N-*anti*** conformation is plausible but is less probable in the gas phase. The *ortho***oN**-ring and **oCl**-ring can be stable only when they are positioned in the ***syn*** conformation. In summary, all modeling predictions are consistent with our previous studies on the **NxxF** and **NxxM** series.^50,51^

### Comparisons of Calculated Models with Solid-State Structures

Differences between the modeled and solid-state torsion angles (**N-ring** and **Cl-ring**) are marked with a dot (**·**) on each of the **NxxCl** PES curves (Figure 12). The solid-state conformations of the **NoxCl** triad match those of the modeled structures with little or no torsion angle deviation. The **NoxCl** molecules are planar in both the solid state and as models, *e.g.,***NooCl** (Figure 6). Therefore, the N1-H1···N22 intramolecular hydrogen bond and molecular planarity established in the optimized *ab initio* models is confirmed by the **NoxCl** solid-state structures. In **NomCl**, the **Cl-*syn*** conformation is preferred over **Cl-*anti***; the **Cl-*anti*** would represent a disruption of the intramolecular hydrogen bonding, while the **Cl-*syn*** assists in the formation of C23–H23···Cl13 intermolecular interactions.

In the **NpoCl** crystal structure, the **oCl**-ring deviates by −70° from the optimized model. This deviation is necessary to allow for the formation of amide···amide (N1–H1···O1 = C1) intermolecular hydrogen bonds (Figure 3). The chlorine is positioned favorably, while the twisted **oCl**-ring allows for a closer aggregation of **NpoCl** molecules and hydrogen bond formation. A similar rotation of the **oCl**-ring arises in **NmoCl** where a less pronounced change in the Cl-ring torsion angle assists in N1–H1···O1 hydrogen bond formation. In tandem, the N-ring adopts the **N-*anti*** conformation that is essential for the formation of both C14–H14···Cl12 interactions and other important contacts that assist in structure aggregation.

Both aromatic rings in **NmmCl** are in the **N-/Cl-*anti*** conformation and opposite to the modeled gas phase structure (Figure 5). The flipping of the N-ring into an **N-*anti*** conformation was already noted in **NmmF**(50) and **NmmM**(51) as it is critical for the formation of N1–H1···N23 hydrogen bonds and *zig-zag* chains. It is unclear why the Cl-rings in both **NmmCl** and **NpmCl** isomers are in a slightly less stable geometry by adopting the plausible **Cl-*anti*** conformation (Figure 2); the chlorine atoms do not engage in any close contacts or halogen bonding but rather are situated in a relatively interaction-free position in the crystal structure. While the opposite **Cl-*syn*** conformation seems to be possible, there is no structural disorder observed (with a ***syn-***/***anti-*** swap), as noted for the analogous **NomF** isomer.^50^ There are no conformational differences between the optimized and solid-state structures for the **NmpCl** isomer, while for the **NppCl** isomer, this formalism is not applicable on symmetry grounds with both *para*-ring substitutions.

### Contacts Analysis^68−74,90,91^

The intermolecular contact types on the Hirshfeld surface were analyzed in **NxxCl** using the MoProViewer software.^72^ The proportions of the main contacts in the nine **NxxCl** crystal structures are shown in Figure 13. Contacts between two chemical types (X,Y) are over-represented when their proportion *C*xy is larger than that obtained by probability products of the chemical contents *S*x and *S*y on the Hirshfeld surface.^72−74^ Enrichment ratios are therefore obtained by dividing the actual proportion by the equiprobable reference value. The most enriched contacts are the strong N–H···N and N–H···O=C hydrogen bonding interactions with average enrichment ratios <*E* > larger than 4 (Table 7; Supporting Information Table S5). The standard deviations of *E*_HnO_ and *E*_HnN_ are large because for many crystals one of these two *E* values is zero, as only one of such hydrogen bond types occurs. The three **NoxCl** isomers have an intramolecular N–H···N hydrogen bond (not counted in the Hirshfeld statistics) but are devoid of an intermolecular one (Figure 6). The **NmmCl**, **NmpCl**, and **NpmCl** isomers display an intermolecular N–H···N hydrogen bond, whereas in both **NmoCl** and **NpoCl** isomers, an N–H···O=C hydrogen bond is observed (Figure 3). As the **NxxCl** molecules have two strong hydrogen bond acceptors with a deficit of strong donors (only one N–H group is available), weak hydrogen bonds are also favored as _pyridine_N···H–C and C=O···H–C. The enrichment ratios of *E*_NHc_ and *E*_OHc_ are −87% *anti*-correlated in the eight anhydrous crystal structure packings.

**Table 7:** Average X···Y Contact Enrichment Ratios between the Different Chemical Types in the Eight Nonhydrated **NxxCl** Crystal Structures^a^

chem.	C	H_C_	Cl	N	H_N_	O
<surface> %	35.5	37.6	13.8	5.2	2.9	5.1
C	**1.2(5)**	1.0(4)	0.9(3)	1.0(6)	0.8(7)	0.6(3)
H_C_		0.7(3)	**1.6(3)**	**1.2(4)**	0.7(4)	**1.6(6)**
Cl			0.6(7)	0.1(2)	0.0(1)	0.4(4)
N				0.9(11)	**4.3(55)**	0.1(2)
H_N_					0.01(4)	**4.5(69)**
O						0.1(3)

a The sample standard deviations are given between parentheses. The over-represented contacts are highlighted in **bold** characters. The second line shows the average chemical content on the Hirshfeld surface. The hydrophobic atoms C, H_C_, and Cl have been regrouped in the table. H_C_ and H_N_ refer to hydrogen atoms bound to carbon or nitrogen that are distinguished as they are chemically very different.

Among contacts between the C, H_C,_ and Cl hydrophobic atoms, the weak Cl···H_C_ hydrogen bonds are enriched. The C···C stacking contacts are also significantly enriched,^32^ as would be expected for heterocycles.^69^ All of the nine crystal structures have Cl···H_C_ weak hydrogen bonds that are over-represented. This is easily understood as H_C_ is the chemical type that has the largest representation at 37% on the Hirshfeld surface and as organic halogen atoms are favored contact partners for H_C_.^90,91^

The **NxxCl** isomers have mostly hydrophobic atoms (C, H_C_, and Cl) at their Hirshfeld surface, with a proportion reaching 86%. The amount of purely hydrophobic contacts within these atoms is remarkably stable at 77 ± 1.3% for the eight nonhydrated **NxxCl** isomers, and this corresponds to a global hydrophobic contacts enrichment of 1.03. In contrast, the polar···polar contacts only represent 3% of the Hirshfeld surface but are globally over-represented with *E* = 1.63. The cross polar/hydrophobic contacts make a total of 20% of the surface, are moderately under-represented at *E* = 0.84, and are mainly due to weak C–H···O and C–H···N hydrogen bonds.^27,28^

The **NpoCl** and **NmmCl** crystals are characterized by limited aromatic ring stacking as the two rings of the molecules have very different orientations (Table 2).^32^ Conversely, these two compounds have high amounts of weak C–H···π hydrogen bonds (*E*_HcC_ = 1.56 and 1.42, respectively).^29^ In **NpoCl**, the two aromatic rings are nearly perpendicular (with C_6_/C_5_N = 83.24(7)° in Table 2), and this crystal packing consequently exhibits extensive C–H···π interactions.^29^ On the other hand, **NmpCl** and **NomCl** crystals show extensive aromatic ring stacking, and the two aromatic rings of each molecule are effectively parallel.^32^ In the **NomCl** packing, all the molecules are close to planarity and are essentially parallel [C_6_/C_5_N = 1.07(6)°], while in **NmpCl**, the aromatic rings have an orientation of C_6_/C_5_N = 7.65(14)°. Therefore, in summary, the C···C and C···H_C_ enrichment values are −96.8% *anti*-correlated in the eight anhydrous **NxxCl** structures. Similarly, for contact proportions *C*_XY_, the *anti*-correlation of enrichments reaches −90.4%.

In broad terms, chlorine···chlorine contacts are generally avoided (with <*E* > = 0.6) but with the exception of **NmpCl** and **NooCl**. In these two crystal structures, the Cl···Cl contacts do not correspond to halogen bonds (where the σ-hole faces the electronegative crown) but are merely at the van der Waals contact level and result from the translation of molecules along a short unit cell axis.

To find some hints why the **NxxF** series^50^ shows poor isomorphism with the **NxxCl** series, the contact enrichments of F and Cl atoms were compared in Table S6 (Supporting Information). One major difference is that the **NxxF** series showed an average enrichment of only 1.3 for the F···H_C_ weak hydrogen bonds when compared to 1.6 for the Cl···H_C_ intermolecular interactions in **NxxCl**. Of further note is that Feng and co-workers have shown by rotational spectroscopy that in a competition between weak H-bonds in the CH_2_FCl**·**H_2_C=O adduct, the C–H···Cl intermolecular interaction is preferred to C–H···F.^100^

From a charge density topology point of view, the strengths of H···Cl hydrogen bonds appear to be also more important than that of the H···F type.^101,102^ Indeed, a starting degree of covalence appears at longer distances for Cl than for F.^102^ Accordingly, for a given internuclear distance H···halogen (halogen = F, Cl), the electron density at the bond critical point of H···Cl is larger than at that of H···F because the penetration of electron shells is more important in the case of Cl.^101^ Hence, due to the higher electronegativity of F compared to Cl, the H···F interaction tends to be more closed-shell in nature and a significant shared-shell character can be only present at very short H···F geometries. In addition, within the natural bond orbital theory (NBO),^103^ it has been established that the charge transfer from the acceptor (halogen) toward the X-H σ* molecular orbital can be considered as the signature of the X-H···(halogen) hydrogen bond strength. Again, due to the larger electronegativity of F, the charge transfer in hydrogen bonds will be less important with F than with Cl, leading to weaker interactions with the former acceptor. The interaction propensity of fluorine is different from that of chlorine and bromine, and this might explain the lower isomorphism of **NxxF**(50) with the **NxxCl**^this work^ series compared to **NxxBr**.^54^

In conclusion, the nine **NxxCl** crystal structures fulfill the following contacts in order of priority: (*i*) one strong intra- or intermolecular hydrogen bond involving N–H with N_pyridine_ or O=C; (*ii*) the remaining hydrogen acceptor atom interacts with H_C_ atoms; (*iii*) weak C–Cl···H_C_ hydrogen bonds are always formed; (*iv*) hydrophobic interactions between the H_C_ and C atoms represent, on average, 50 ± 2% of the contact surface; and (*v*) aromatic ring stacking is favored when the two rings and their symmetry related partners have similar orientations,^32^ while weak C–H···π hydrogen bonding interactions occur mostly when the aromatic ring orientations differ significantly.^27,29^

In our previous studies with **Clxx**,^56^ we have noted the paucity of halogen bonding and notably Cl···Cl contacts in these amide-bridge reversed isomers (compared to **NxxCl**). This behavior is not too dissimilar to that observed for **NxxCl**. In related research, we have considered the competition between the F, O=C, N–H, and aromatic rings in terms of influencing interactions and aggregation.^36^ We have also speculated on the number of halogen atoms and type of halogen atom needed to tip the interactions from hydrogen bonding toward halogen bonding of the type C–Cl···O=C, C–Cl···N_pyridine_, and C–Cl···Cl–C. Indeed, research studies on the competition between interactions in crystal structure formation have been pursued with much interest recently in structural systematic studies of extensive series of molecules and in co-crystal formation.^104−109^ It has been noted that detailed studies are still rare.^109^ However, the ongoing structural systematic reports of series of closely related compounds together with both computational and database analyses should enable more in-depth analyses and predictive abilities in the near future.^49,56,109,110^

In research concerning the competition between hydrogen-bonding and halogen-bonding interactions in the crystal structures of pentachlorophenol (C_6_Cl_5_OH) and pentabromophenol (C_6_Br_5_OH), it has been pointed out that(*C*)O–H···O(H)–C is stronger than solitary C–Cl···Cl–C and C–Br···Br–C interactions, as observed from the topological properties of ρ(**r**) at the corresponding bond critical points (H···O > Br···Br > Cl···Cl).^111^ Similar conclusions were also raised with the electrophilic–nucleophilic interactions between the corresponding local charge concentration (CC) and charge depletion (CD) sites in the valence shell of atoms involved in the intermolecular interactions (H···O > Br···Br > Cl···Cl), here characterized by the topology of *L*(**r**) = −∇^2^ρ(**r**). In both crystal structures, neither O–H···Cl–C nor O–H···Br–C intermolecular hydrogen bonds are observed, indicating that O is a better acceptor in O–H···O(H)–C hydrogen bonds than Cl and Br in the former. On the other hand, halogen bonding of the C–Cl···O(H)–C and C–Br···O(H)–C type is not observed because involving O as an acceptor in (*C*)O–H···O(H)–C hydrogen bonds leads to stronger interactions. Consequently, if halogen bonding of the type C–Hal···O=C, C–Hal···N_pyridine_, or C–Hal···Hal-C should compete with H···O=C, H···N_pyridine_, and H···Hal-C hydrogen bonds, the best candidates should be found with the heavier halogens (Hal = Br, I); otherwise, the number of acceptors should be larger to permit Hal atoms to take the place of donors once the best donors have been used up.^109,111^ This is what recent structural research is beginning to show.^49,109^

## Summary and Conclusions

The 3 × 3 isomer grid of **NxxCl** [*N*-chlorophenyl(pyridine)carboxamides] structures displays correlations with their **NxxX** (**X** = F, Br or M = Me) analogues. This is readily demonstrated with five isomorphous relationships between pairs of **NxxCl** and **NxxBr** structures.^54,84,86^ The **NxxCl** general behavior mimics the amide-bridge reversed **Clxx** series^56^ in its relationships with both methyl and bromo-substituted derivatives^53,54^ but not with the fluorine analogues.^50,52^ As such, there is a transition along the Me →F → Cl → Br series of structures where the increasing influence of the halogen atom is noted especially from F to Br. The impact on the structure and the increased structural overlap (isomorphous behavior) between the Cl and Br derivatives are noted here for **NxxCl** and in **NxxBr**.^54^ The matching of molecular crystal structures on the CSD readily demonstrates the value of systematic studies to the structural science community and the (bio)pharmaceutical sector in particular.^49,110^

N–H···N interactions dominate in comparison to N–H···O=C in the **NxxCl** series. This has been noted over several structural series between molecules where there is direct competition between O=C and N_pyridine_ as acceptors of the N–H amide hydrogen bond donor group.^50−54,56^ The remaining O or N acceptor atom usually interacts with aromatic C–H groups. Weak C–H···Cl interactions are often present in the **NxxCl** structures but not in any predictable way. The planar **NooCl** structure is peculiar with its intramolecular Cl···H_N_···N_pyridine_ synergistic combination. Aromatic ring interactions arise especially where symmetry favors stacking,^32^ and C–H···π interactions occur often where the aromatic plane orientations differ significantly.^29^ In models, the optimized geometries of the **NxxCl** isomers mostly resemble the geometries of related isomer grids, *i.e.,***NxxF** and **NxxM**.^50,51^ They also mostly correspond with their crystal structures, and differences arise if there is a favorable interaction in the crystal structure that necessitates a change in **NxxCl** geometry. In doing so, the divergence between the models and solid-state geometry is more than compensated for in crystal packing forces and the resulting favorable lattice energy. At the solid/liquid boundary, the melting point of a member of the **NxxCl** series follows Carnelley’s rule on molecular symmetry but with distinct differences (typically lower average melting points) than noted for their **Clxx** analogues.^56^

The 18-member series of **Brxx**/**NxxBr** structures is in preparation for publication with additional contact analysis and for comparisons with **NxxX** (**X** = F,^50^ Cl^this work^ or M = Me^51^) analogues together with their corresponding amide-bridge reversed isomers (**Mxx**,^53^**Fxx**,^52^**Clxx**^56^). The increasing role and influence of the heavier halogen in the crystal structures will be assessed in terms of the competition between hydrogen and halogen bonding interactions.^109^ Investigations on the physicochemical properties and trends of series of isomers of 72+ molecules (including polymorphs) will be available for future computational analysis.

## Supporting Information Available

Crystallography and melting point data (Section I); enlarged diagrams from Figure 12 (main paper) (Section II); ATR-IR and NMR data for the nine **NxxCl** isomers (**NppCl·H2O** to **NooCl**) (Section III); melting points and electrostatic energy diagrams (for ESI) (Section IV); and contact enrichment studies for **NxxCl** (Section V) (PDF)

## Accession Codes

CCDC 2074347–2074355 contain the supplementary crystallographic data for this paper. These data can be obtained free of charge via www.ccdc.cam.ac.uk/data_request/cif, or by emailing data_request@ccdc.cam.ac.uk, or by contacting The Cambridge Crystallographic Data Centre, 12 Union Road, Cambridge CB2 1EZ, UK; fax: +44 1223 336033.
