# Supplementary material for: Probing the Electronic Properties and Interaction Landscapes in a Series of N-(Chlorophenyl)pyridinecarboxamides
Source: Cryst Growth Des. 2022 Apr 13;22(5):3343–58. doi: 10.1021/acs.cgd.2c00153 (PMC9074230; doi:10.1021/acs.cgd.2c00153)
Supplement: Supplementary file 1 — cg2c00153_si_001.pdf [file cg2c00153_si_001.pdf]

ELECTRONIC SUPPLEMENTARY INFORMATION

PROBING THE ELECTRONIC PROPERTIES AND INTERACTION LANDSCAPES IN A SERIES OF *N*-(CHLOROPHENYL)PYRIDINECARBOXAMIDES

John F. Gallagher,* Niall Hehir, Pavle Mocilac, Chloé Violin, Brendan O'Connor#

School of Chemical Sciences, Dublin City University, Dublin 9, Ireland

School of Biotechnology, Dublin City University, Dublin 9, Ireland.#

and

Emmanuel Aubert, Enrique Espinosa, Benoît Guillot, Christian Jelsch

CRM[2], CNRS UMR 7036, Faculté des Sciences et Technologies, Université de Lorraine, BP 70239, Boulevard des Aiguillettes, 54506 Vandoeuvre-lès-Nancy, France

Corresponding Author:*
Professor John F. Gallagher,
School of Chemical Sciences,
Dublin City University, Dublin 9, Ireland
e-mail: john.gallagher@dcu.ie

ESI comprises five sections (labelled Sections I to V).

**Section I**	Crystallography and melting point data.
**Section II**	Enlarged diagrams from **Figure 12** (main paper).
**Section III**	ATR-IR and NMR data for the nine **NxxCl** isomers (**NppCl•H_2O** to **NooCl**).
**Section IV**	Melting points and electrostatic energy diagrams (for ESI).
**Section V**	Contact enrichment studies for **NxxCl**.

TABLE OF CONTENTS

Section I Crystallography and melting point data.

Page No.

2. Table of contents and Schematic diagram of the **NxxCl** reaction products.
3. Table S1 Crystallographic Experimental details.
5. Table S2 Selected intra- and intermolecular hydrogen-bond parameters (Å, °).
6. Table S3a,b Comparisons of the **NxxCl** and **NxxBr** crystal structures (isomer grids).
7. Table S4a Melting points (°C) of the **NxxCl** and **Clxx** isomer grids.
Table S4b Isomorphous relationships for **NxxCl** and **NxxBr**.

Section II Enlarged diagrams from **Figure 12** (main paper).

8. **Figure 12** from the main paper (showing conformational analysis and trends).
9. Nine **NxxCl** PES diagrams as depicted in the main paper.
10-12 **NppCl** to **NpoCl** (three enlarged **NpxCl** versions for clarity).
13-15 **NmpCl** to **NmoCl** (three enlarged **NmxCl** Figures).
16-18 **NopCl** to **NooCl** (three enlarged **NoxCl** Figures).

Section III ATR-IR and NMR data for nine **NxxCl** isomers (**NppCl** to **NooCl**).

19-54 ATR-IR spectra, ^{1}H-NMR in $CDCl_3$, ^{1}H-NMR in DMSO-d_6, ^{13}C-NMR in DMSO-d_6.
55. ATR-IR spectra (a composite diagram of the nine **NxxCl** ATR-IR spectra).

Section IV Melting points and electrostatic energy diagrams (for ESI).

56-58 **Figures S01** to **Figures S06**.

Section V Contact enrichment studies for **NxxCl**.

59-60

Table S5: The contact enrichment ratios for the eight anhydrous **NxxCl** crystal structures. Outlier numbers highlighted in **bold** are discussed.

Table S6: Enrichment of contacts with chlorine in the **NxxCl** and with fluorine in the **NxxF** isomer series.

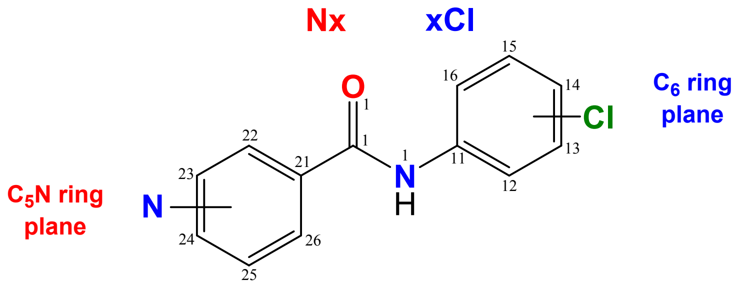

Schematic diagram of the NxxCl reaction products

Table S1: Crystallographic Experimental details

Experiments were carried out at 294(1) K with Mo *Kα* radiation. H atoms were treated by a mixture of independent (N-H) and constrained (C-H) refinement.

	NppCl•H_2O	NpmCl	NpoCl	NmpCl
**Crystal data**				
Chemical formula	$C_{12}H_{11}ClN_2O_2$	$C_{12}H_9ClN_2O$	$C_{12}H_9ClN_2O$	$C_{12}H_9ClN_2O$
M_r (g.mol^{-1})	250.68	232.66	232.66	232.66
Crystal system, space group	Orthorhombic, *Pbca*	Monoclinic, $P2_1/n$	Monoclinic, *Cc*	Monoclinic, $P2_1$
a, *b*, *c* (Å)	7.4118(2), 11.8244(2), 26.7559(2)	8.1442(2), 10.3052(2), 13.3843(3)	12.6952(6), 10.5329(3), 9.6681(5)	9.9826(3), 3.8840(1), 13.7564(5)
α, β, γ (°)	90, 90, 90	90, 103.893(2), 90	90, 121.007(7), 90	90, 95.088(3), 90
V (Å^3), *Z*	2344.89(8), 8	1090.45(4), 4	1108.06(11), 4	531.27(3), 2
μ (mm^{-1})	0.32	0.33	0.32	0.34
Crystal size (mm)	0.43×0.31×0.08	0.55×0.35×0.25	0.44×0.25×0.17	0.39×0.16×0.15
**Data collection**				
Measured, independent, observed [$I > 2\sigma(I)$] reflections	16045, 2524, 1739	7512, 2411, 1891	3884, 1601, 1491	3626, 1736, 1572
R_{int}	0.039	0.020	0.019	0.021
$(\sin \theta/\lambda)_{max}$ (Å^{-1})	0.641	0.657	0.653	0.648
**Refinement**				
$R[F^2>2\sigma(F^2)]$, $wR(F^2)$, S	0.042, 0.108, 1.03	0.035, 0.102, 1.07	0.026, 0.067, 1.10	0.043, 0.114, 1.03
No. of reflections	2524	2411	1601	1736
No. of parameters	166	150	149	149
No. of restraints	0	0	2	1
$\Delta\rho_{max}$, $\Delta\rho_{min}$ (e.Å^{-3})	0.32, -0.20	0.20, -0.29	0.18, -0.14	0.29, -0.23
Absolute structure and parameter	–	–	Flack x from 332 quotients [(I+)-(I-)]/[(I+)+(I-)], 0.03(4)	Classical Flack method, -0.04 (11)

	**NmmCl**	**NmoCl**	**NopCl**	**NomCl**	**NooCl**
**Crystal data**					
Chemical formula	$C_{12}H_9ClN_2O$	$C_{12}H_9ClN_2O$	$C_{12}H_9ClN_2O$	$C_{12}H_9ClN_2O$	$C_{12}H_9ClN_2O$
M_r (g.mol^{-1})	232.66	232.66	232.66	232.66	232.66
Crystal system, space group	Monoclinic, $P2_1/n$	Monoclinic, $P2_1/c$	Triclinic, $P\bar{1}$	Triclinic, $P\bar{1}$	Orthorhombic, *Pbca*
a, *b*, *c* (Å)	9.1339(2), 12.2820(2), 9.9477(2)	17.4001(8), 4.9503(2), 12.9596(7)	6.2976(2), 8.2399(3), 10.7563(3)	6.0819(3), 7.4928(4), 12.2122(6)	4.6511(14), 14.234(4), 33.626(11)
α, β, γ (°)	90, 106.779(2), 90	90, 104.873(5), 90	87.454(3), 86.485(3), 77.548(3)	95.948(4), 101.771(4), 99.435(4)	90, 90, 90
V (Å^3), Z	1068.45 (4),4	1078.89(9), 4	543.73(3), 2	532.00(5), 2	2226.2(11), 8
μ (mm^{-1})	0.33	0.33	0.33	0.34	0.32
Crystal size (mm)	0.60 × 0.58 × 0.34	0.33 × 0.13 × 0.05	0.58 × 0.42 × 0.13	0.55 × 0.34 × 0.19	0.71 × 0.11 × 0.05
**Data collection**					
Measured, independent, observed [$I > 2\sigma(I)$] reflections	11679, 3420, 2771	7364, 2359, 1537	9126, 2871, 2161	4556, 2758, 2151	13048, 2347, 908
R_{int}	0.016	0.055	0.015	0.012	0.082
$(\sin \theta/\lambda)_{max}$ (Å^{-1})	0.736	0.647	0.694	0.695	0.638
**Refinement**					
$R[F^2>2\sigma(F^2)]$, $wR(F^2)$, S	0.038, 0.115, 1.11	0.066, 0.149, 1.08	0.038, 0.105, 1.05	0.038, 0.124, 1.08	0.051, 0.120, 0.89
No. of reflections	3420	2359	2871	2758	2347
No. of parameters	149	149	149	149	149
No. of restraints	0	0	0	0	0
$\Delta\rho_{max}$, $\Delta\rho_{min}$ (e Å^{-3})	0.31, -0.29	0.31, -0.17	0.25, -0.28	0.26, -0.23	0.14, -0.11

Computer programs: *SHELXL2014*/7 (Sheldrick, 2014).
Analysis of absolute structure: Parsons, Flack and Wagner, *Acta Cryst*. **B69** (2013), 249-259.

Cambridge Structural Database analysis on closely related structures.[49]
PACLEO = *N*-(5-chloropyridin-2-yl)benzamide in triclinic space group $P\bar{1}$ with *Z'*=4.[109]
PELKUN = *N*-phenyl-2-chloronicotinamide in orthorhombic space group *Pccn* with *Z'*=1.[79]

GEPQIC[85] = **NopCl** (as published in reference 85).
NmoCl (in $P2_1/c$) is isomorphous with **NmoBr** (**TICDOZ01**)[84] (in $P2_1/a$), whereas **NmoM**[51] and a polymorph of **NmoBr** (**TICDOZ**)[86] are effectively isomorphous in space group *Pbca***.**

Table S2: **Selected intra- and intermolecular hydrogen-bond parameters (Å, °)**

D—H···*A*	*D*—H (Å)	H···*A* (Å)	*D*···*A* (Å)	*D*—H···*A* (°)
**NppCl•H_2O**				
N1—H1···O1W	0.85(2)	1.99(2)	2.831(2)	173.1(19)
C16—H16···O1	0.93	2.25	2.856(2)	122
O1W—H1W···O1^{i}	0.83(3)	2.03(3)	2.838(3)	164(2)
O1W—H2W···N24^{ii}	0.88(3)	2.04(3)	2.903(2)	166(2)
**NpmCl**				
N1—H1···N24^{iii}	0.843(17)	2.322(18)	3.1373(17)	162.9(16)
C12—H12···O1	0.93	2.24	2.8038(17)	118
C22—H22···N24^{iii}	0.93	2.59	3.4225(18)	149
C23—H23···O1^{iv}	0.93	2.40	3.1426(19)	137
**NpoCl**				
N1—H1···O1^{v}	0.72(2)	2.11(2)	2.797(2)	159(3)
N1—H1···C26	0.72(2)	2.64(3)	2.906(3)	105(2)
**NmpCl**				
N1—H1···N23^{vi}	0.90(3)	2.18(3)	3.079(3)	170(3)
C12—H12···O1	0.93	2.43	2.898(4)	111
C26—H26···O1^{vii}	0.93	2.54	3.226(3)	131
**NmmCl**				
N1—H1···N23^{viii}	0.848(16)	2.275(17)	3.0842(13)	159.7(15)
C12—H12···O1	0.93	2.38	2.8897(14)	114.1
C14—H14···O1^{ix}	0.93	2.46	3.3129(14)	153.3
**NmoCl**				
N1—H1···O1^{x}	0.85(2)	2.08(3)	2.884(3)	158(2)
C16—H16···O1	0.93	2.53	2.909(3)	105
**NopCl_[GEPQIC]**				
N1—H1···N22	0.838(16)	2.217(16)	2.6631(16)	113.4 (13)
C12—H12···O1	0.93	2.29	2.8954(17)	122
C16—H16···O1^{xi}	0.93	2.46	3.2728(17)	147
**NomCl**				
N1—H1···N22	0.822(17)	2.187(17)	2.6536(13)	116.1(15)
C12—H12···O1	0.93	2.29	2.8764(15)	120.6
C16—H16···O1^{xii}	0.93	2.54	3.2341(17)	131.4
**NooCl**				
N1—H1···N22	0.84(3)	2.09(3)	2.624 (4)	121(2)
N1—H1···Cl22	0.84(3)	2.54(3)	2.937 (3)	110(2)
C16—H16···O1	0.93	2.34	2.929 (4)	121

Symmetry code(s):
(i) -*x*+1/2, *y*+1/2, *z*; (ii) -*x*, -*y*+2, -*z*+1; (iii) -*x*+1/2, *y*-1/2, -*z*+3/2; (iv) *x*+1/2, -*y*+1/2, *z*+1/2; (v) *x*, -*y*+1, *z*-1/2; (vi) -*x*, *y*-1/2, -*z*+1; (vii) -*x*+1, *y*-1/2, -*z*+1; (viii) *x*-1/2, -*y*+3/2, *z*-1/2; (ix) *x*-1/2, -*y*+1/2, *z*-1/2; (x) *x*, *y*-1, *z*; (xi) *x*+1, *y*, *z*; (xii) *x*-1, *y*, *z*.

Table S3a,b: Comparisons of **NxxCl**[this work] and **NxxBr**[54,84] crystal structures (isomer grids).

Table S3a: The 3 × 3 **NxxCl** isomer grid

NxxCl	Np	Nm	No
pCl	**NppCl•H_2O**<br>Mixed due to hydrate<br>Space group = *Pbca*	**NmpCl**<br>N-H…N<br>Space group = $P2_1$	**NopCl**<br>N-H…$N_{intramolecular}$<br>***P*-1** [**GEPQIC**][85]
mCl	**NpmCl**<br>N-H…N<br>Space group = $\boldsymbol{P2_1/n}$	**NmmCl**<br>N-H…N<br>Space group = $P2_1/n$	**NomCl**<br>N-H…$N_{intramolecular}$<br>Space group = *P*-1
oCl	**NpoCl**<br>**N-H…O=C**<br>Space group = ***Cc***	**NmoCl**<br>**N-H…O=C**<br>Space group = $\boldsymbol{P2_1/c}$	**NooCl**<br>N-H…$N_{intramolecular}$<br>Space group = ***Pbca***

The **NxxCl** structures typically have weak contacts involving the Cl atoms apart from the intramolecular **NoxCl** (especially **NooCl**). Isomorphous NxxCl/NxxBr structures are in green for comparison with **Table 3b**.

Table S3b: The 3 × 3 **NxxBr**[54] isomer grid

NxxBr	Np	Nm	No
pBr	**NppBr**<br>**N-H…O=C**/N…Br<br>Space group = $P2_1$	**NmpBr**<br>N-H…O=C<br>Space group = *C*2/*c*	**NopBr**<br>N-H…$N_{intramolecular}$<br>Space group = ***P*-1**
mBr	**NpmBr**<br>N-H...N<br>Space group = $\boldsymbol{P2_1/n}$	**NmmBr**<br>**N-H…O=C**/N-H...N<br>Space group = *P*-1	**NomBr**<br>Stacking/N…Br<br>Space group = *C*2/*c*
oBr	**NpoBr**<br>**N-H…O=C**<br>Space group = ***Cc***	**NmoBr** two polymorphs **TICDOZ(01)**<br>**N-H…O=C**<br>Space group = $\boldsymbol{P2_1/a}$.[84]<br>Space group = *Pbca* for **TICDOZ**[86]	**NooBr**<br>N-H…$N_{intramolecular}$<br>Space group = ***Pbca***

Of note is that **NmoCl**[this work] ($P2_1/c$) is isomorphous with **NmoBr** (**TICDOZ01**)[84] (in space group $P2_1/a$, whereas **NmoM**[51] and a polymorph of **NmoBr** (**TICDOZ**)[86] are isomorphous in space group *Pbca*.

Table S4a: Melting points (°C) of the **NxxCl** and **Clxx** isomer grids.

	NpxCl	NmxCl	NoxCl
**NxpCl**	**139.0-140.0**	168.3-172.9	138.0-140.0
**NxmCl**	164.0-166.0	139.2-141.6	90.8-92.1
**NxoCl**	132.7-134.7[87]	**80.0-81.9**[87]	110.1-111.5
	**Clxp**	**Clxm**	**Clxo**
**Clpx**	206.2-208.4	150.1-151.3	131.5-134.5
**Clmx**	185.4-187.2	112.4-113.9	95.0–105.0
**Clox**	167.9-169.9	134.5-137.8	134.4-138.0

Reference 87 (of main manuscript) literature value for the **NpoCl** melting point = 134°C and **NmoCl** = 82°C.

Table S4b: Isomorphous relationships for **NxxCl**, **NxxBr**: comparisons with **Clxx**[56]/**Brxx**.[54]

NxxCl and NxxBr isomer grids			Clxx and Brxx isomer grids		
**NxxCl**	**Space group**	**NxxBr**[54]	**Clxx**[56]	**Space group**	**Brxx**[54]
NppCl•H_2O	Pbca ≠ $P2_1$	NppBr	**Clpp***	***$P2_1/c$***	**Brpp***
**NpmCl**	***$P2_1/n$***	**NpmBr**	Clmp (*Z′*=4)	P-1 ≠ P-1	Brmp (*Z′*=2)
**NpoCl**	***Cc***	**NpoBr**	**Clop**	***Pbca***	**Brop**
NmpCl	$P2_1$ ≠ C2/*c*	NmpBr	Clpm	P-1 ≠ C2/*c*	Brpm
NmmCl	$P2_1/n$ ≠ P-1	NmmBr	**Clmm•H_2O**	***$P2_1/c$***	**Brmm•H_2O**
**NmoCl**	***$P2_1/c$ – $P2_1/a$***	**NmoBr**[84]	**Clom**	***C2/c***	**Brom**
**NopCl**[85]	***P-1***	**NopBr**	Clpo	C2/c ≠ P-1	Brpo
NomCl	P-1 ≠ C2/c	NomBr	Clmo	P-1 ≠ $P2_1/c$	Brmo
**NooCl**	***Pbca***	**NooBr**	**Cloo**	***C2/c***	**Broo**

Footnote: Isomorphous pairs are highlighted in **bold** together with their ***common space group in italics***.
The ≠ symbol is for crystal structures that are not isomorphous; for **Clmp** and **Brmp**, *Z′* is also noted.
Clpp and **Brpp** are part of a larger isomorphous series as discussed previously.[54]

Of note are the isostructural **Clmp** (*Z*'=4) and **Brmp** (*Z*'=2) in *P*-1 (No. 2) and with two sets of similar unit cell axes **Clmp** [*a* = 11.4395(2), *b* = 12.9293(2) Å] and **Brmp** [*b* = 11.5019(5), *c* = 12.9305(6) Å] and with the third axis [*a* = 7.6612(3) Å] is halved in **Brmp** when compared with **Clmp** [*b* = 14.8943(4) Å].

A Cambridge Structural Database (CSD) study by Mukherjee and Desiraju[18,49] demonstrates a significant degree of similarity between pairs of structures with C-**X** bonds (**X** = Cl or Br).[18] In 2017, Giangreco and co-workers at the Cambridge Crystallographic Data centre (CCDC) have reported an extensive study matching molecular crystal structures in a systematic exploration of isostructurality.[110] Given the increasing numbers of structures being added to the CSD on a daily basis the value of such studies is of importance to the structural science community and especially the (bio)pharmaceutical sector.

Section II

An enlarged version of **Figure 12** showing conformational analysis and trends.
The individual diagrams are enlarged in the ESI (for ease of viewing of the finer details).

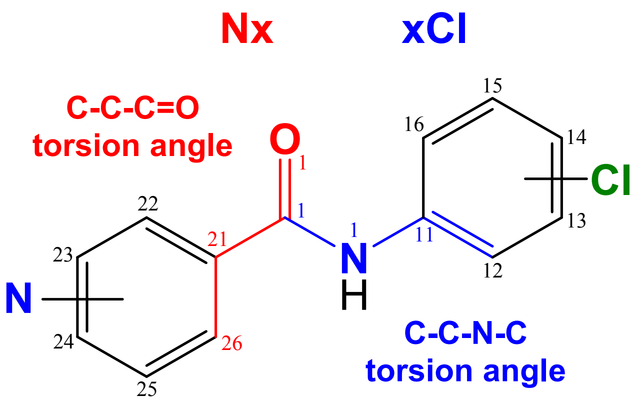

Scheme
Schematic diagram of the **NxxCl** general structure type showing the two torsion angles (**C26-C21-C1=O1** and **C12-C11-N1-C1**) for Potential Energy Scans and geometric analysis.

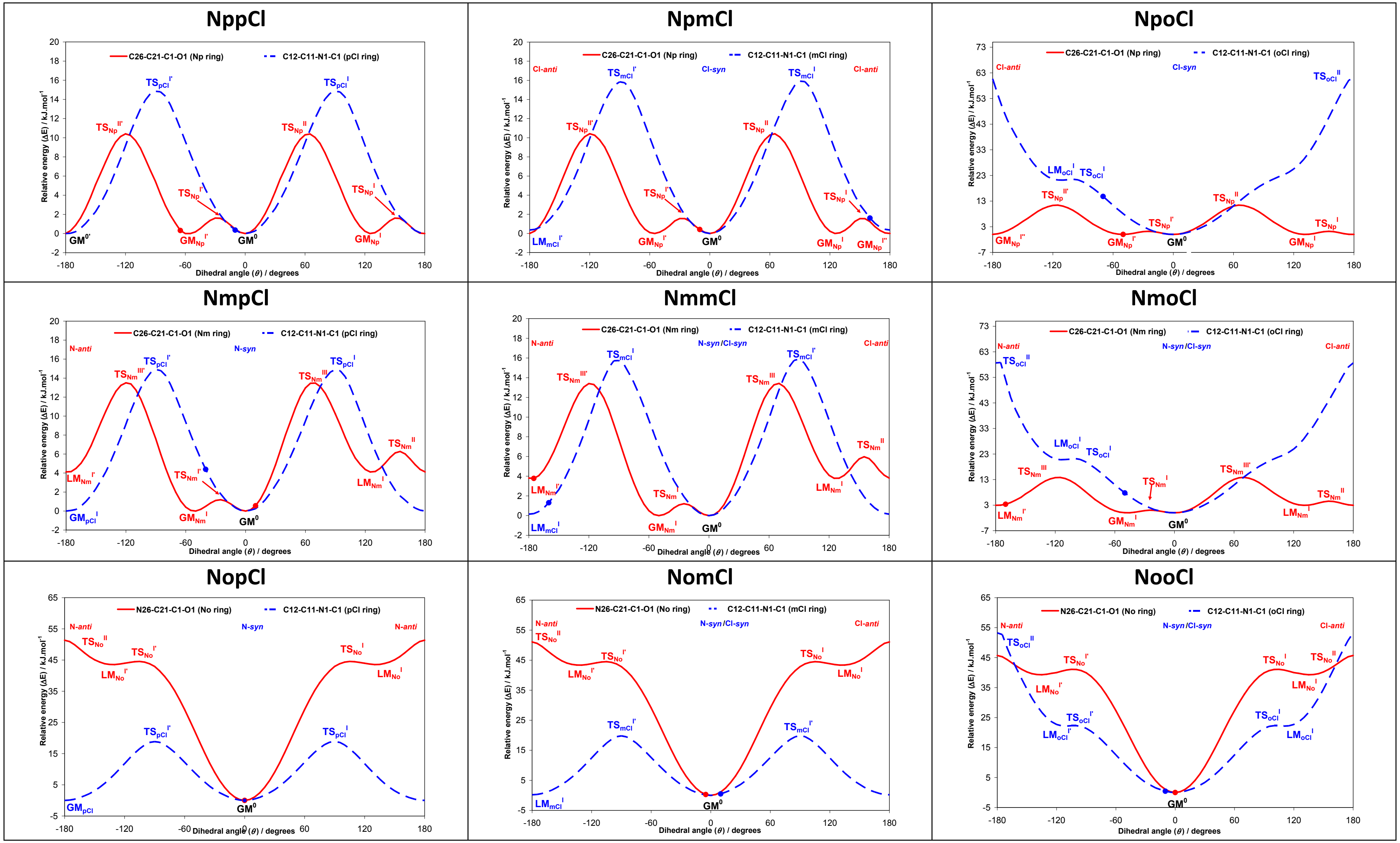
NppCl
C26-C21-C1-O1 (Np ring)
C12-C11-N1-C1 (pCl ring)
NpmCl
C26-C21-C1-O1 (Np ring)
C12-C11-N1-C1 (mCl ring)
Cl-anti
Cl-syn
NpoCl
C26-C21-C1-O1 (Np ring)
C12-C11-N1-C1 (oCl ring)
NmpCl
C26-C21-C1-O1 (Nm ring)
C12-C11-N1-C1 (pCl ring)
N-anti
N-syn
NmmCl
C26-C21-C1-O1 (Nm ring)
C12-C11-N1-C1 (mCl ring)
N-syn/Cl-syn
NmoCl
C26-C21-C1-O1 (Nm ring)
C12-C11-N1-C1 (oCl ring)
NopCl
N26-C21-C1-O1 (No ring)
C12-C11-N1-C1 (pCl ring)
NomCl
N26-C21-C1-O1 (No ring)
C12-C11-N1-C1 (mCl ring)
NooCl
N26-C21-C1-O1 (No ring)
C12-C11-N1-C1 (oCl ring)
Relative energy (ΔE) / kJ.mol-1
Dihedral angle (θ) / degrees

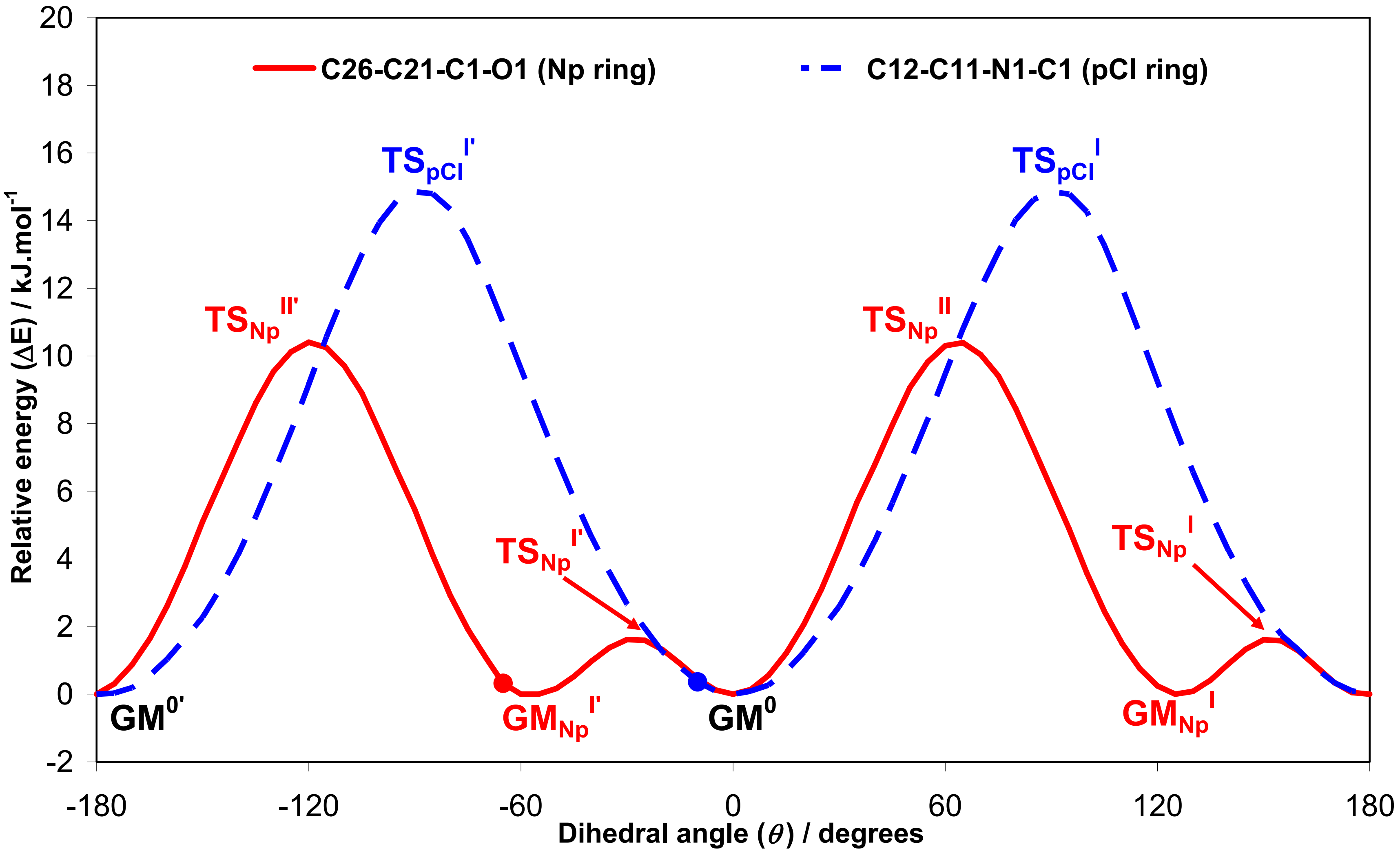

NppCl – Enlarged diagrams from the main paper (PES diagram) as Figure 12.

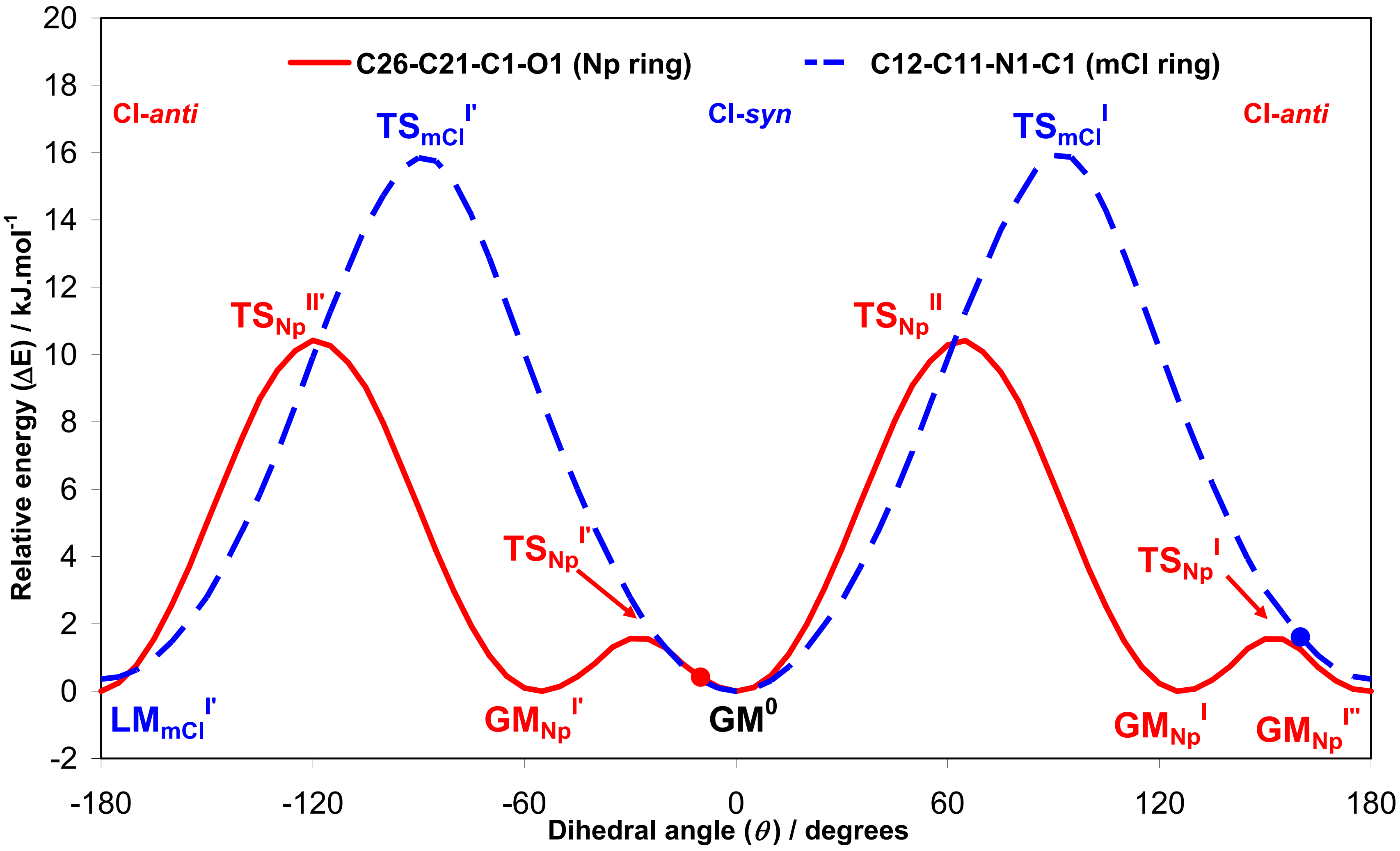
C26-C21-C1-O1 (Np ring)
C12-C11-N1-C1 (mCl ring)
Cl-anti
Cl-syn
Cl-anti
TSmCl I'
TSmCl I
TSNp II'
TSNp II
TSNp I'
TSNp I
LMmCl I'
GMNp I'
GM0
GMNp I
GMNp I"
Relative energy (ΔE) / kJ.mol-1
Dihedral angle (θ) / degrees
20
18
16
14
12
10
8
6
4
2
0
-2
-180
-120
-60
0
60
120
180

NpmCl

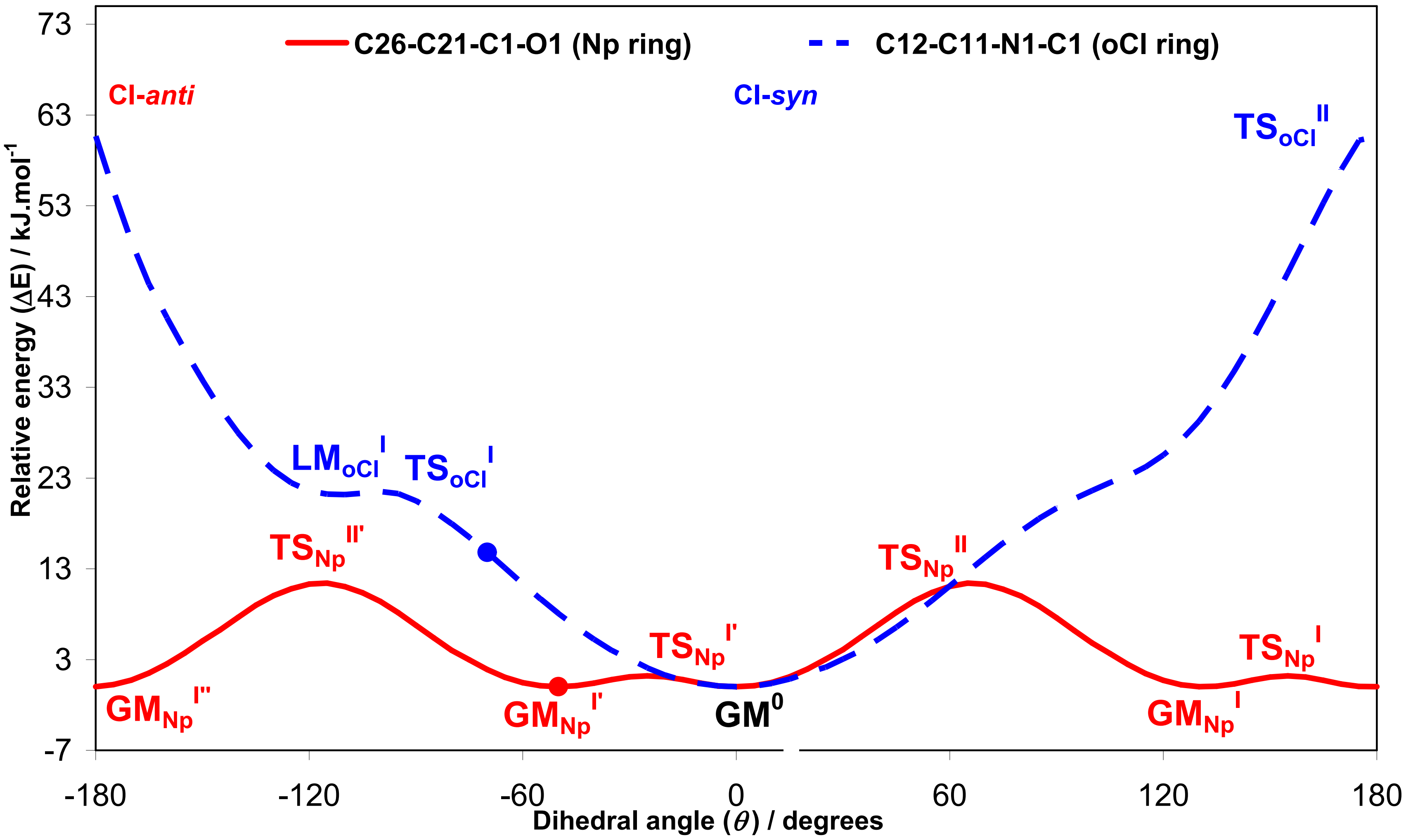

C26-C21-C1-O1 (Np ring)
C12-C11-N1-C1 (oCl ring)
Cl-anti
Cl-syn
TSoCl II
LMoCl I
TSoCl I
TSNp II'
TSNp II
TSNp I'
TSNp I
GMNp I''
GMNp I'
GM0
GMNp I
Relative energy (ΔE) / kJ.mol-1
73
63
53
43
33
23
13
3
-7
-180
-120
-60
0
60
120
180
Dihedral angle (θ) / degrees

NpoCl

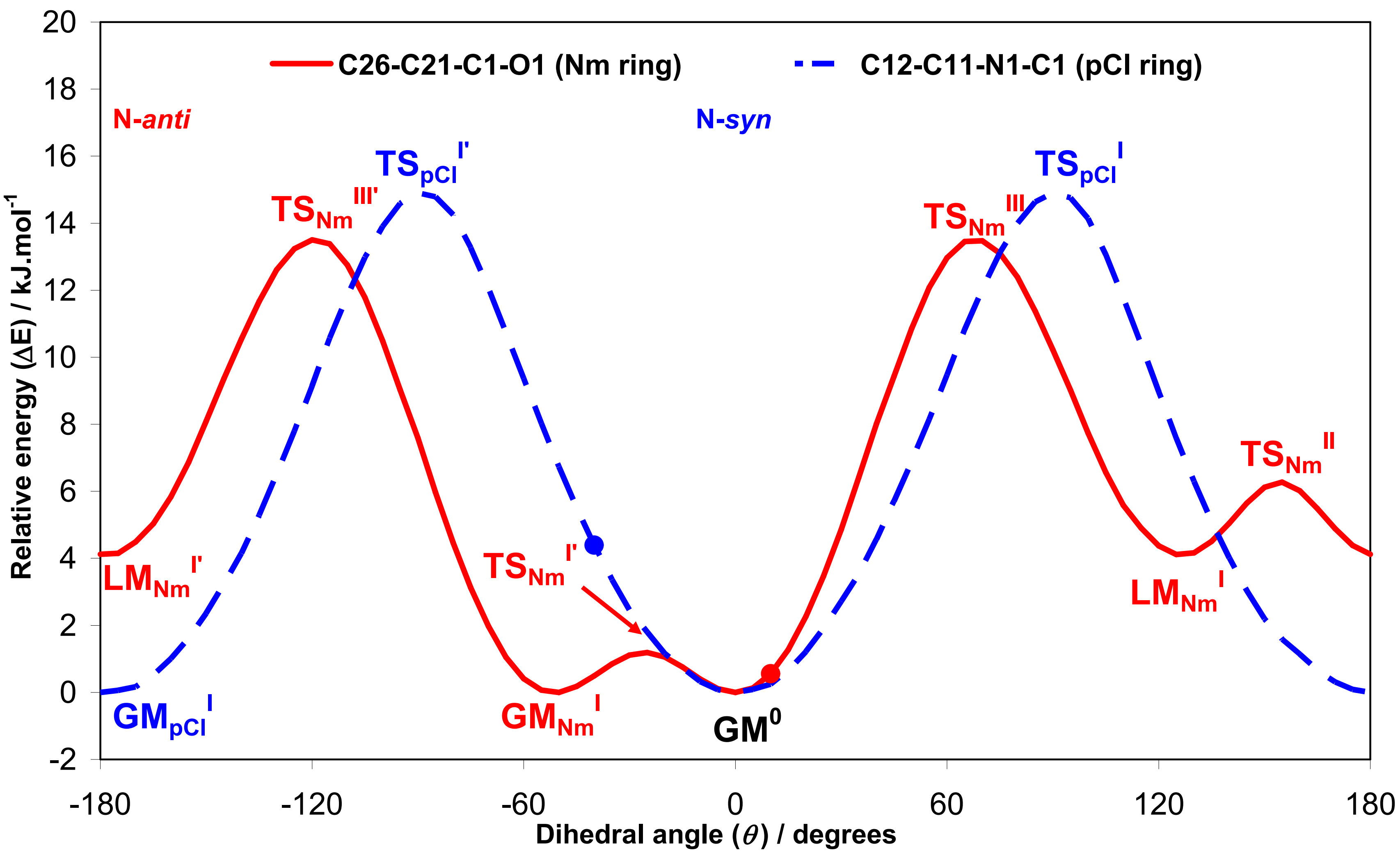
C26-C21-C1-O1 (Nm ring)
C12-C11-N1-C1 (pCl ring)
N-anti
N-syn
TSpCl I'
TSNm III'
TSpCl I
TSNm III
TSNm II
TSNm I'
LMNm I'
LMNm I
GMNm I
GMpCl I
GM0
Relative energy (ΔE) / kJ.mol-1
Dihedral angle (θ) / degrees
20
18
16
14
12
10
8
6
4
2
0
-2
-180
-120
-60
0
60
120
180

NmpCl

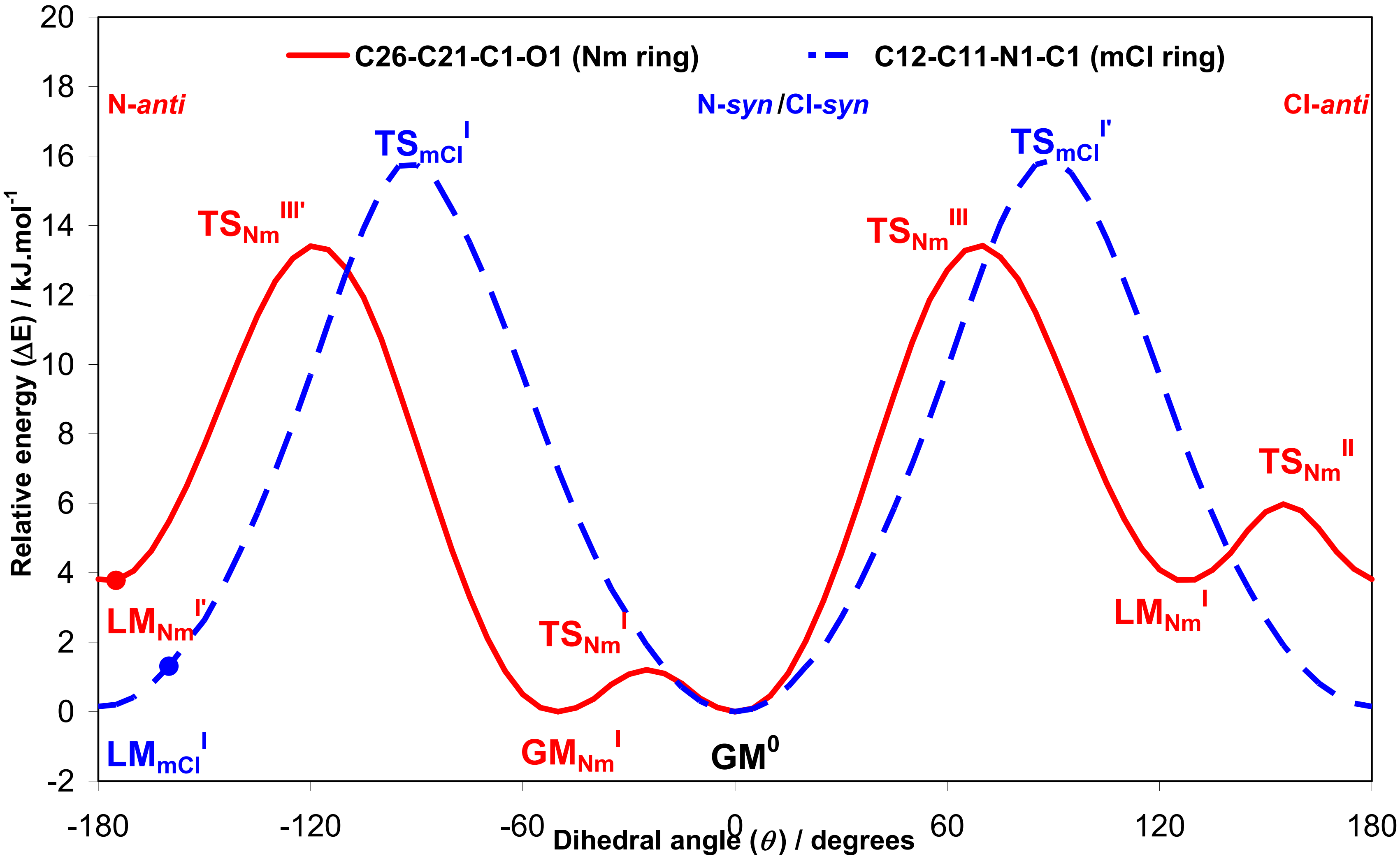

C26-C21-C1-O1 (Nm ring)
C12-C11-N1-C1 (mCl ring)
N-anti
N-syn /Cl-syn
Cl-anti
TSmCl I
TSmCl I'
TSNm III'
TSNm III
TSNm II
TSNm I
LMNm I'
LMNm I
LMmCl I
GMNm I
GM0
Relative energy (ΔE) / kJ.mol-1
Dihedral angle (θ) / degrees
20
18
16
14
12
10
8
6
4
2
0
-2
-180
-120
-60
0
60
120
180

NmmCl

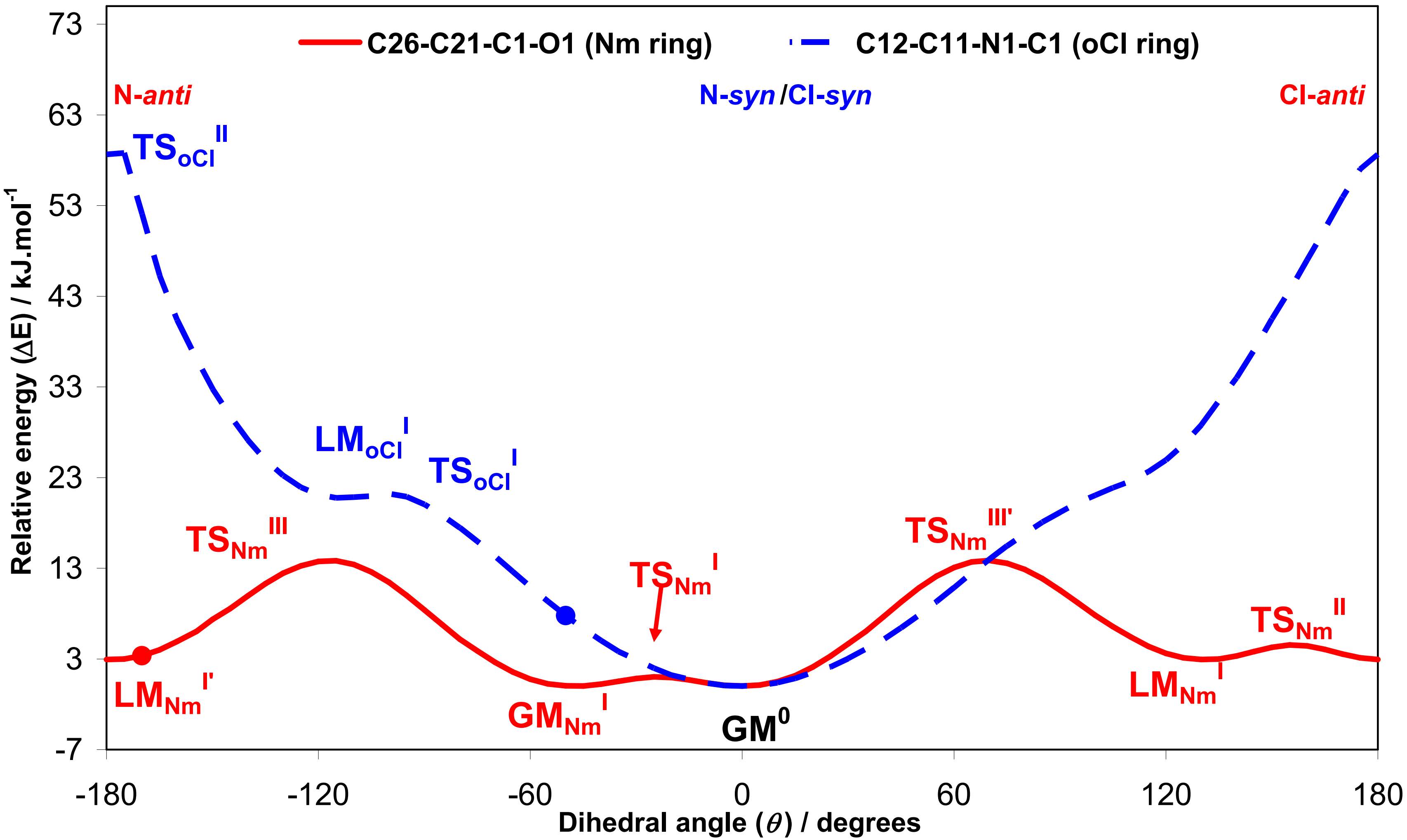
C26-C21-C1-O1 (Nm ring)
C12-C11-N1-C1 (oCl ring)
N-anti
N-syn/Cl-syn
Cl-anti
TSoCl II
LMoCl I
TSoCl I
TSNm III
TSNm I
TSNm III'
TSNm II
LMNm I'
GMNm I
GM0
LMNm I
Relative energy (ΔE) / kJ.mol-1
Dihedral angle (θ) / degrees
73
63
53
43
33
23
13
3
-7
-180
-120
-60
0
60
120
180

NmoCl

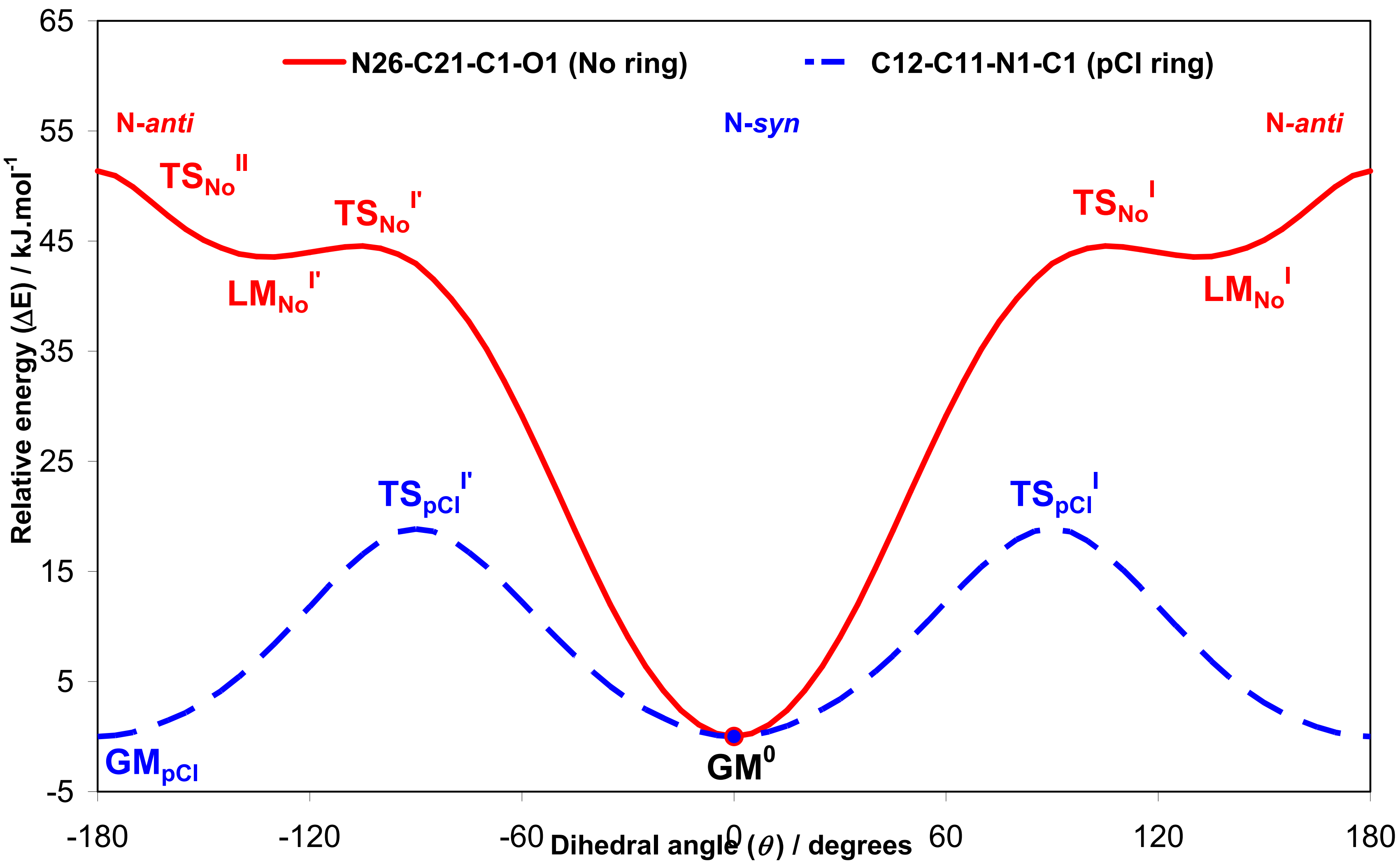

N26-C21-C1-O1 (No ring)
C12-C11-N1-C1 (pCl ring)
N-anti
N-syn
N-anti
TSNo II
TSNo I'
LMNo I'
TSNo I
LMNo I
TSpCl I'
TSpCl I
GMpCl
GM0
Relative energy (ΔE) / kJ.mol-1
65
55
45
35
25
15
5
-5
-180
-120
-60
0
60
120
180
Dihedral angle (θ) / degrees

NopCl

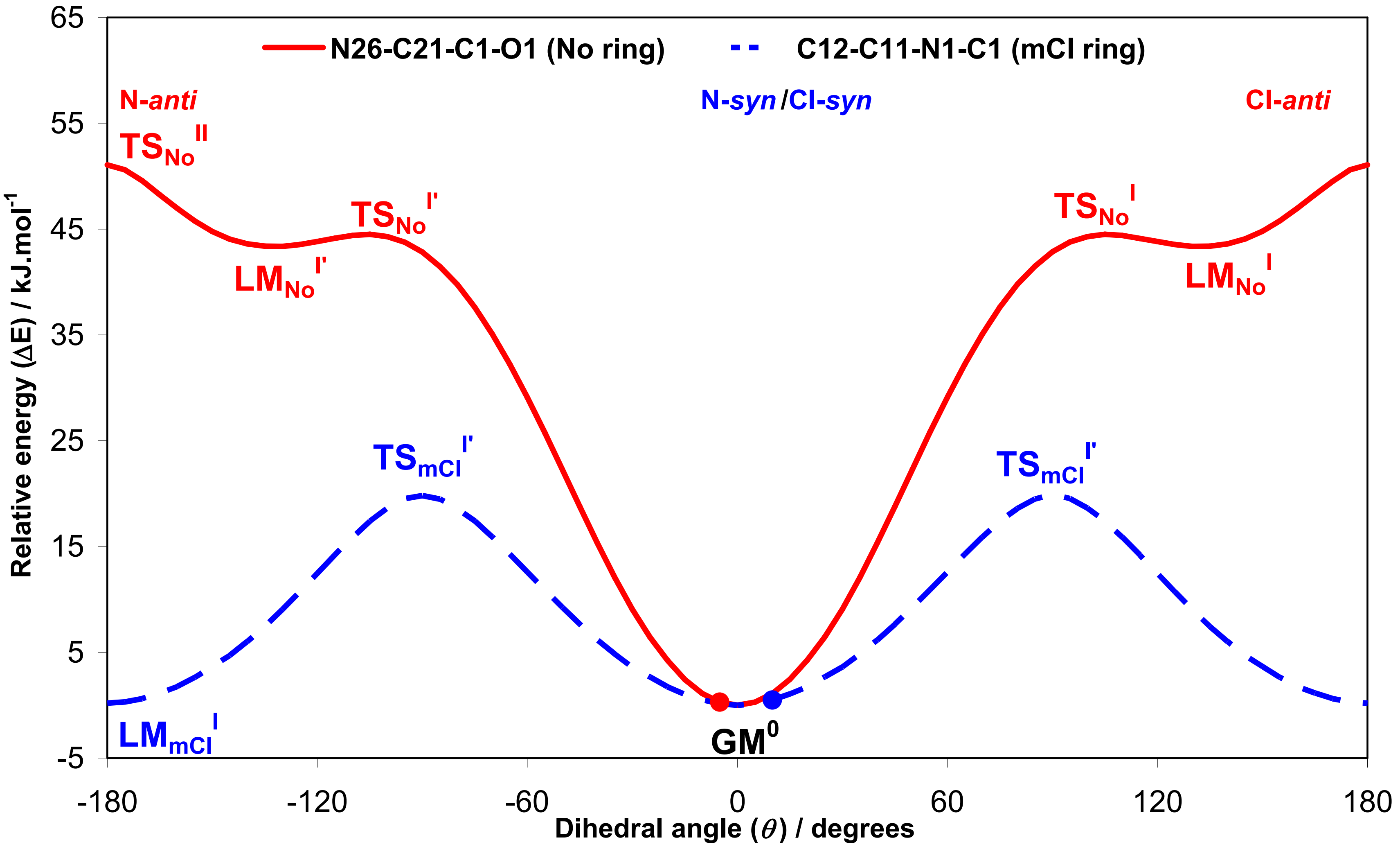
N26-C21-C1-O1 (No ring)
C12-C11-N1-C1 (mCl ring)
N-anti
N-syn /Cl-syn
Cl-anti
TSNo II
TSNo I'
LMNo I'
TSNo I
LMNo I
TSmCl I'
TSmCl I'
LMmCl I
GM0
Relative energy (ΔE) / kJ.mol-1
Dihedral angle (θ) / degrees
65
55
45
35
25
15
5
-5
-180
-120
-60
0
60
120
180

NomCl

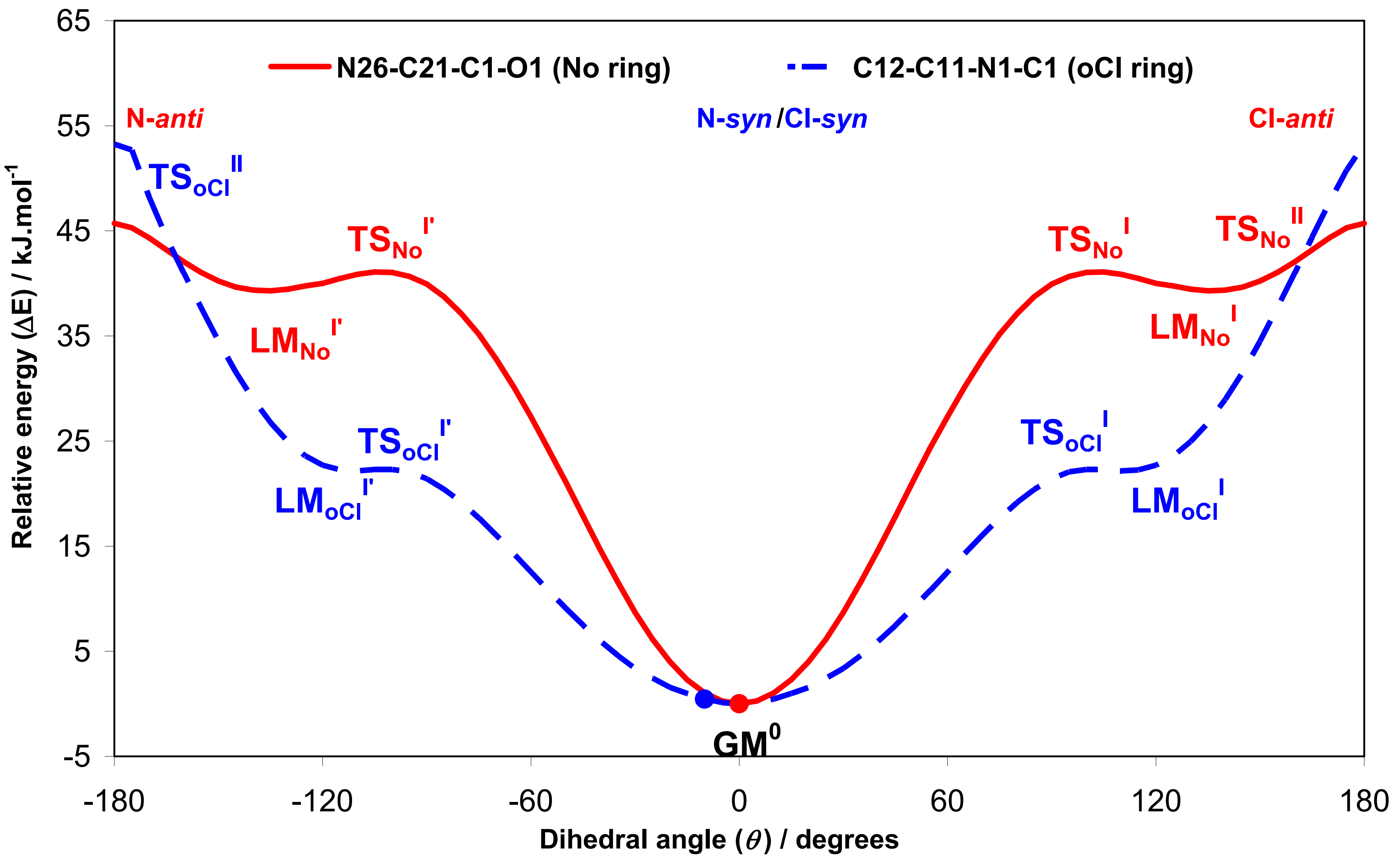
N26-C21-C1-O1 (No ring)
C12-C11-N1-C1 (oCl ring)
N-anti
N-syn /Cl-syn
Cl-anti
TSoCl II
TSNo I'
TSNo I
TSNo II
LMNo I'
LMNo I
TSoCl I'
TSoCl I
LMoCl I'
LMoCl I
GM0
Relative energy (ΔE) / kJ.mol-1
Dihedral angle (θ) / degrees
65
55
45
35
25
15
5
-5
-180
-120
-60
0
60
120
180

NooCl

Section III

ATR-IR and NMR spectral data for nine NxxCl isomers ($CDCl_3$ and DMSO-d_6 used) (from NppCl to NooCl)

NppCl data

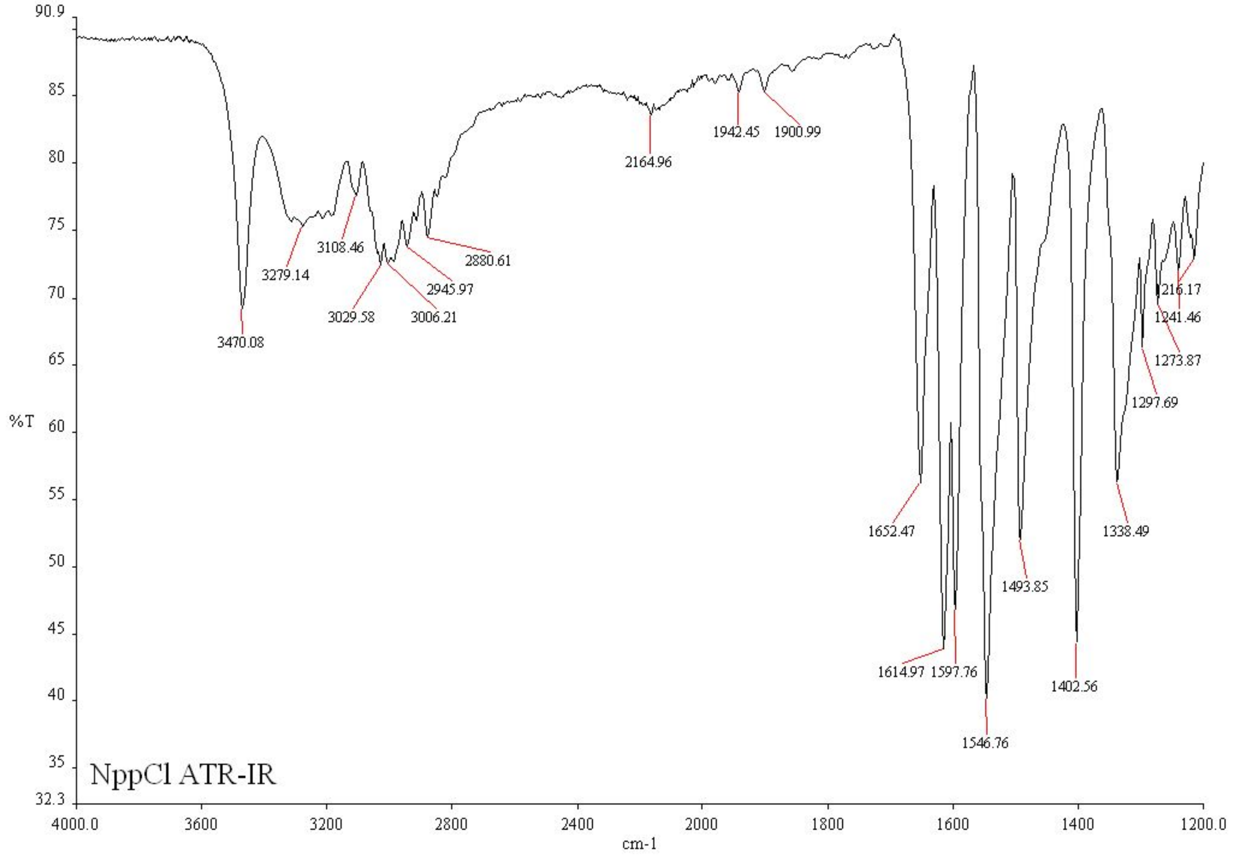

Spectrum 1: ATR-IR spectrum of **NppCl**

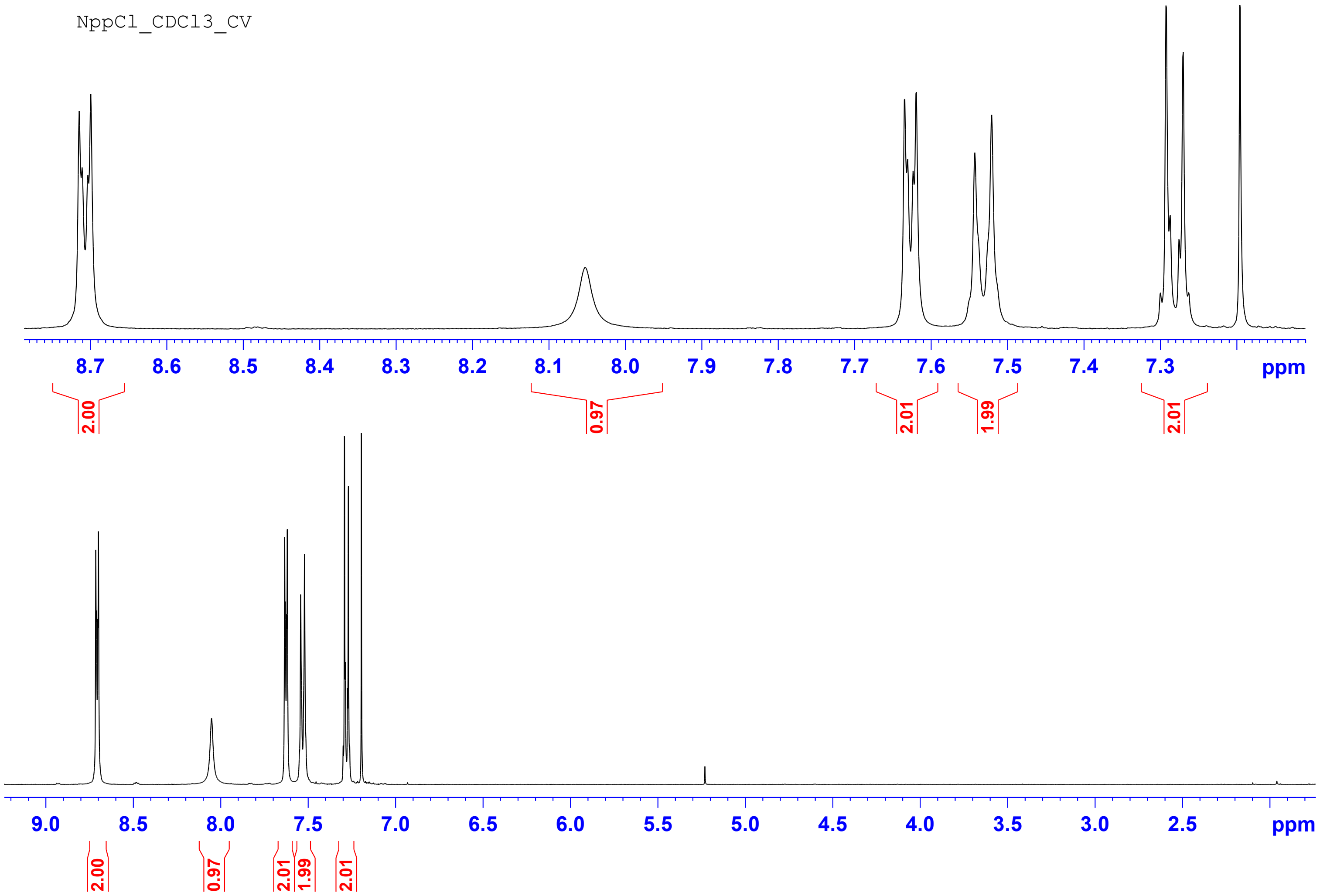

Spectrum 2: ^{1}H-NMR of **NppCl** in $CDCl_3$

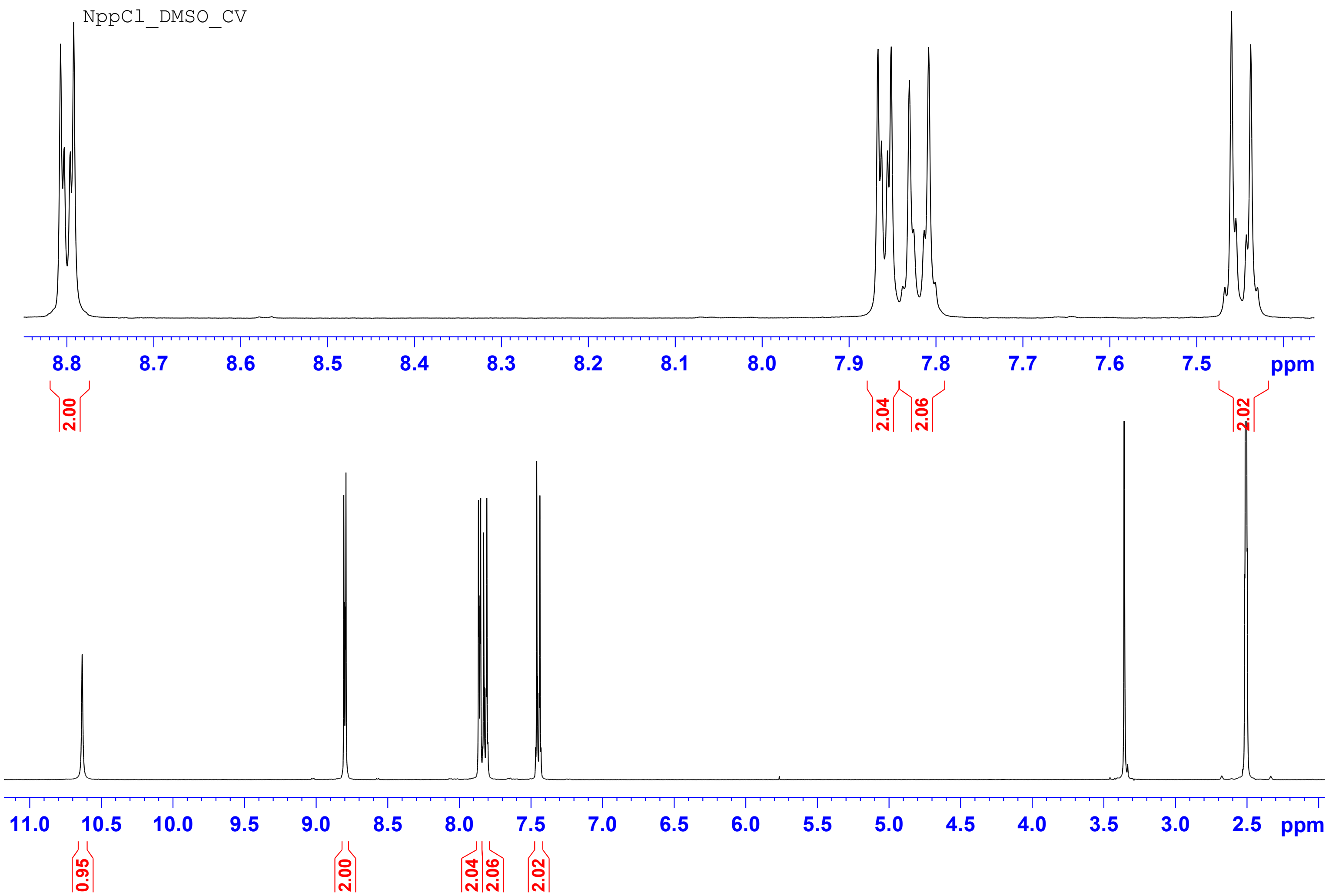

Spectrum 3: ^{1}H-NMR of **NppCl** in DMSO-d_6

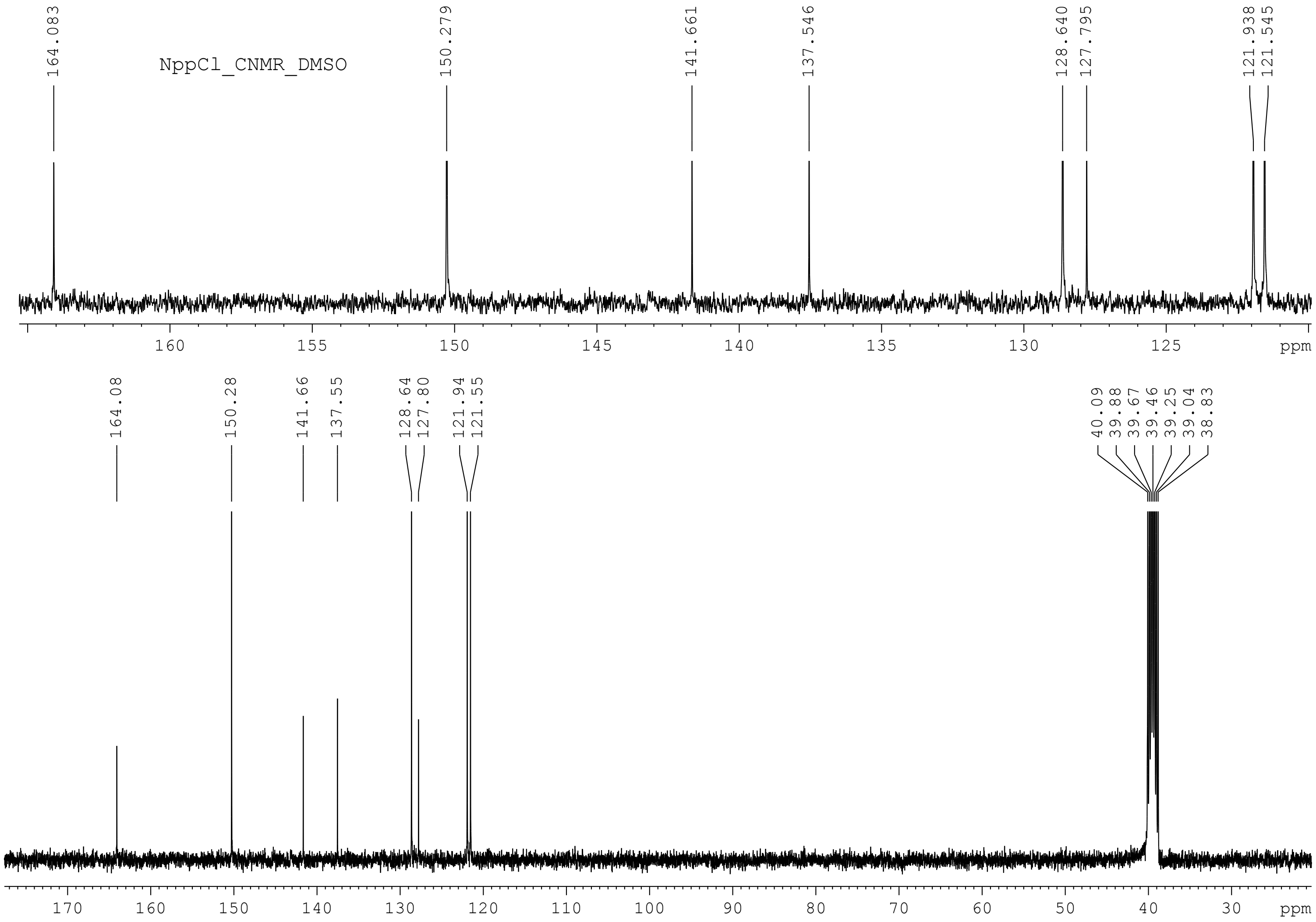

Spectrum 4: ^{13}C-NMR in DMSO-d_6 of **NppCl**

NpmCl data

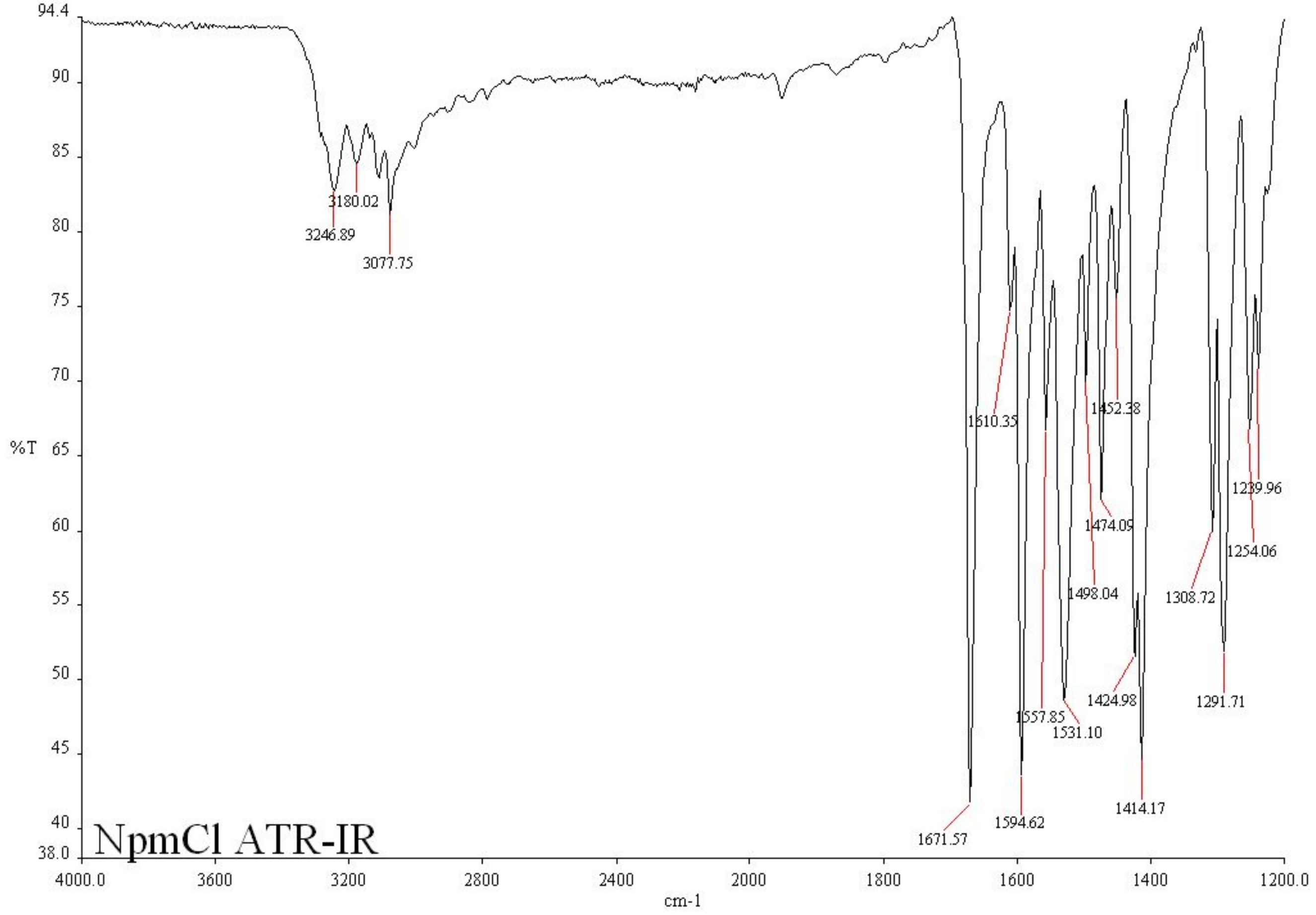

Spectrum 5: ATR-IR spectrum of **NpmCl**

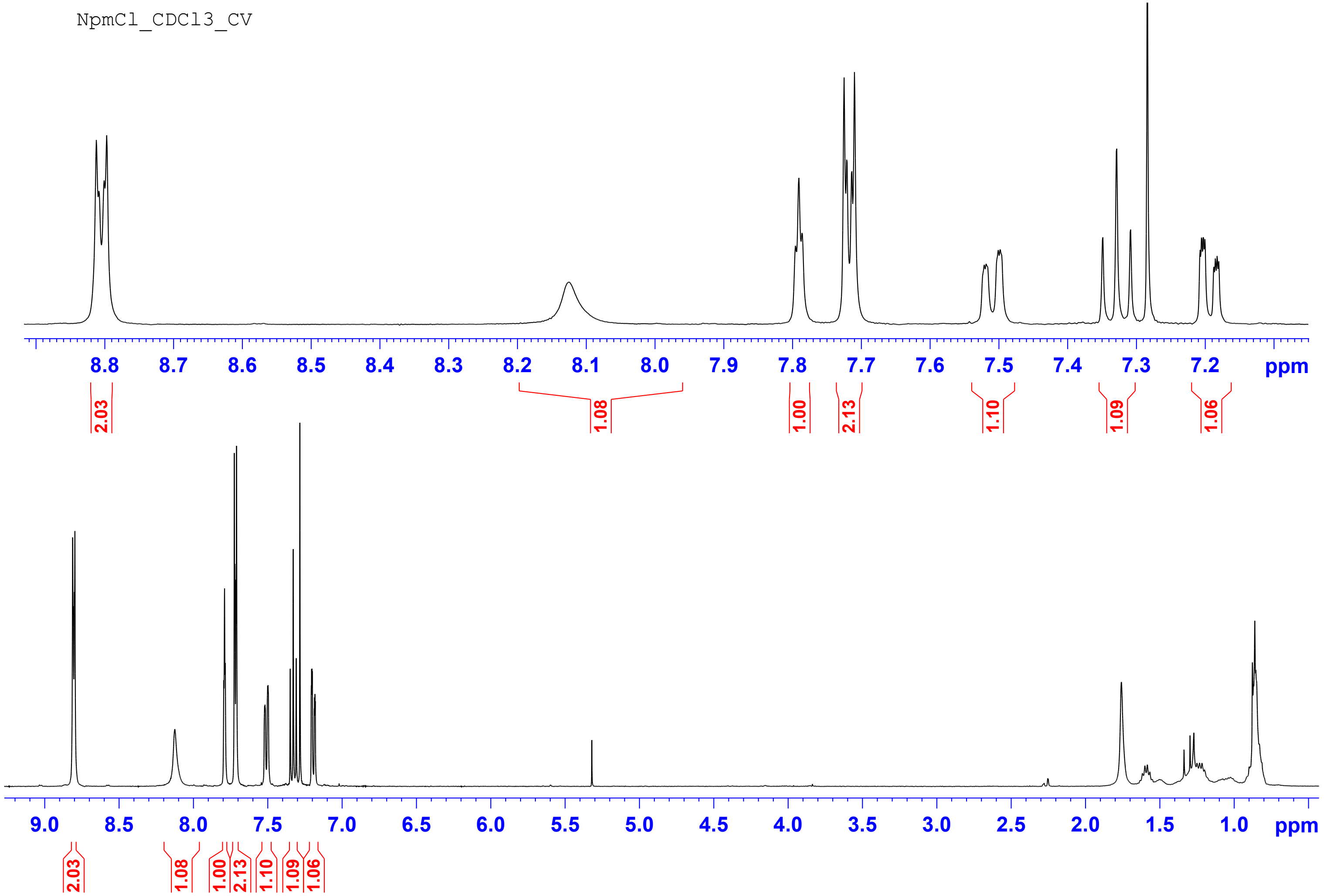

Spectrum 6: ^{1}H-NMR of **NpmCl** in $CDCl_3$

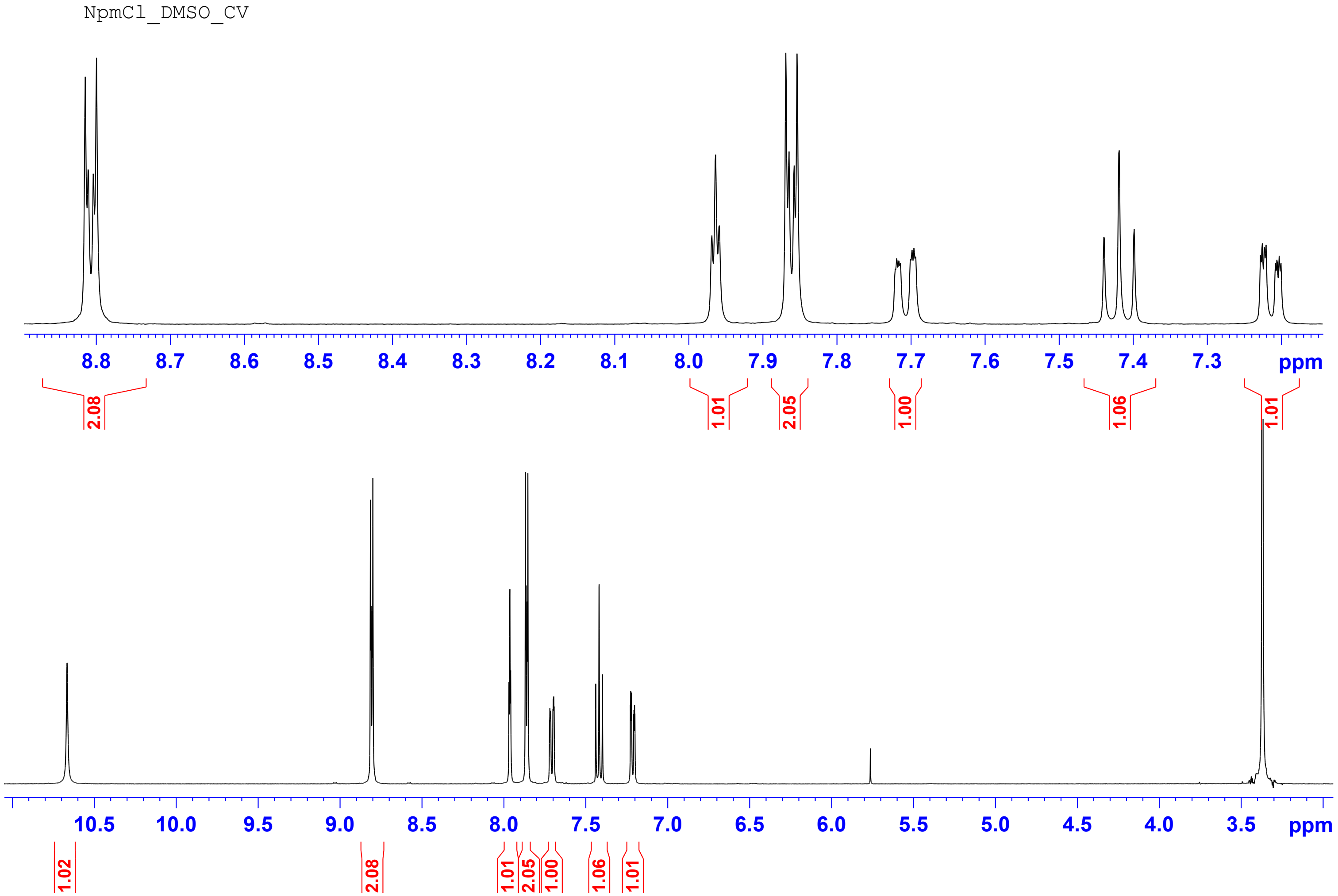

Spectrum 7: ^{1}H-NMR of **NpmCl** in DMSO-d_6

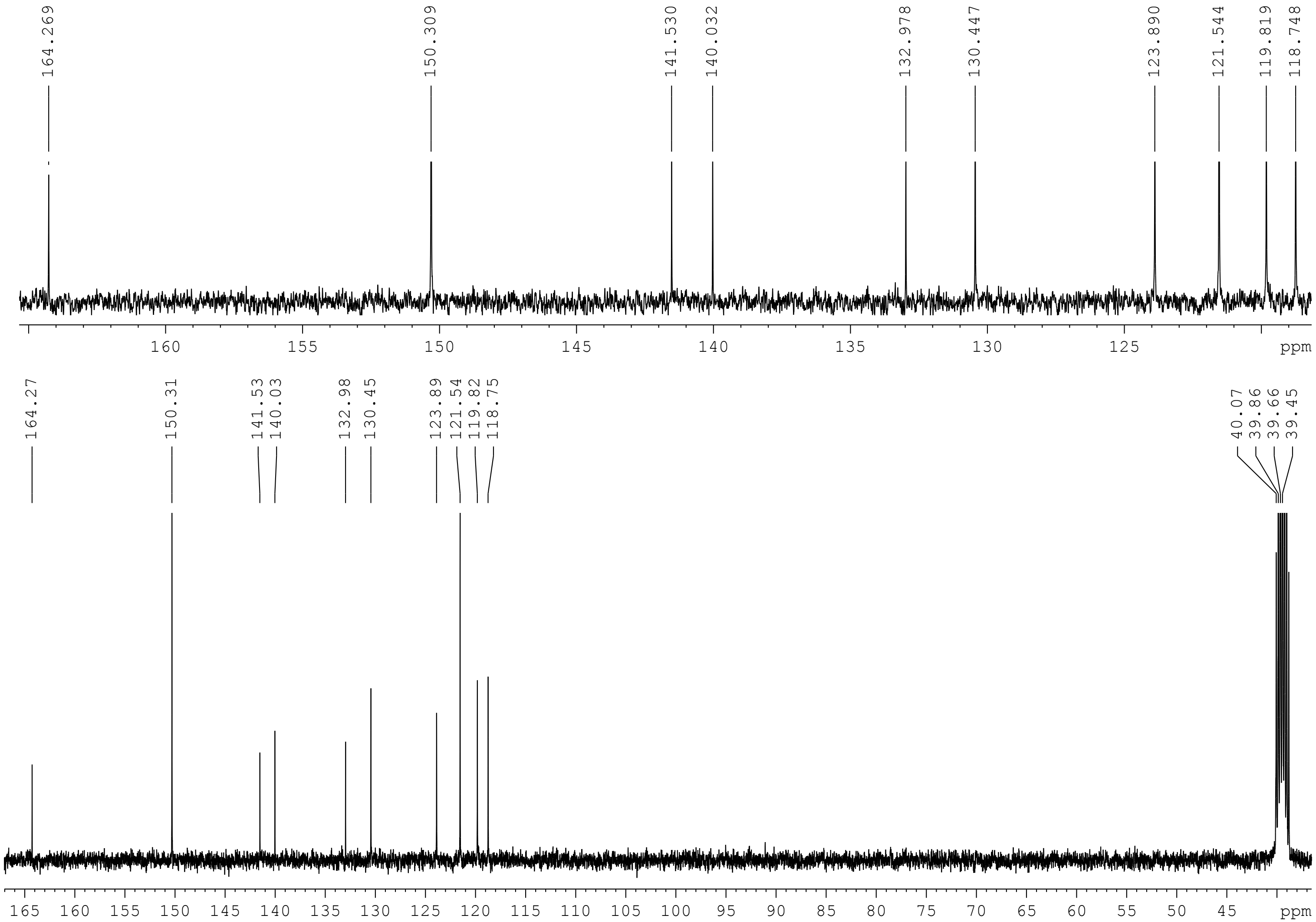

Spectrum 8: ^{13}C-NMR of **NpmCl** in DMSO-d_6

NpoCl data

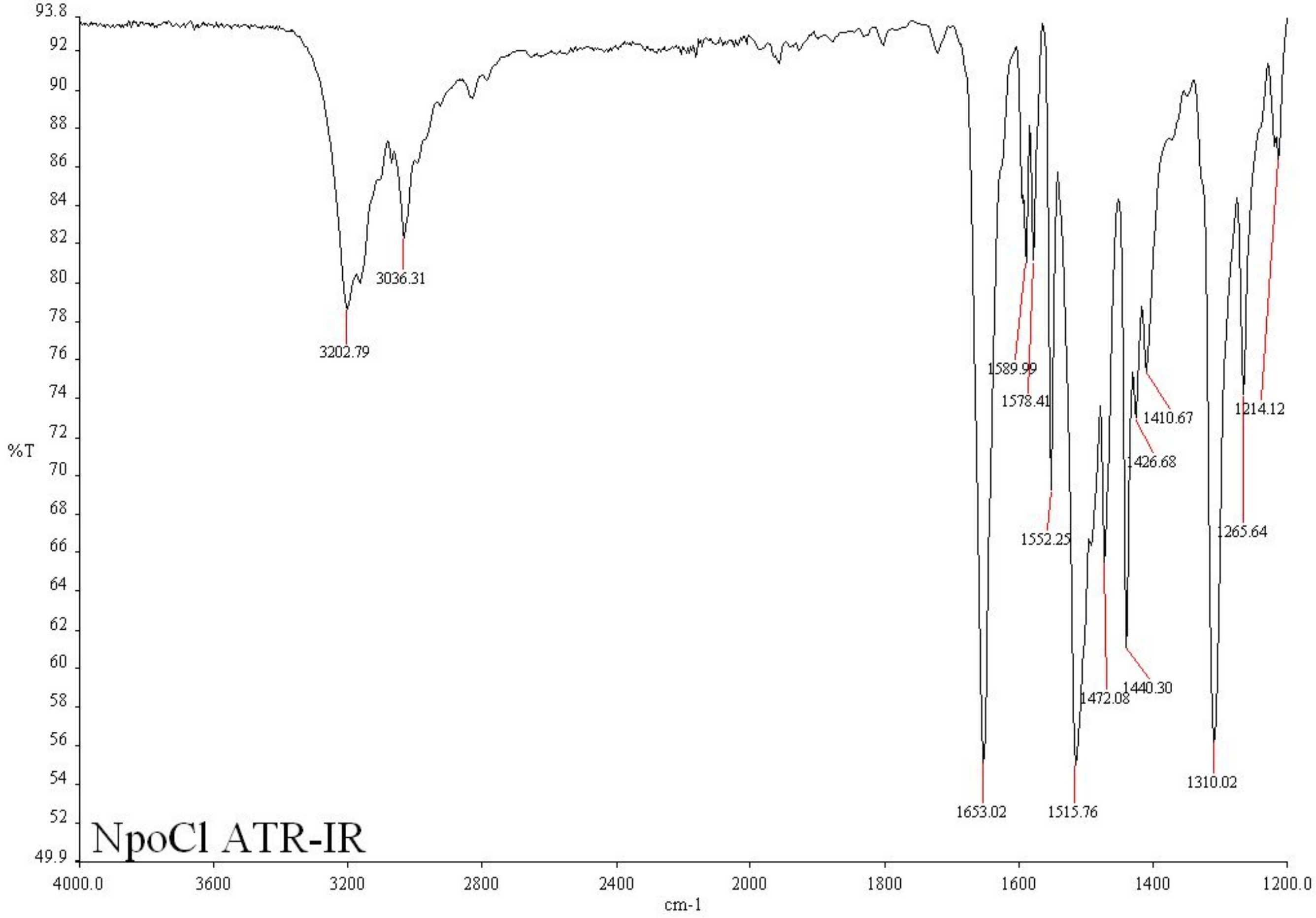

Spectrum 9: ATR-IR spectrum of **NpoCl**

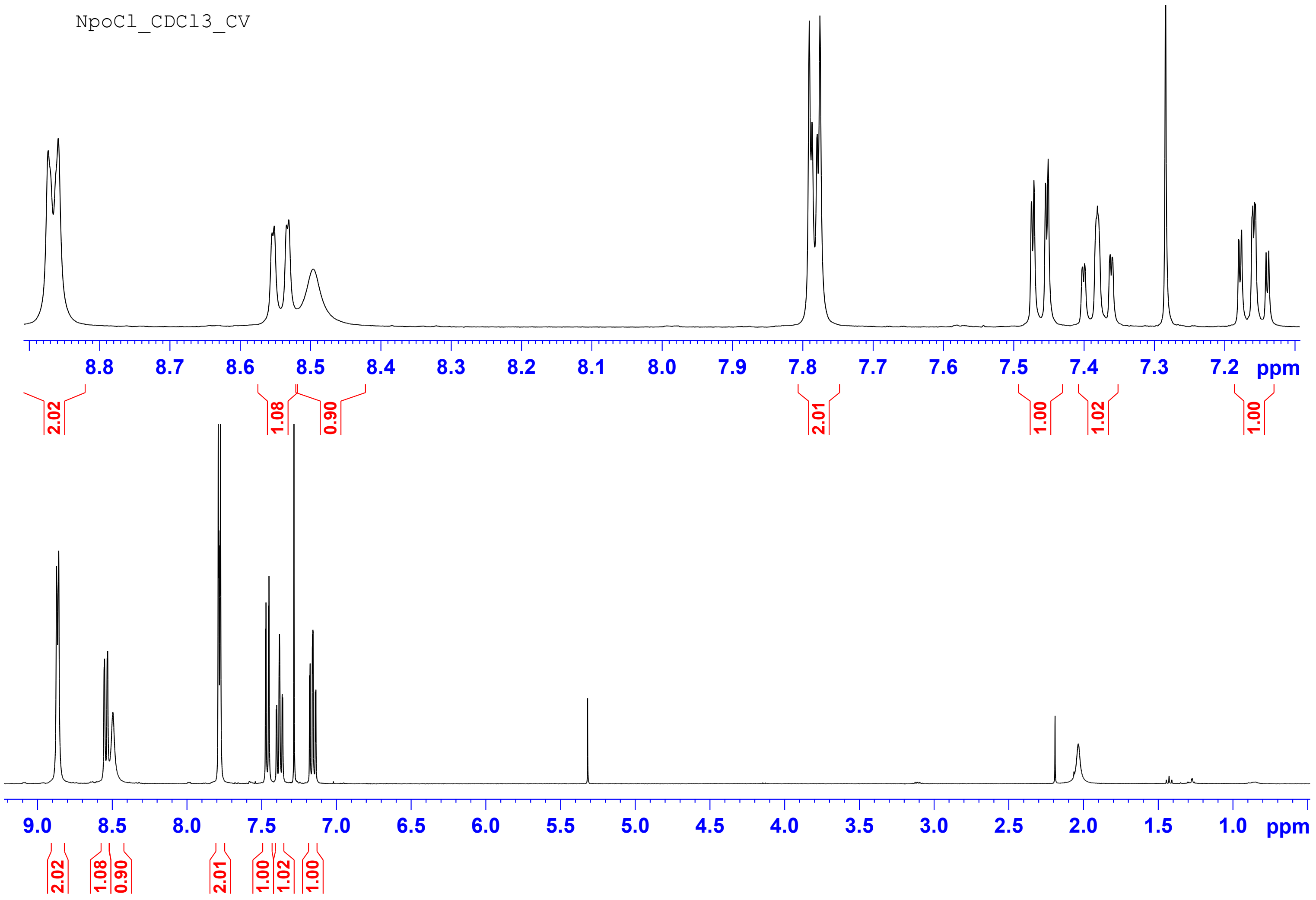

Spectrum 10: ^{1}H-NMR of **NpoCl** in $CDCl_3$

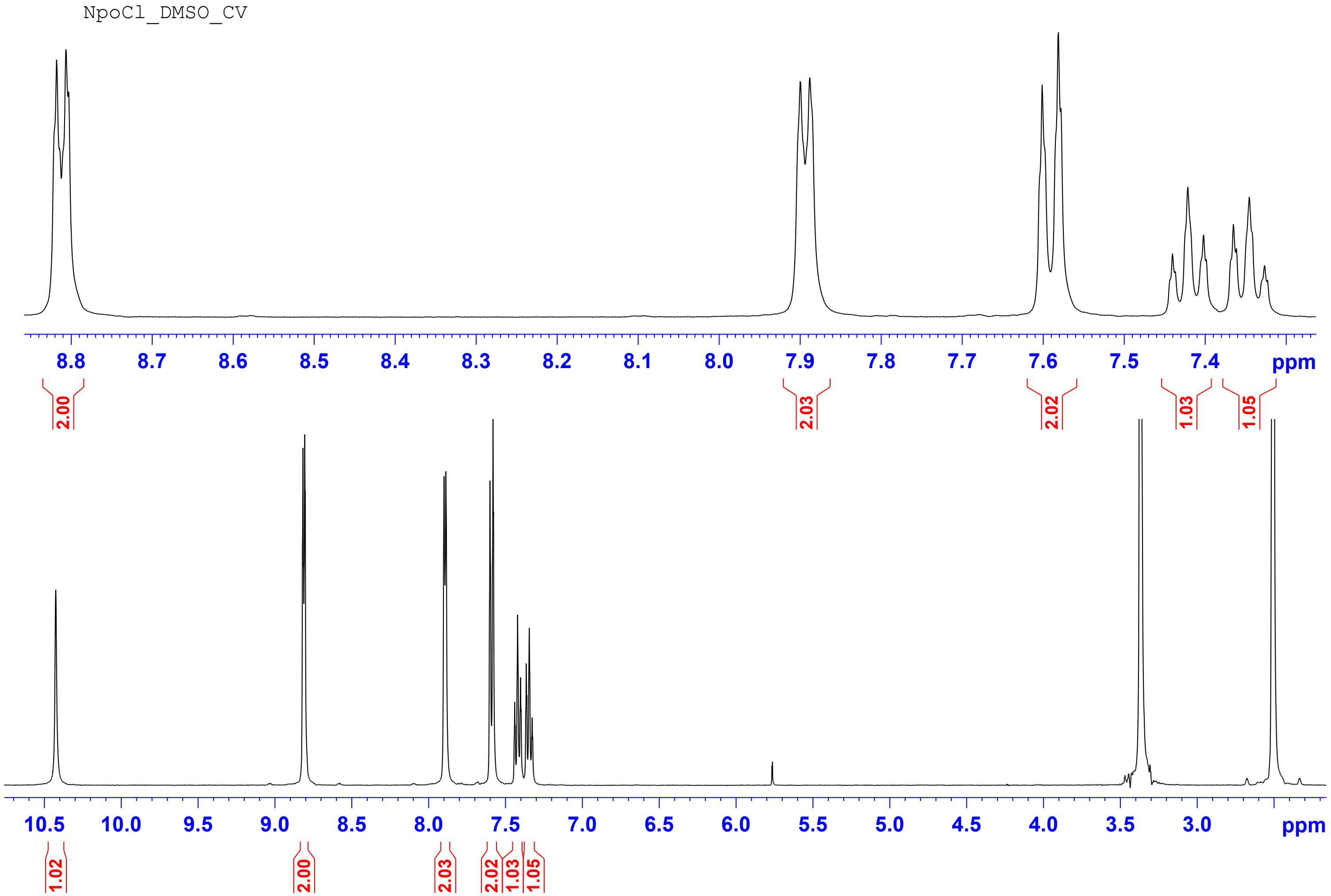

Spectrum 11: ^{1}H-NMR of **NpoCl** in DMSO-d_6

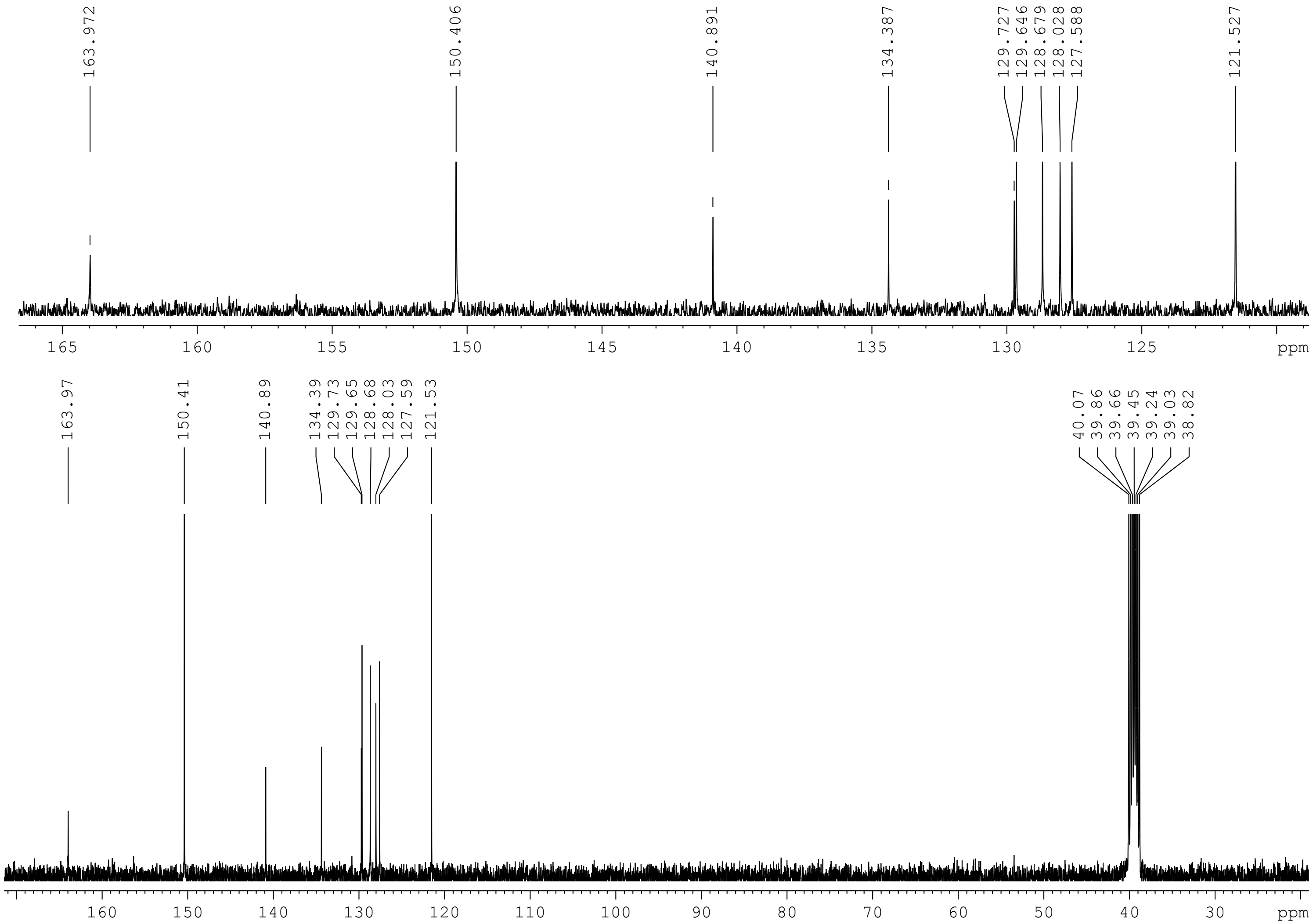

Spectrum 12: ^{13}C-NMR of **NpoCl** in DMSO-d_6

NmpCl data

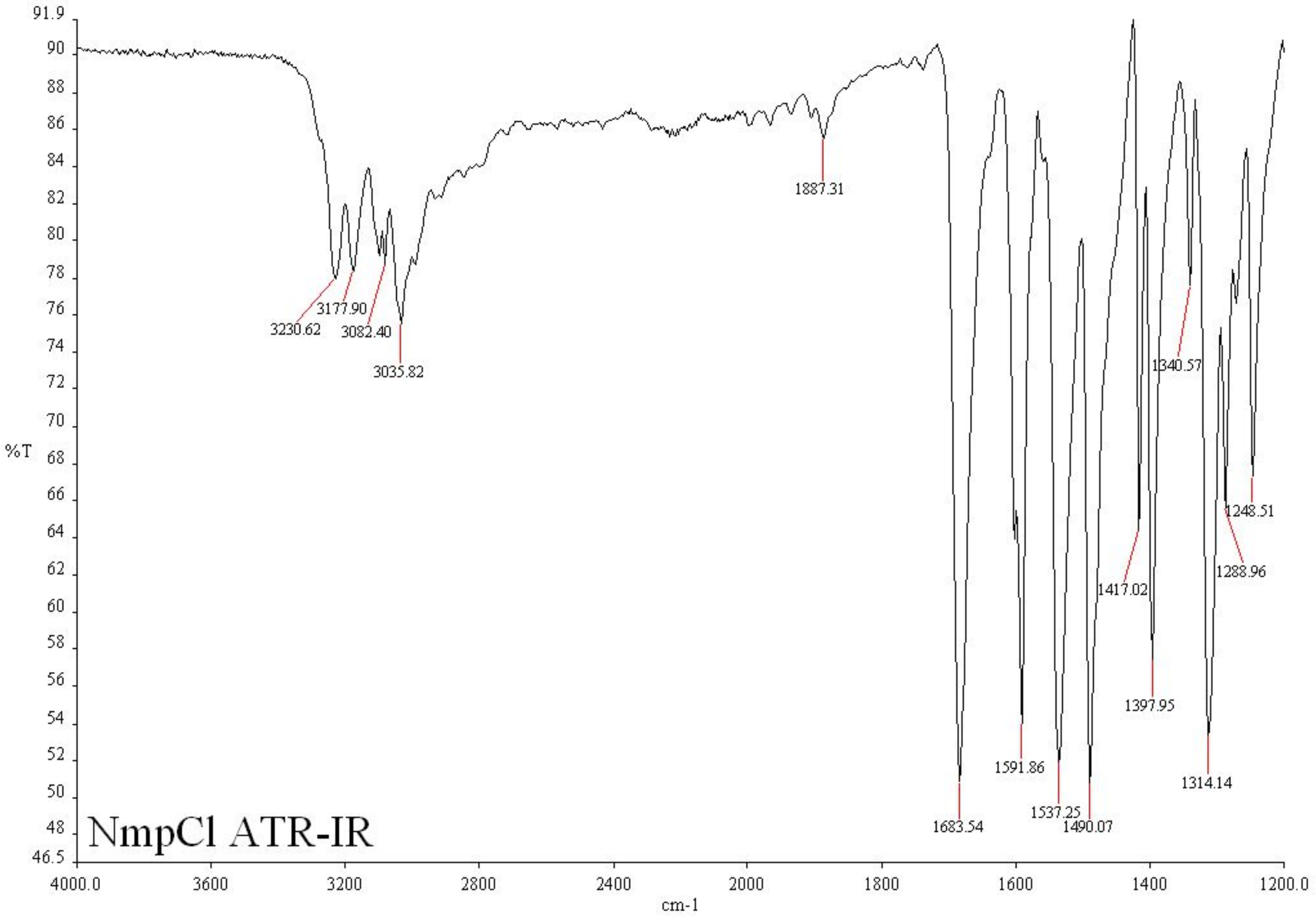

Spectrum 13: ATR-IR spectrum of **NmpCl**

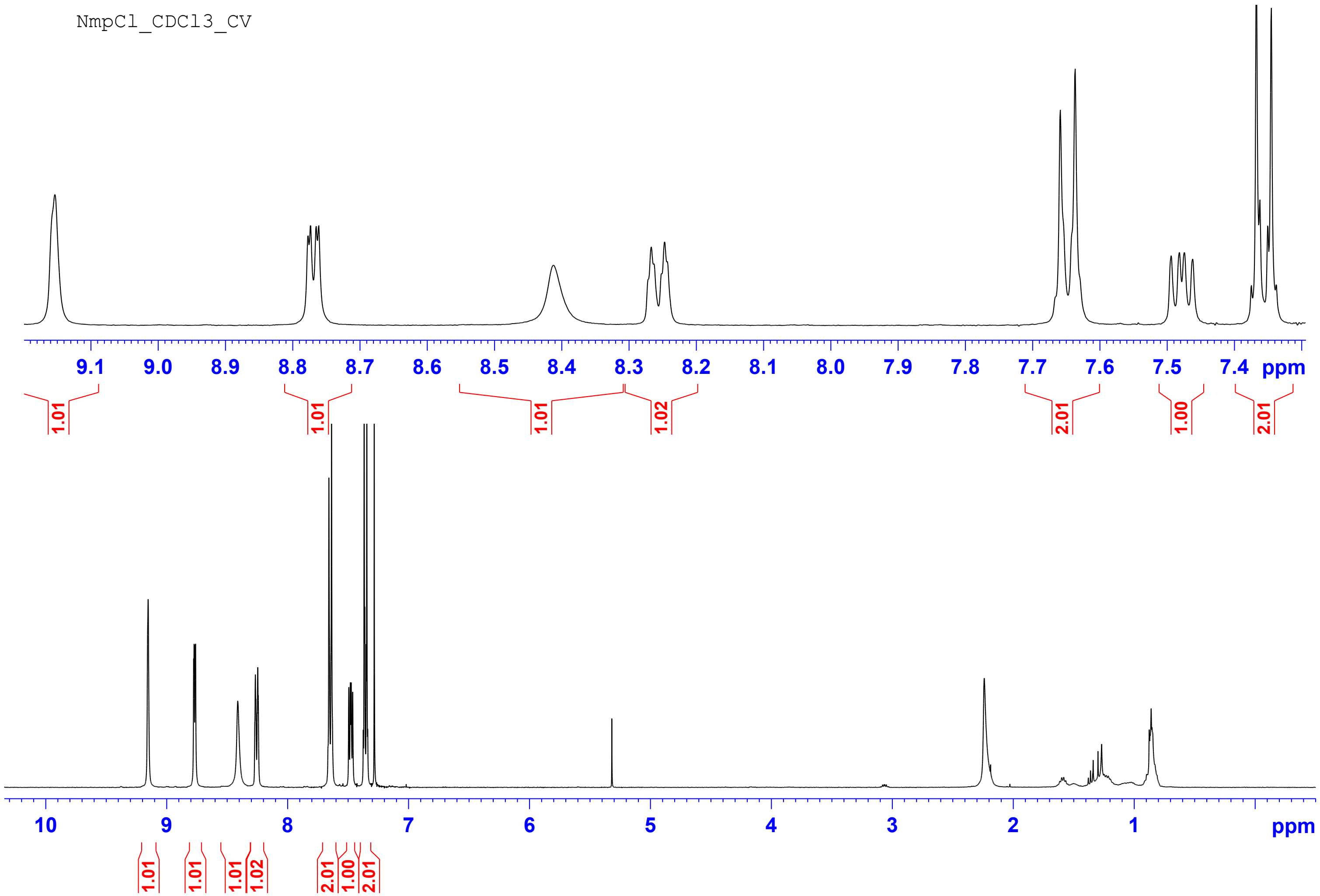

Spectrum 14: ^{1}H-NMR of **NmpCl** in $CDCl_3$

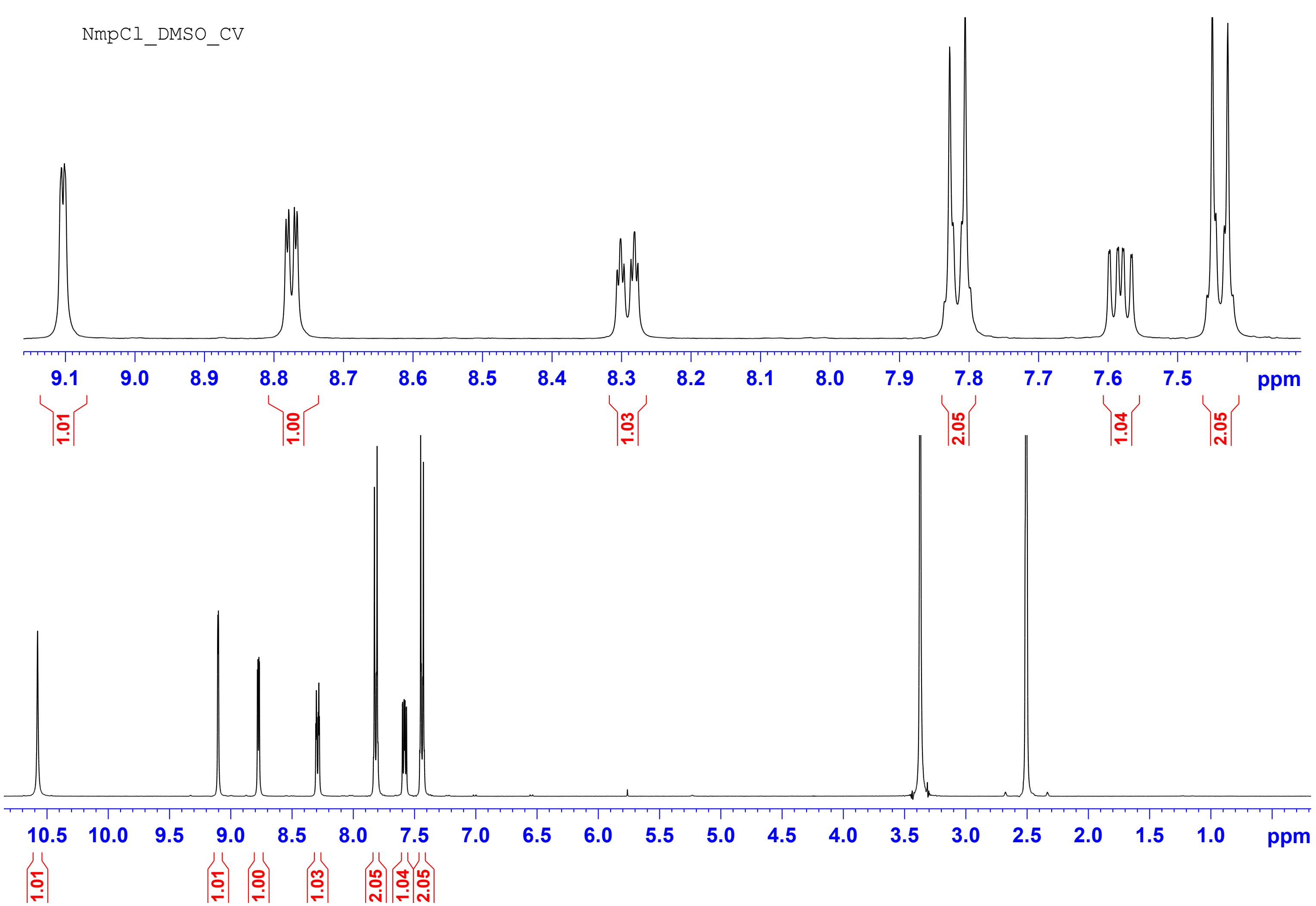

Spectrum 15: ^{1}H-NMR of **NmpCl** in DMSO-d_6

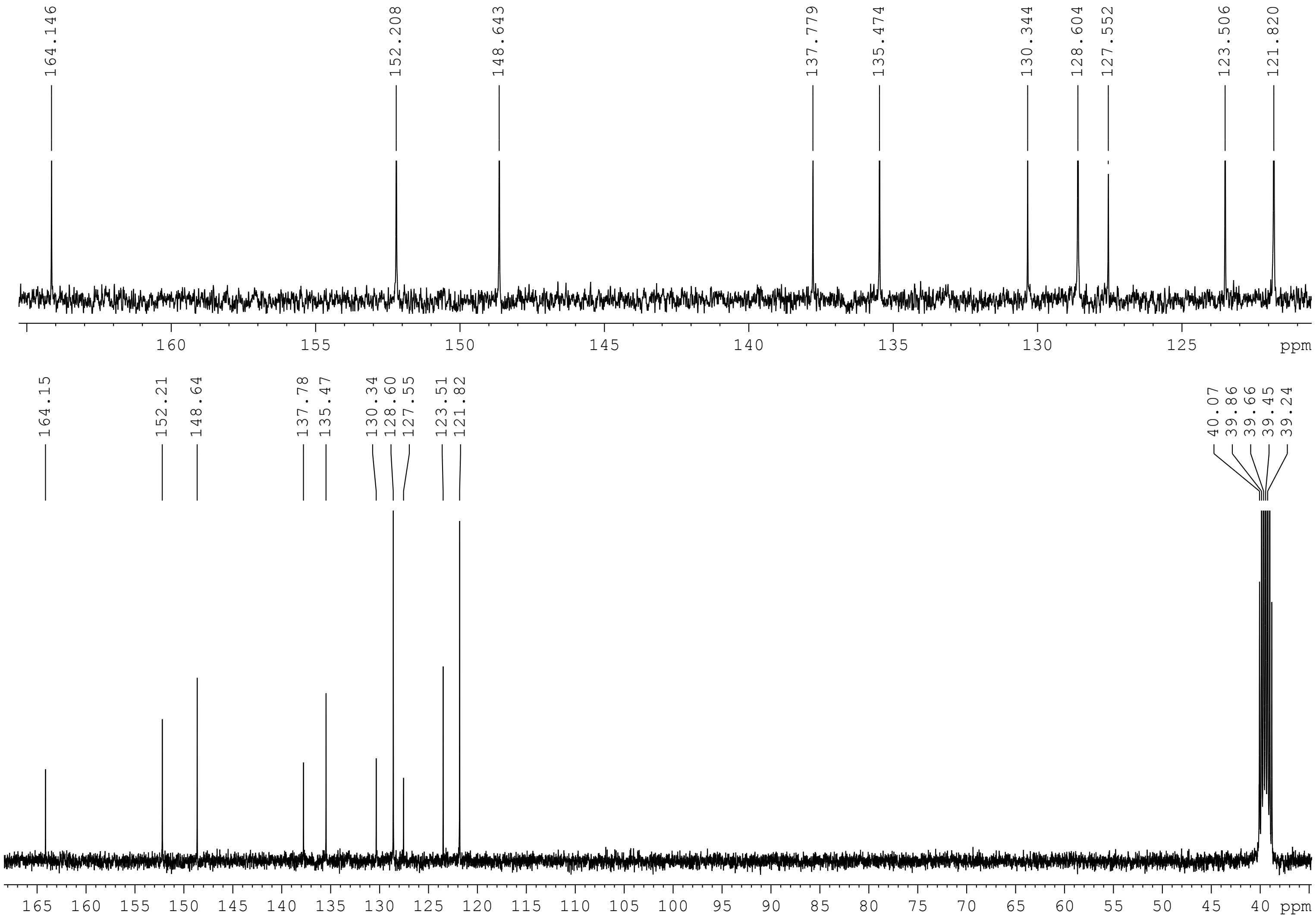

Spectrum 16: ^{13}C-NMR of **NmpCl** in DMSO-d_6

NmmCl data

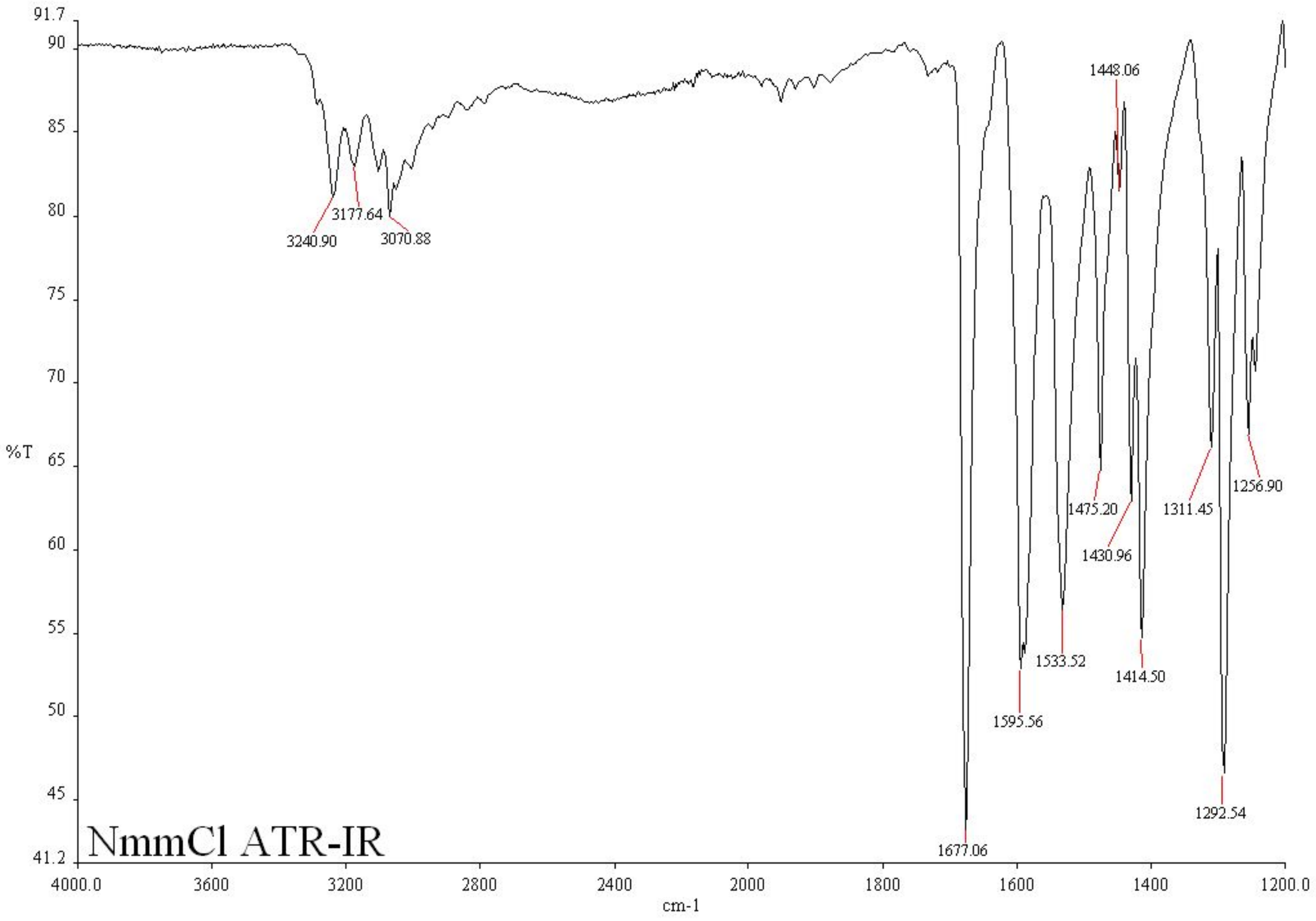

Spectrum 17: ATR-IR spectrum of **NmmCl**

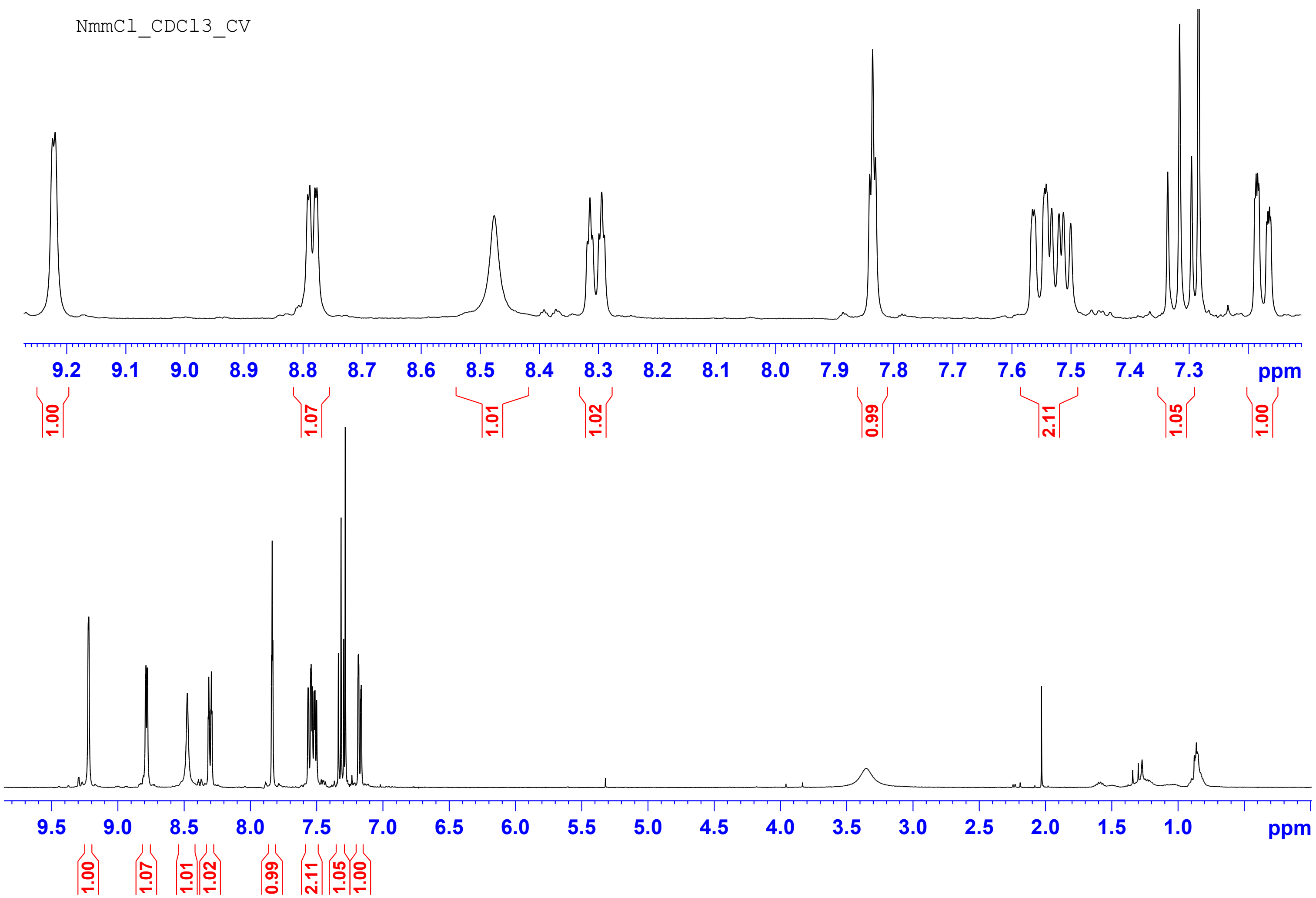

Spectrum 18: ^{1}H-NMR of **NmmCl** in $CDCl_3$

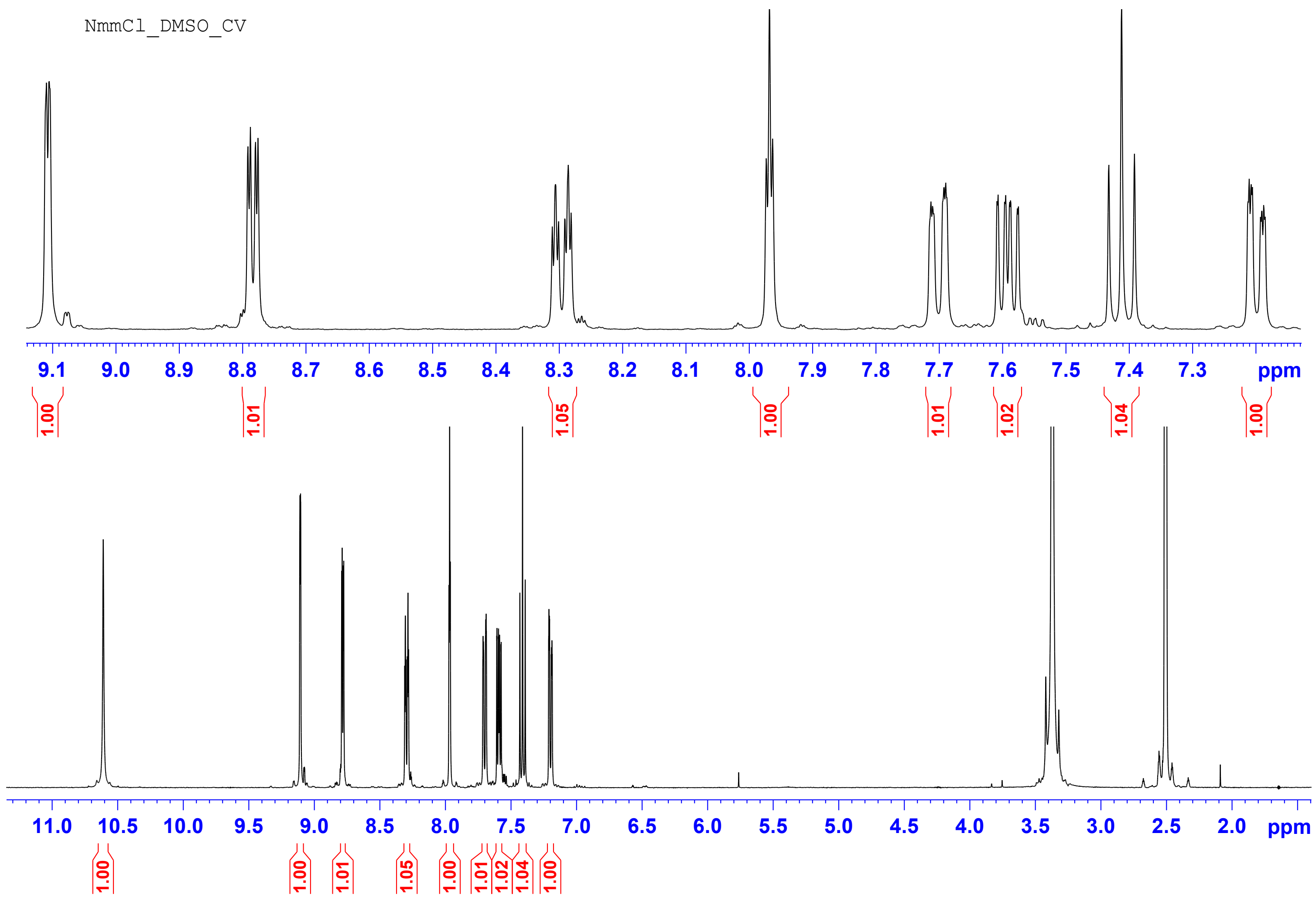

Spectrum 19: ^{1}H-NMR of **NmmCl** in DMSO-d_6

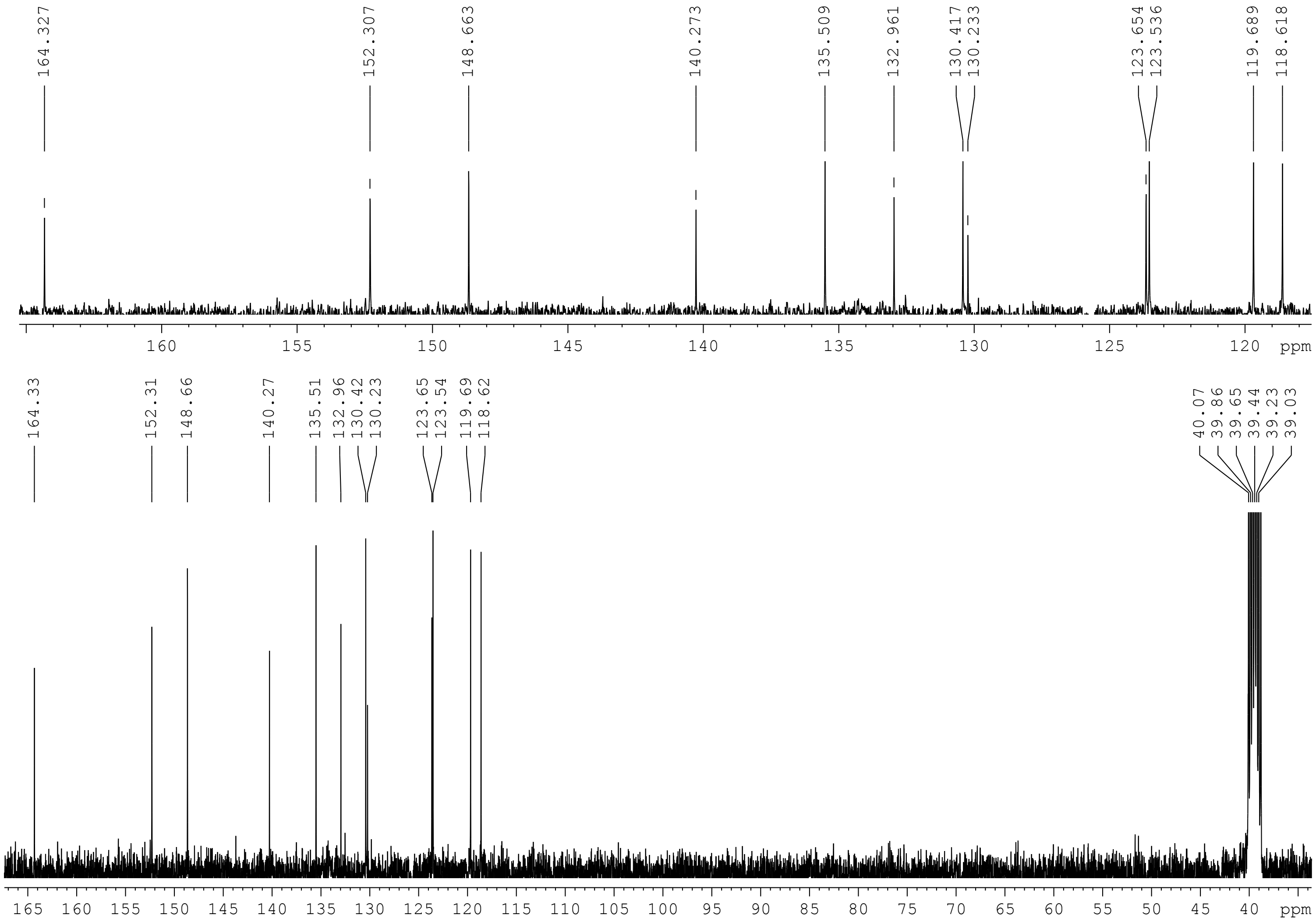

Spectrum 20: ^{13}C-NMR of **NmmCl** in DMSO-d_6

NmoCl data

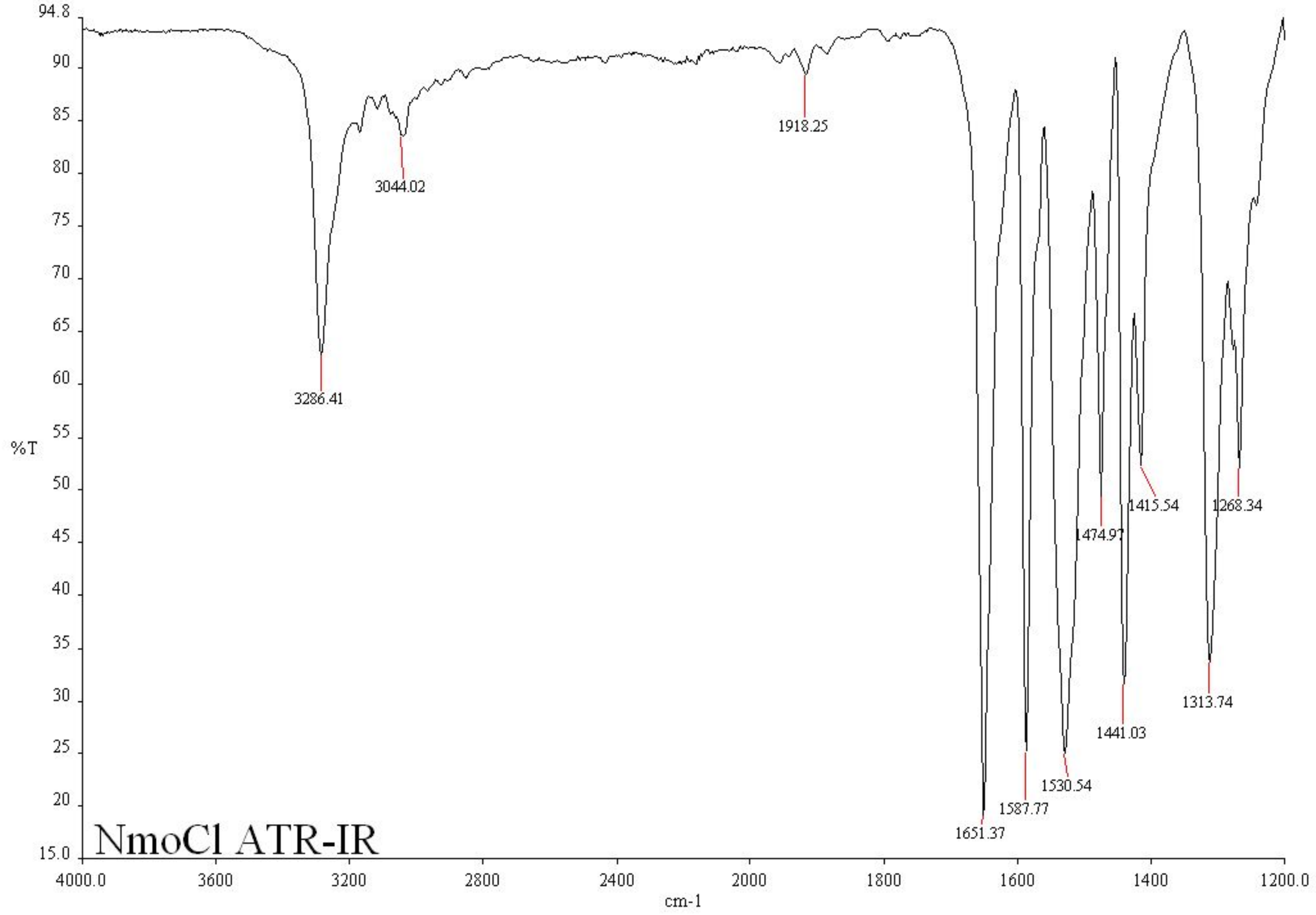

Spectrum 21: ATR-IR spectrum of **NmoCl**

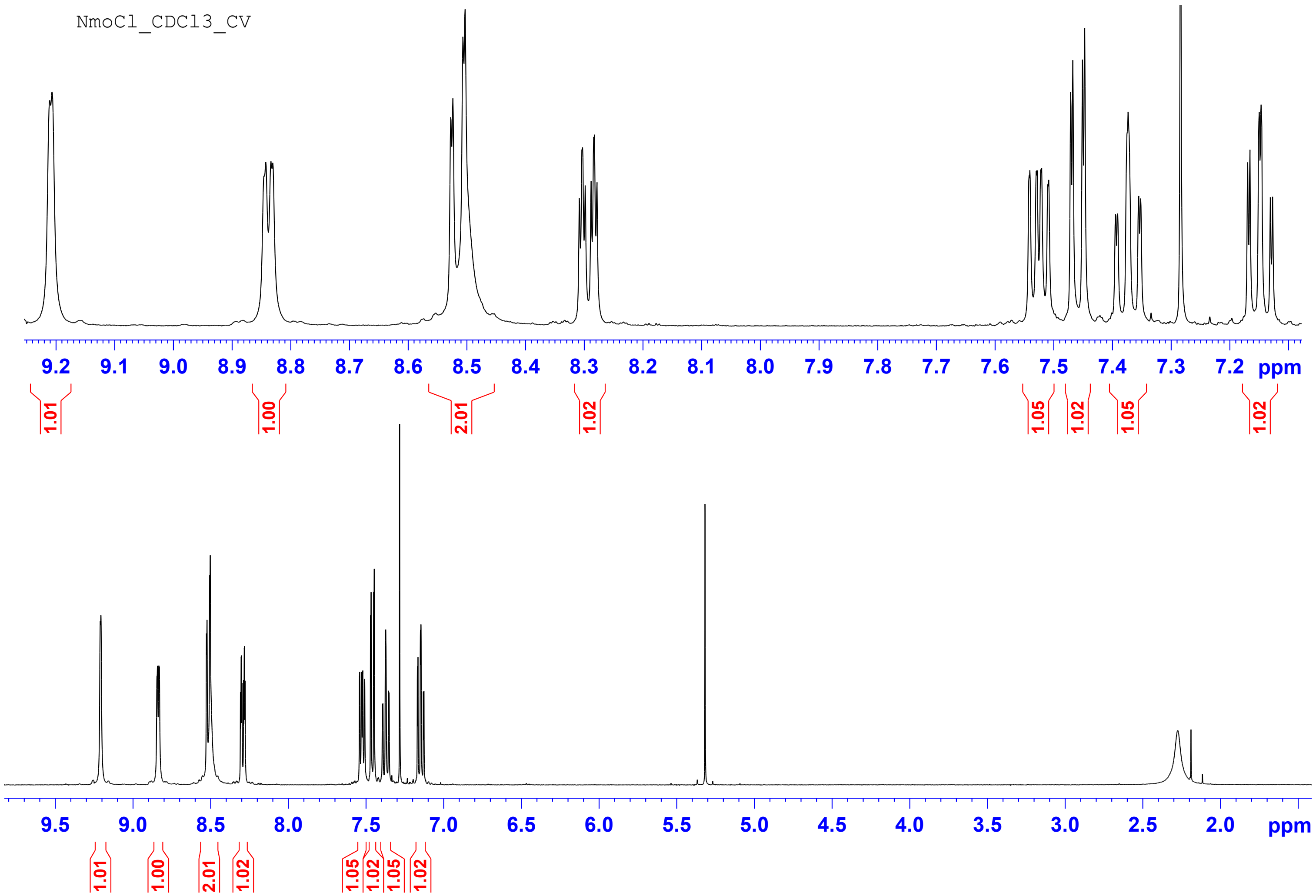

Spectrum 22: ^{1}H-NMR of **NmoCl** in $CDCl_3$

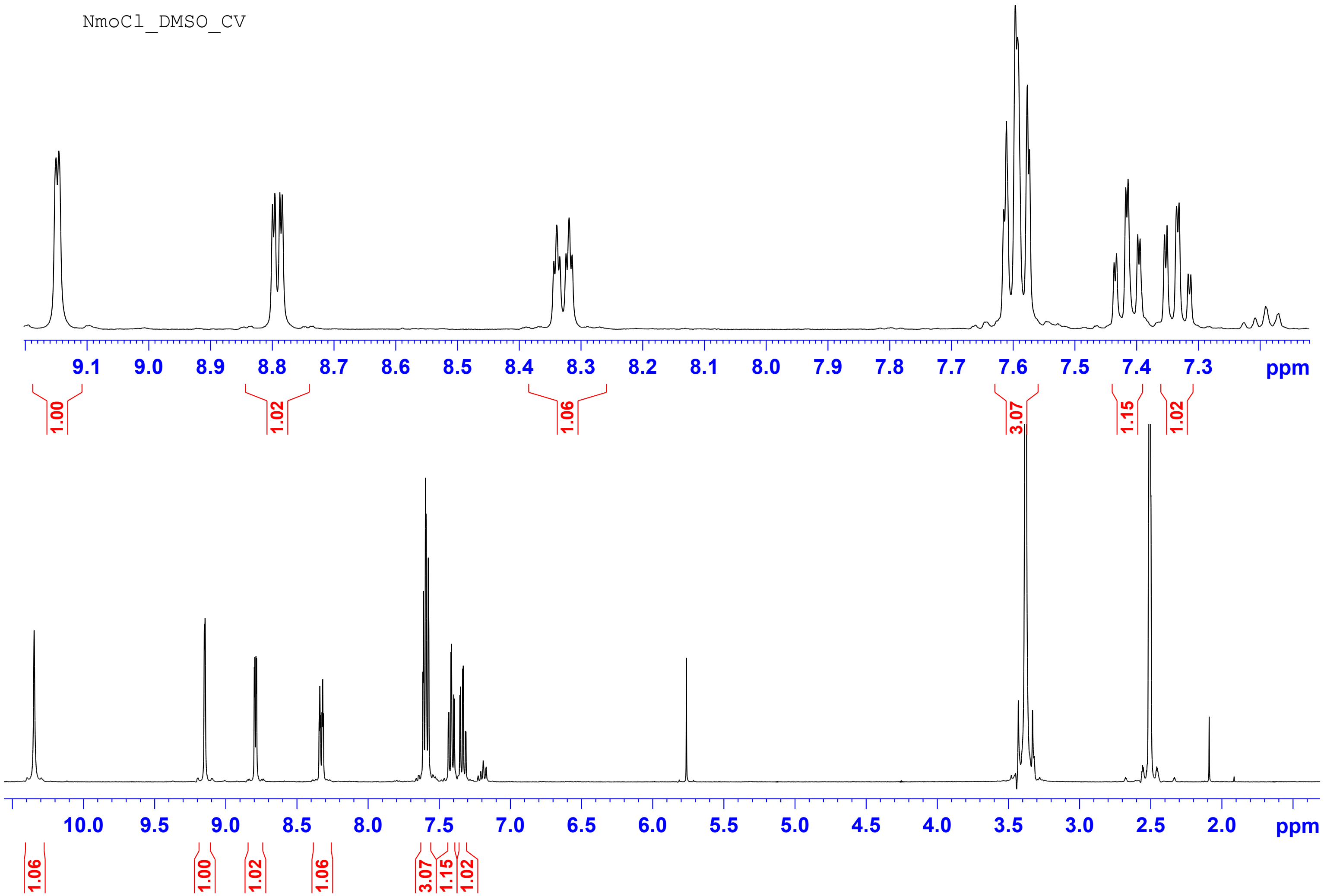

Spectrum 23: ^{1}H-NMR of **NmoCl** in DMSO-d_6

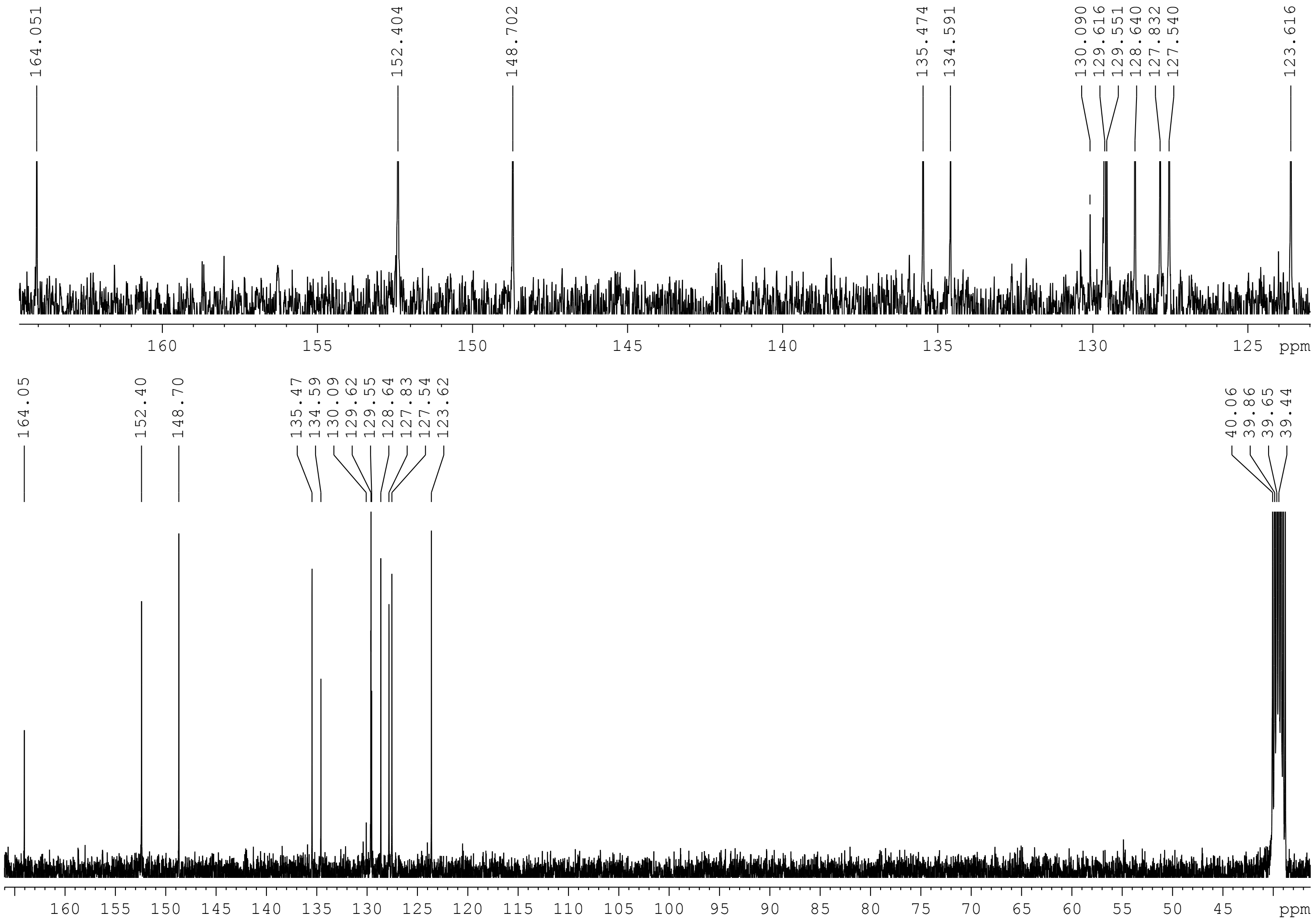

Spectrum 24: ^{13}C-NMR of **NmoCl** in DMSO-d_6

NopCl data

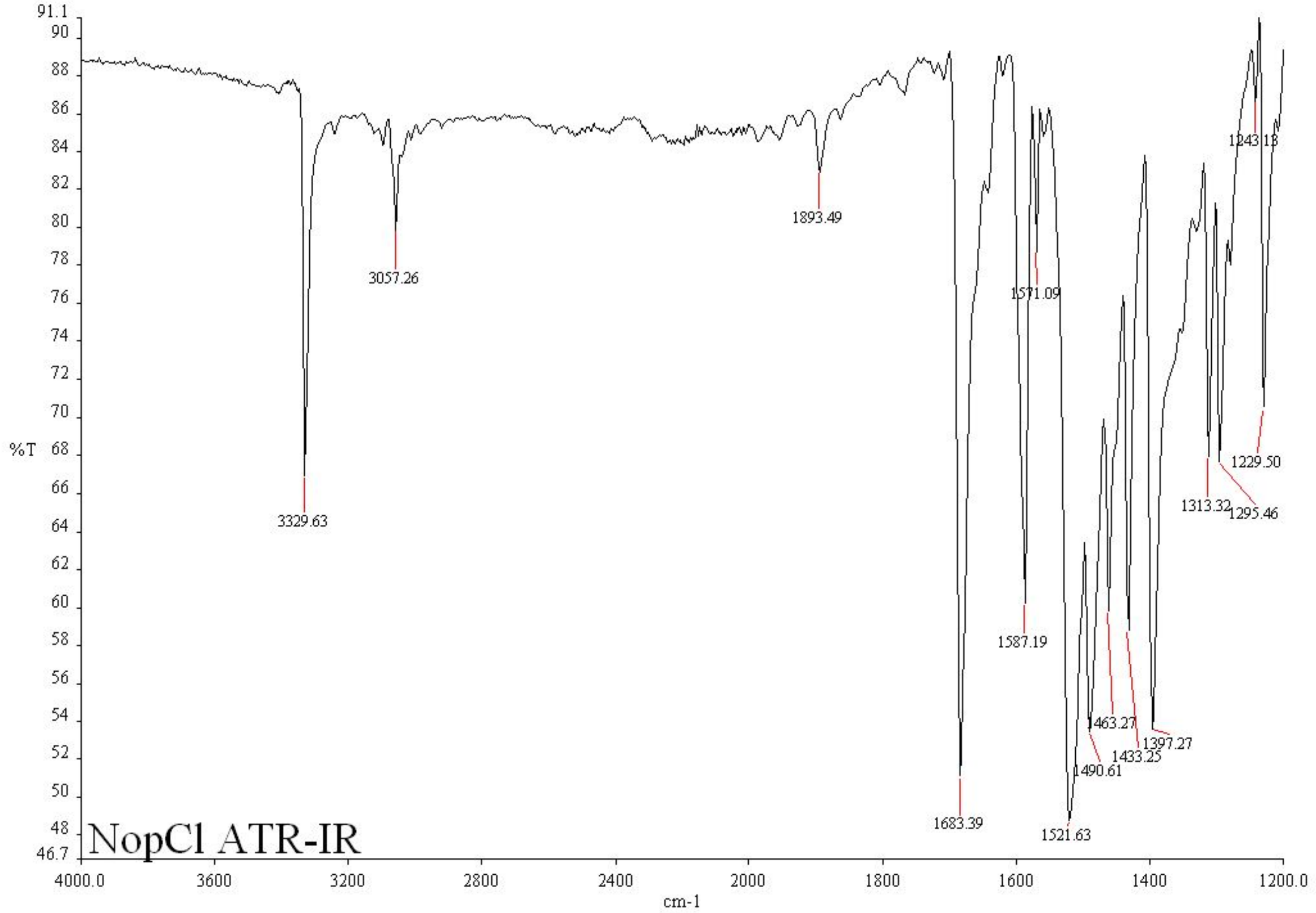

Spectrum 25: ATR-IR spectrum of **NopCl**

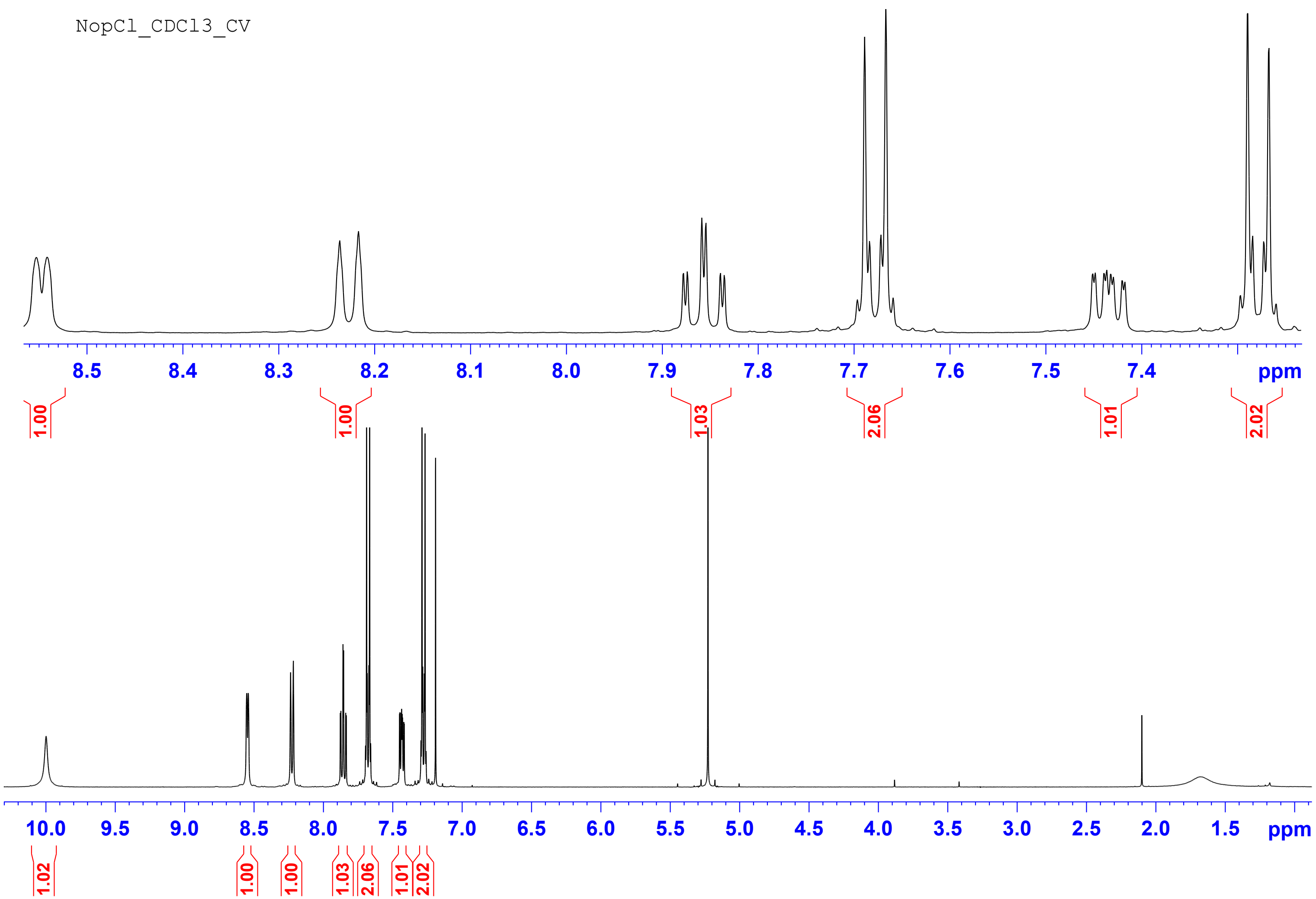

Spectrum 26: ^{1}H-NMR of **NopCl** in $CDCl_3$

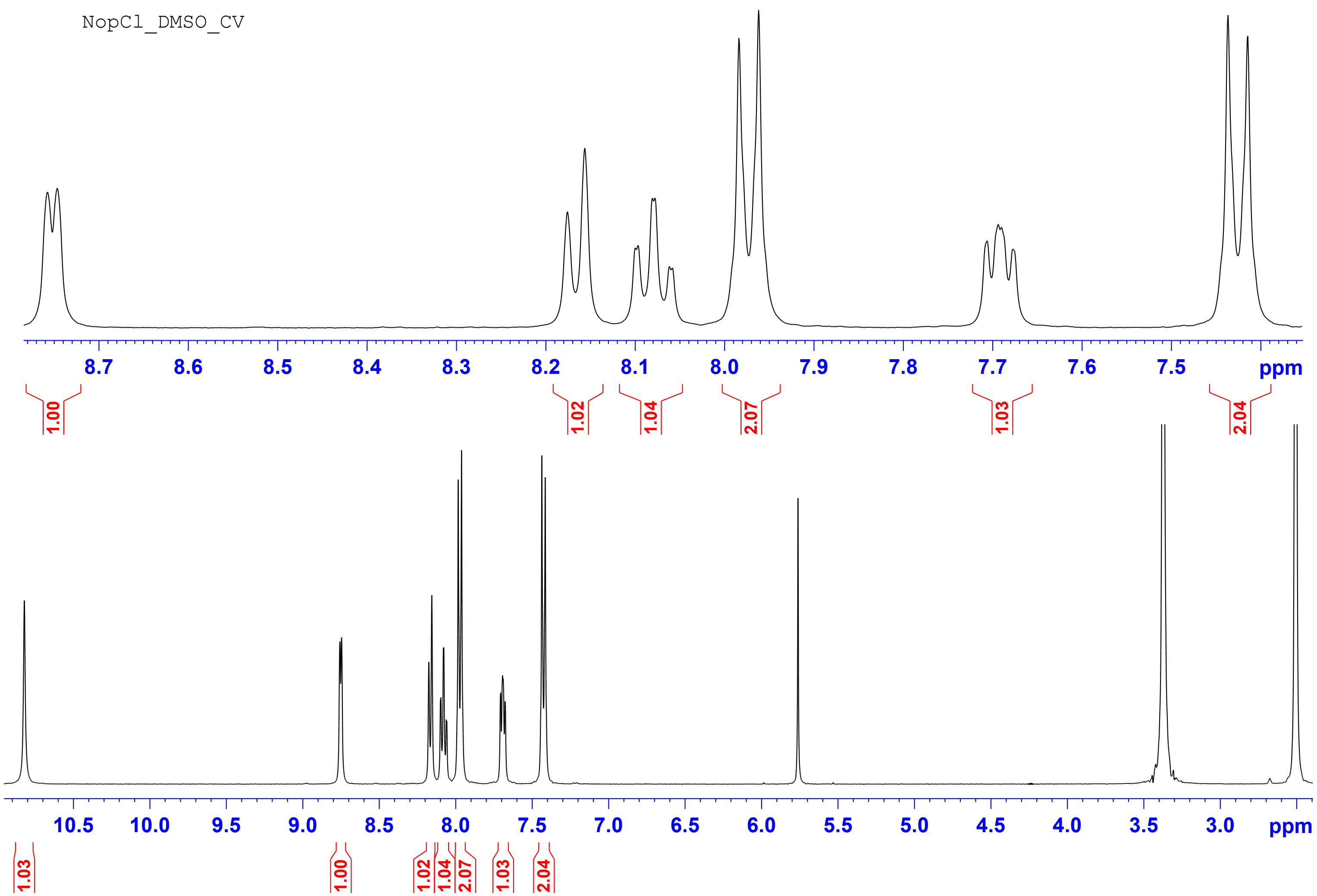

Spectrum 27: ^{1}H-NMR of **NopCl** in DMSO-d_6

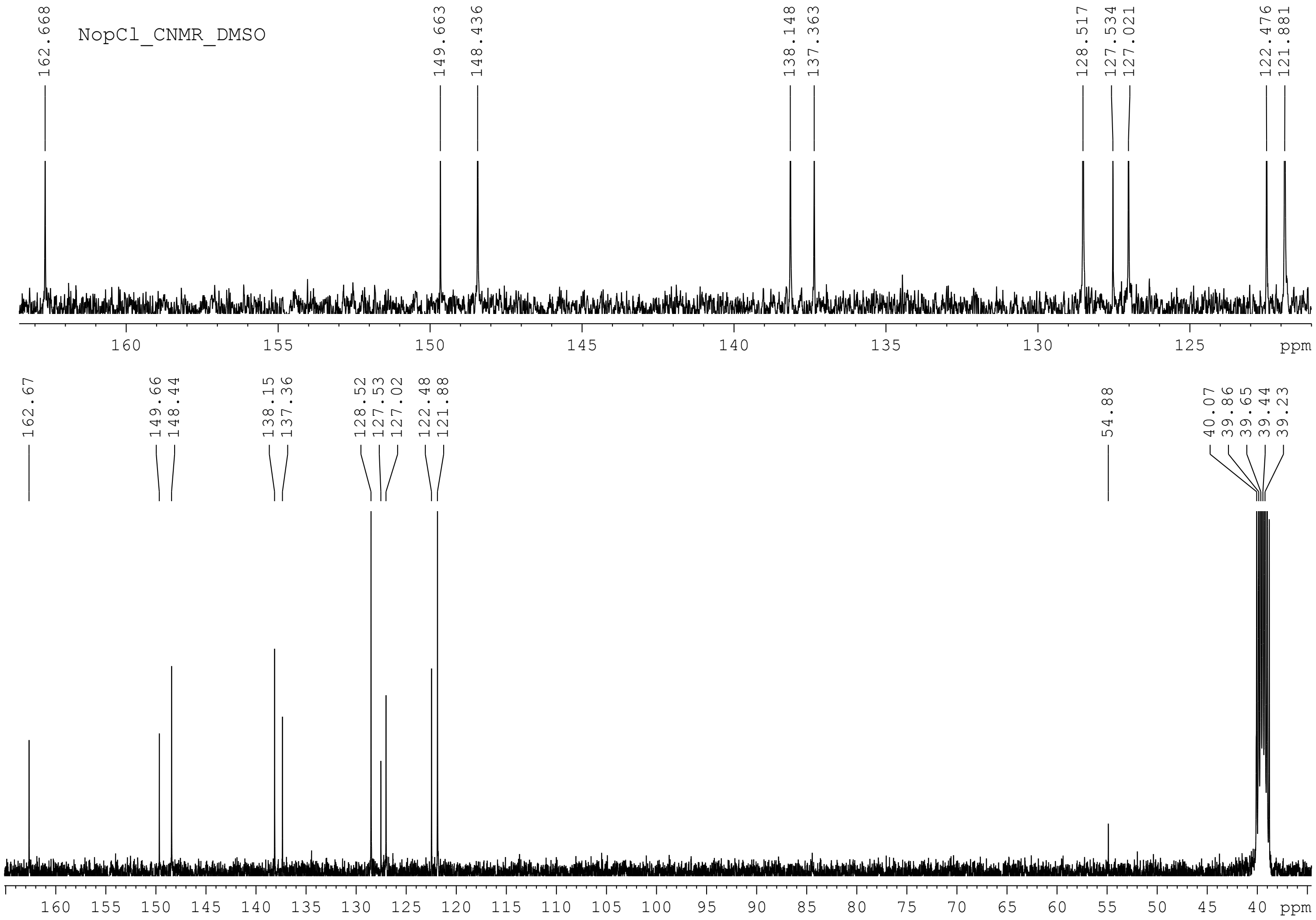

Spectrum 28: ^{13}C-NMR of **NopCl** in DMSO-d_6

NomCl data

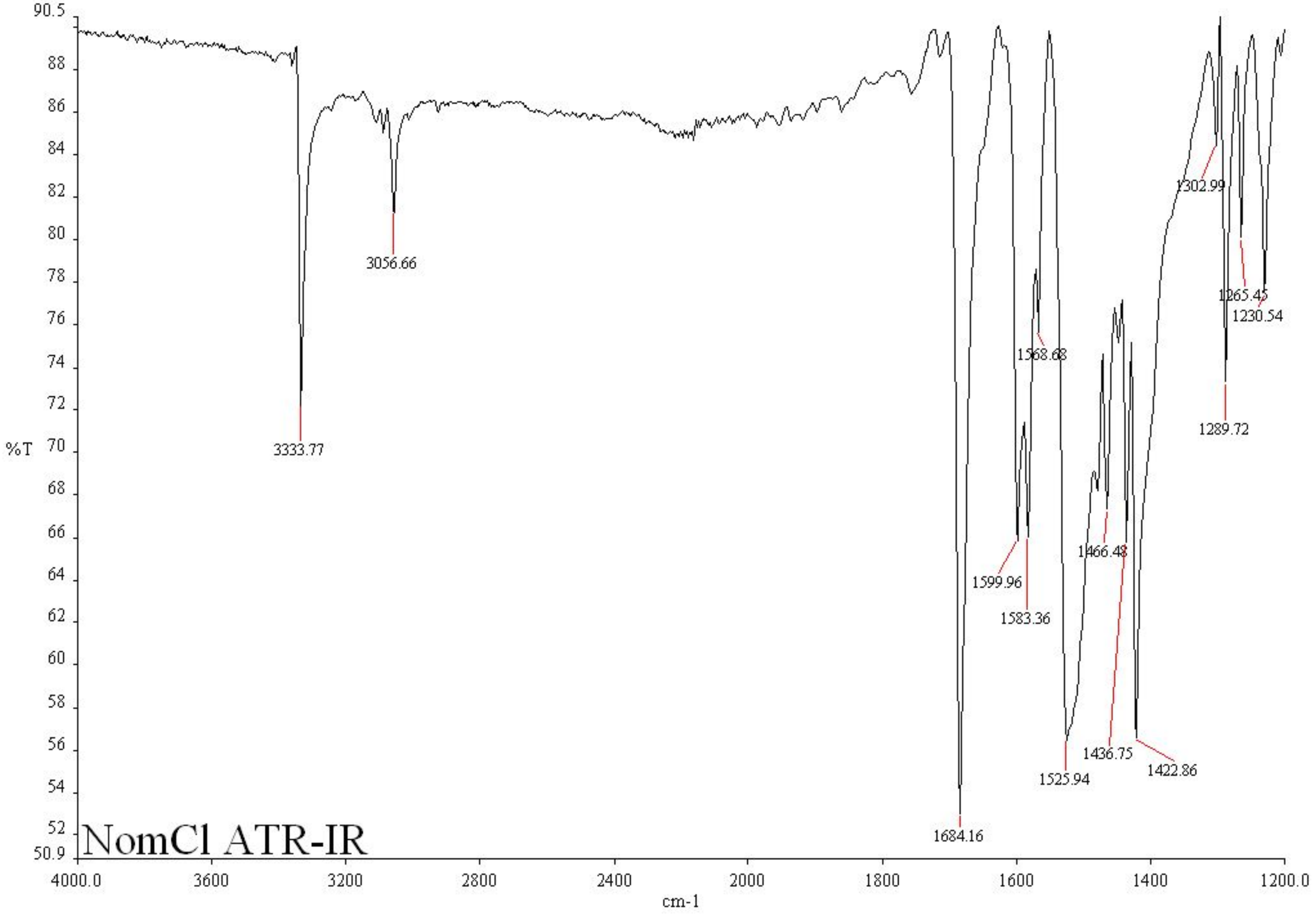

Spectrum 29: ATR-IR spectrum of **NomCl**

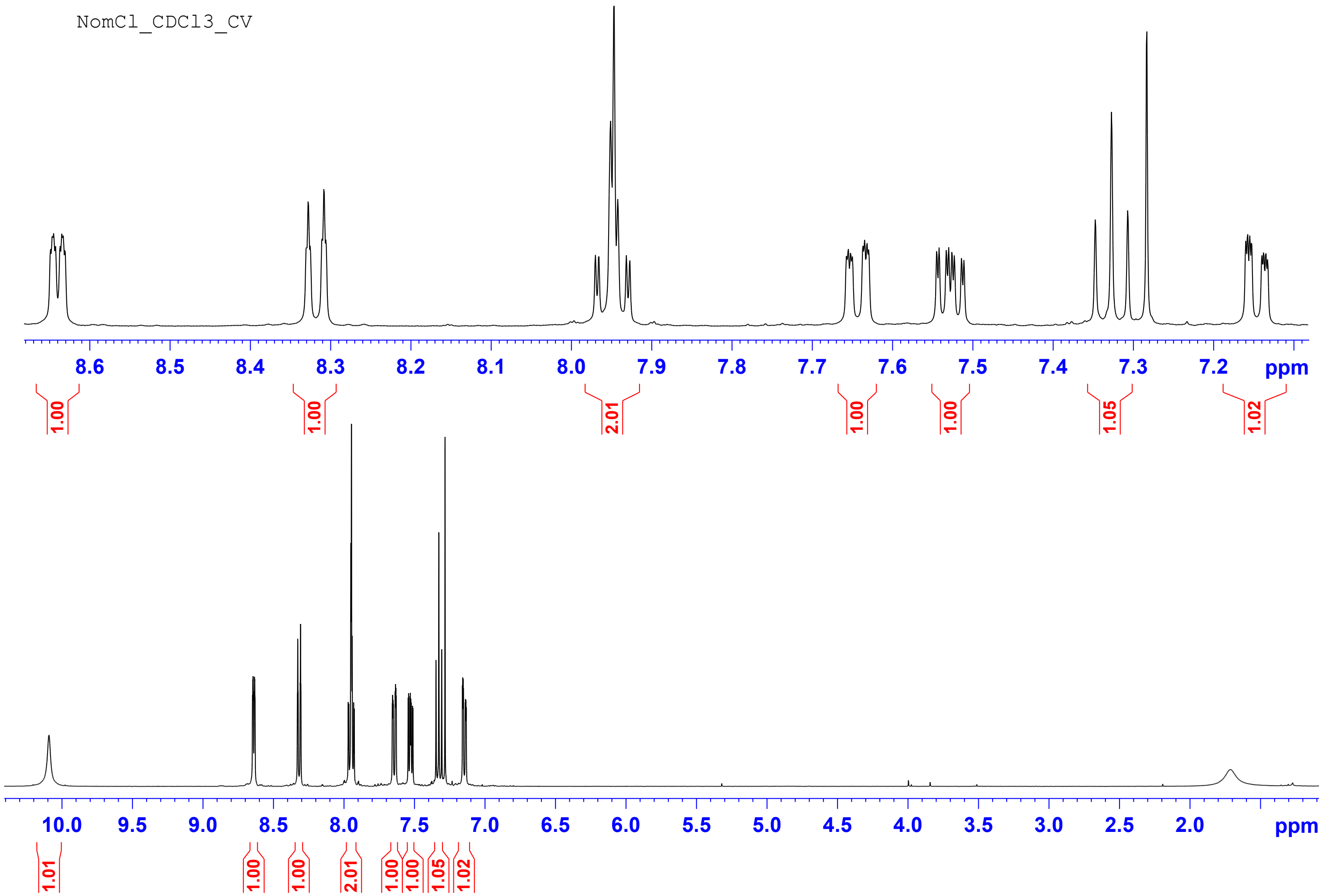

Spectrum 30: ^{1}H-NMR of **NomCl** in $CDCl_3$

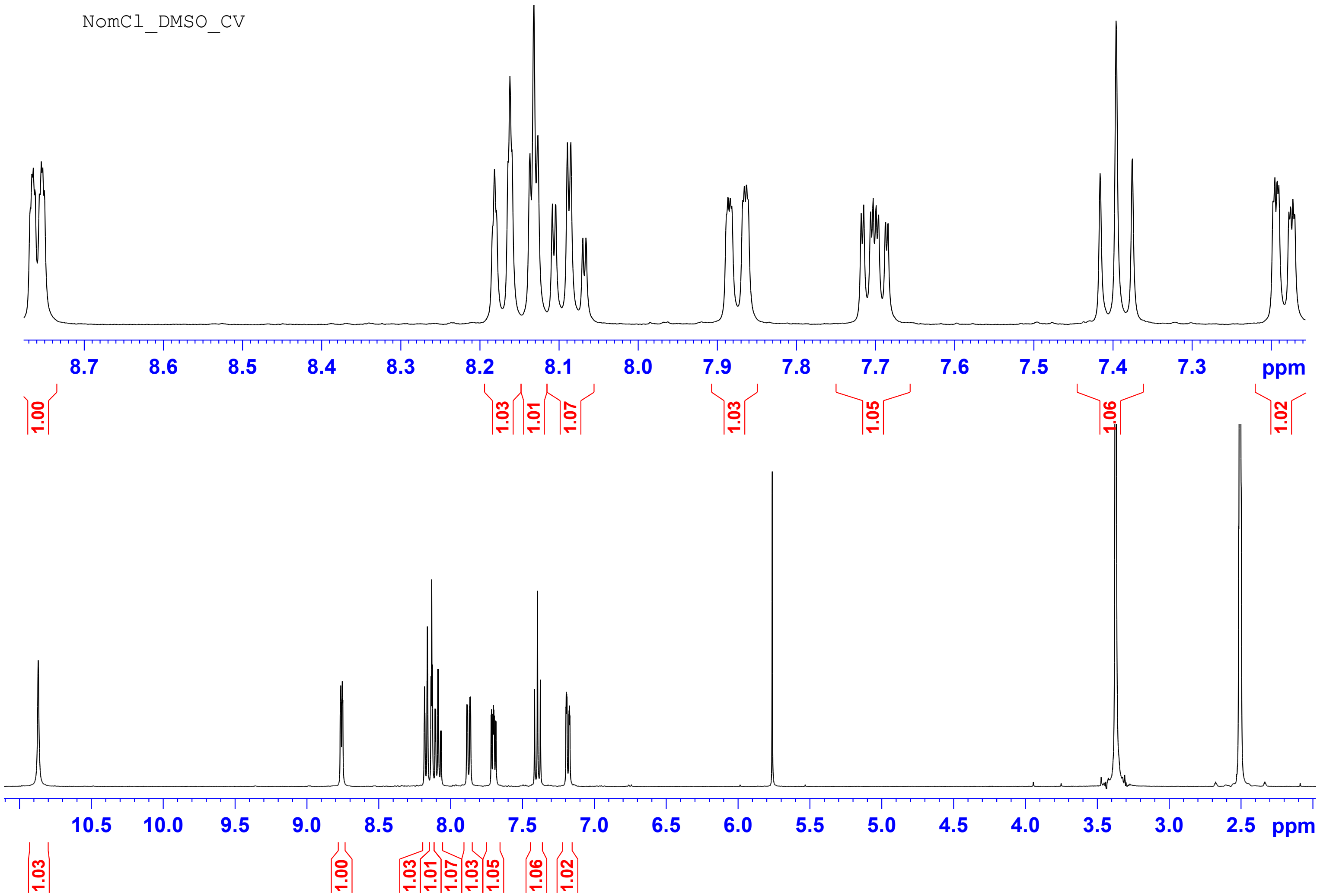

Spectrum 31: ^{1}H-NMR of **NomCl** in DMSO-d_6

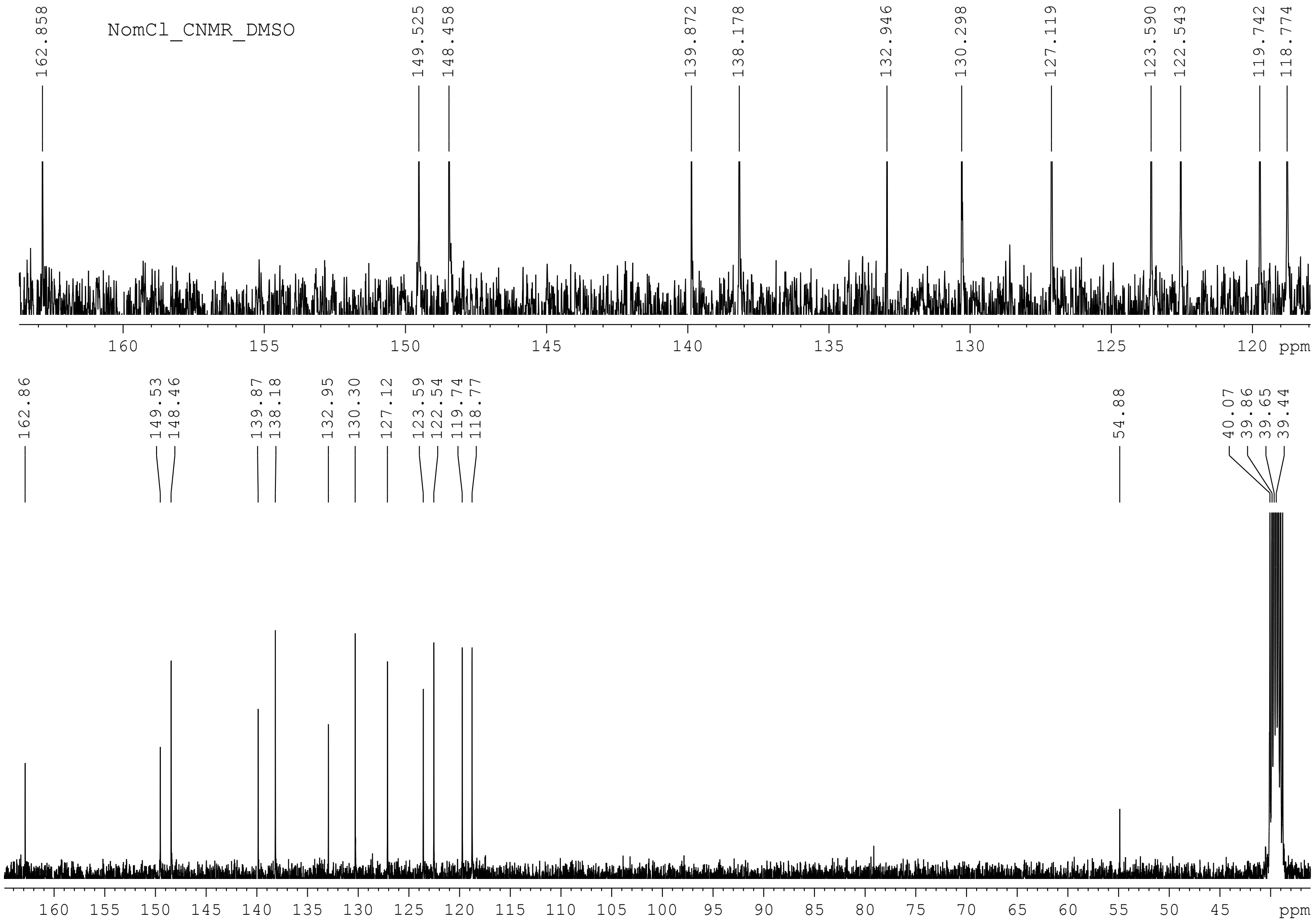

Spectrum 32: ^{13}C-NMR of **NomCl** in DMSO-d_6

NooCl data

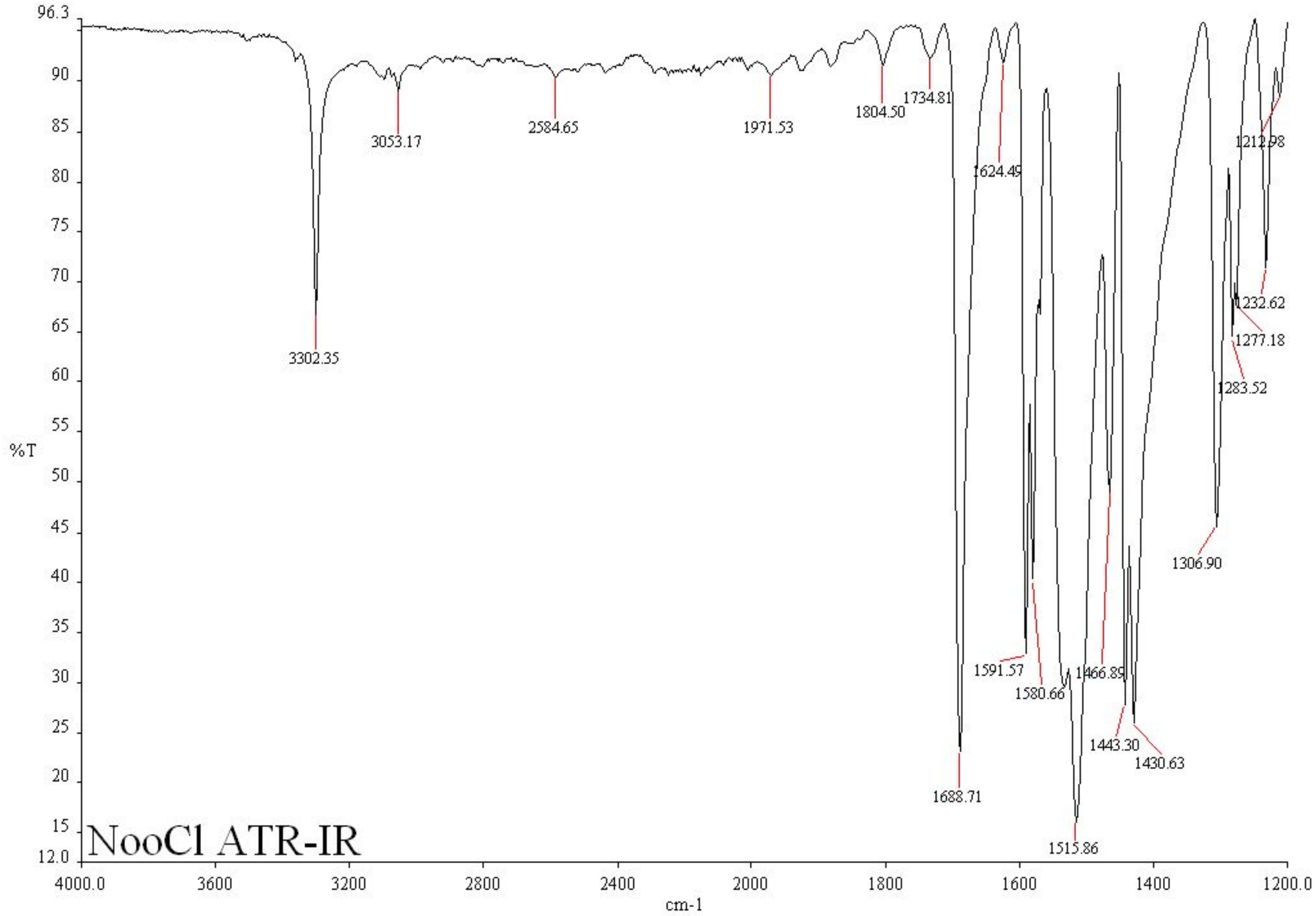

Spectrum 33: ATR-IR spectrum of **NooCl**

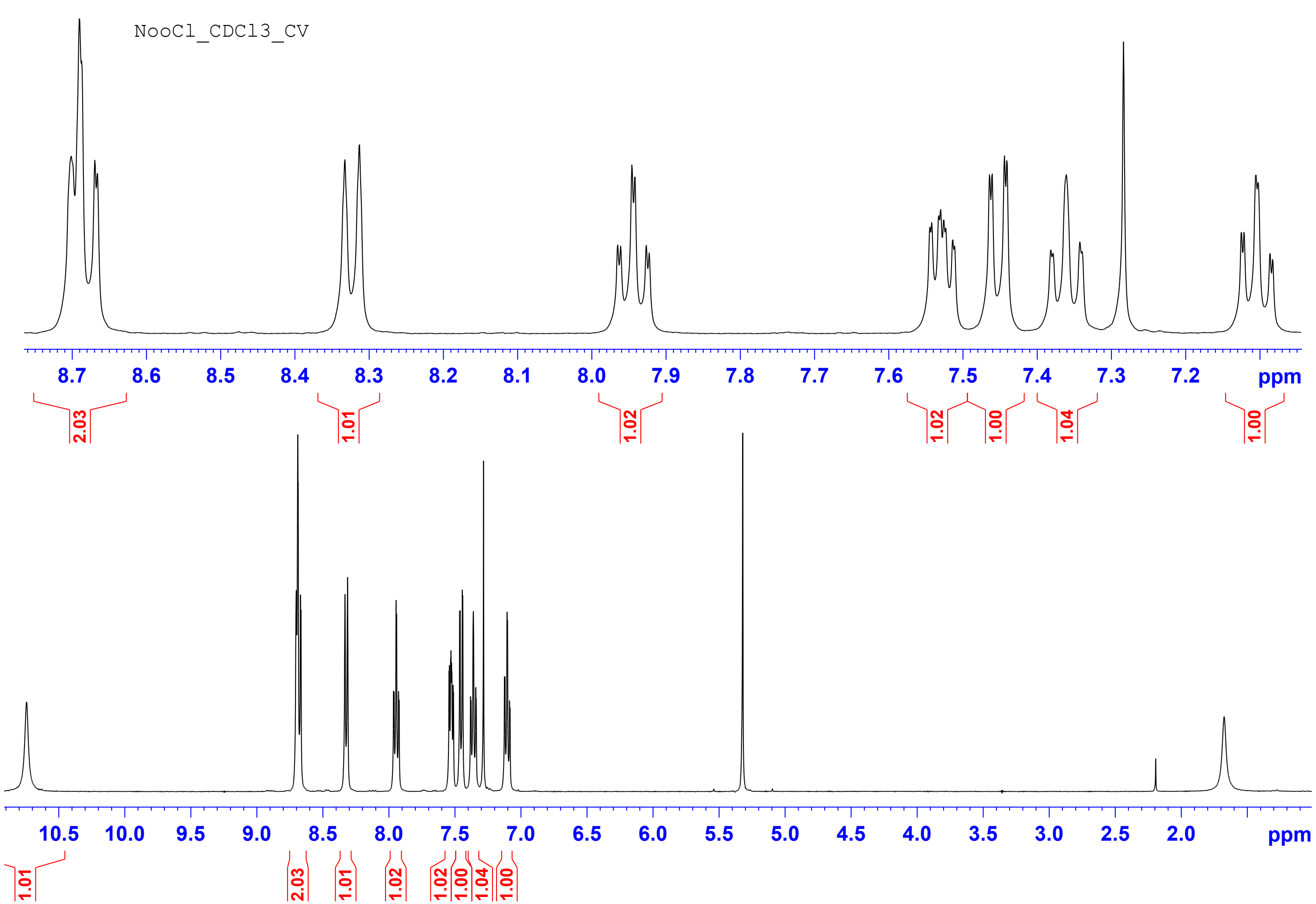

Spectrum 34: ^{1}H-NMR of **NooCl** in $CDCl_3$

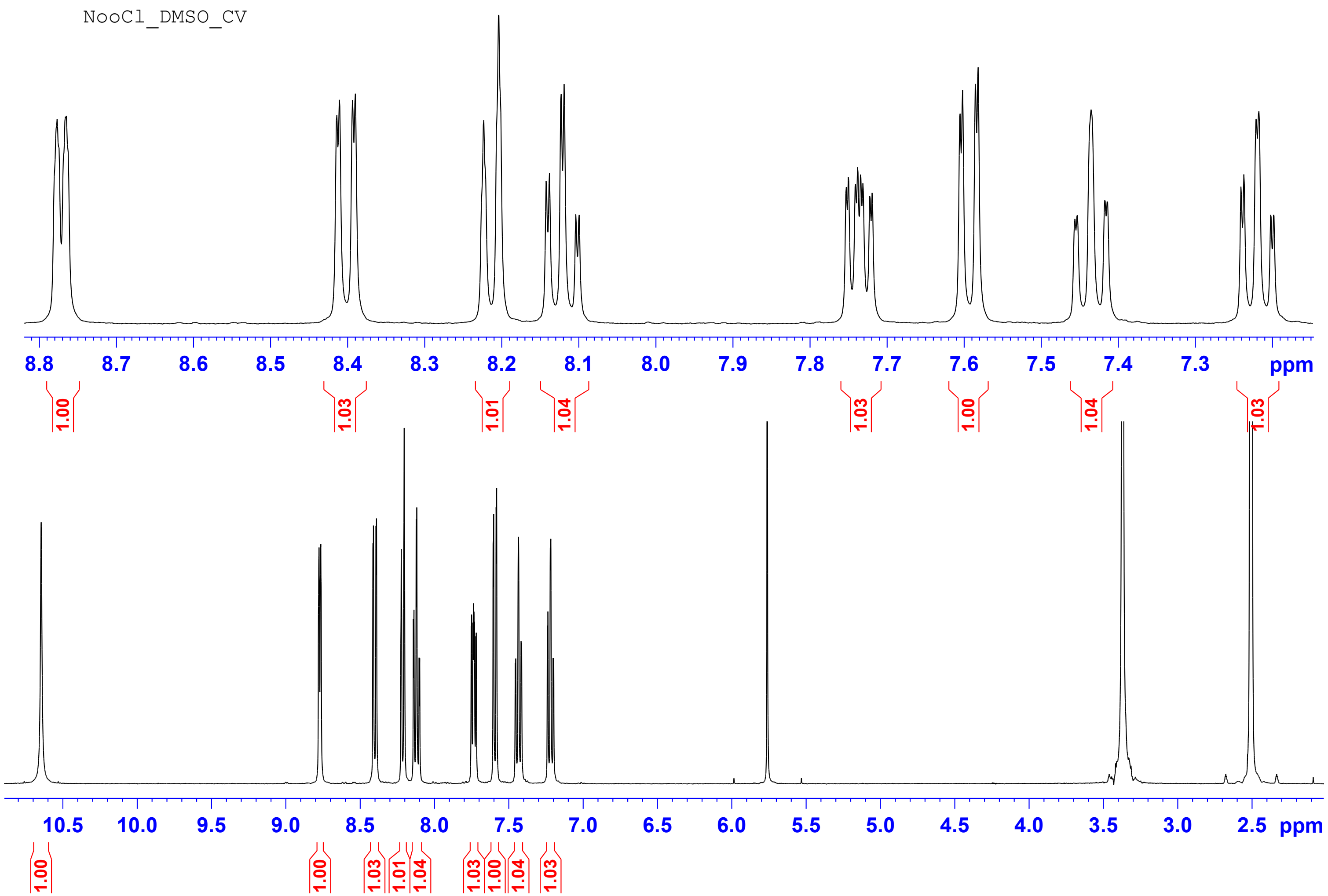

Spectrum 35: ^{1}H-NMR of **NooCl** in DMSO-d_6

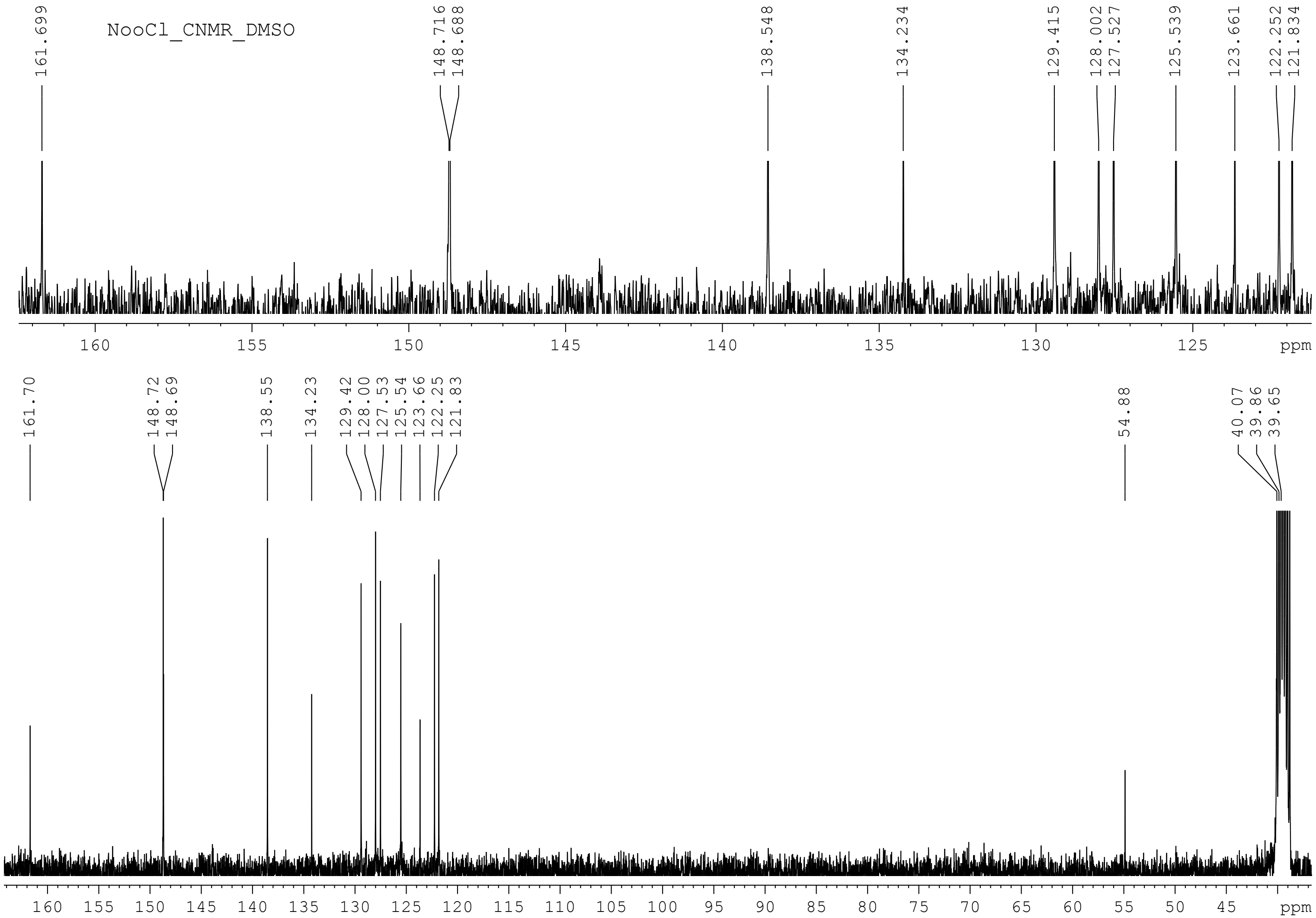

Spectrum 36: ^{13}C-NMR of **NooCl** in DMSO-d_6

ATR-IR Diagram (a composite of the nine NxxCl spectra)

3 x 3 isomer grid of ATR-IR spectra

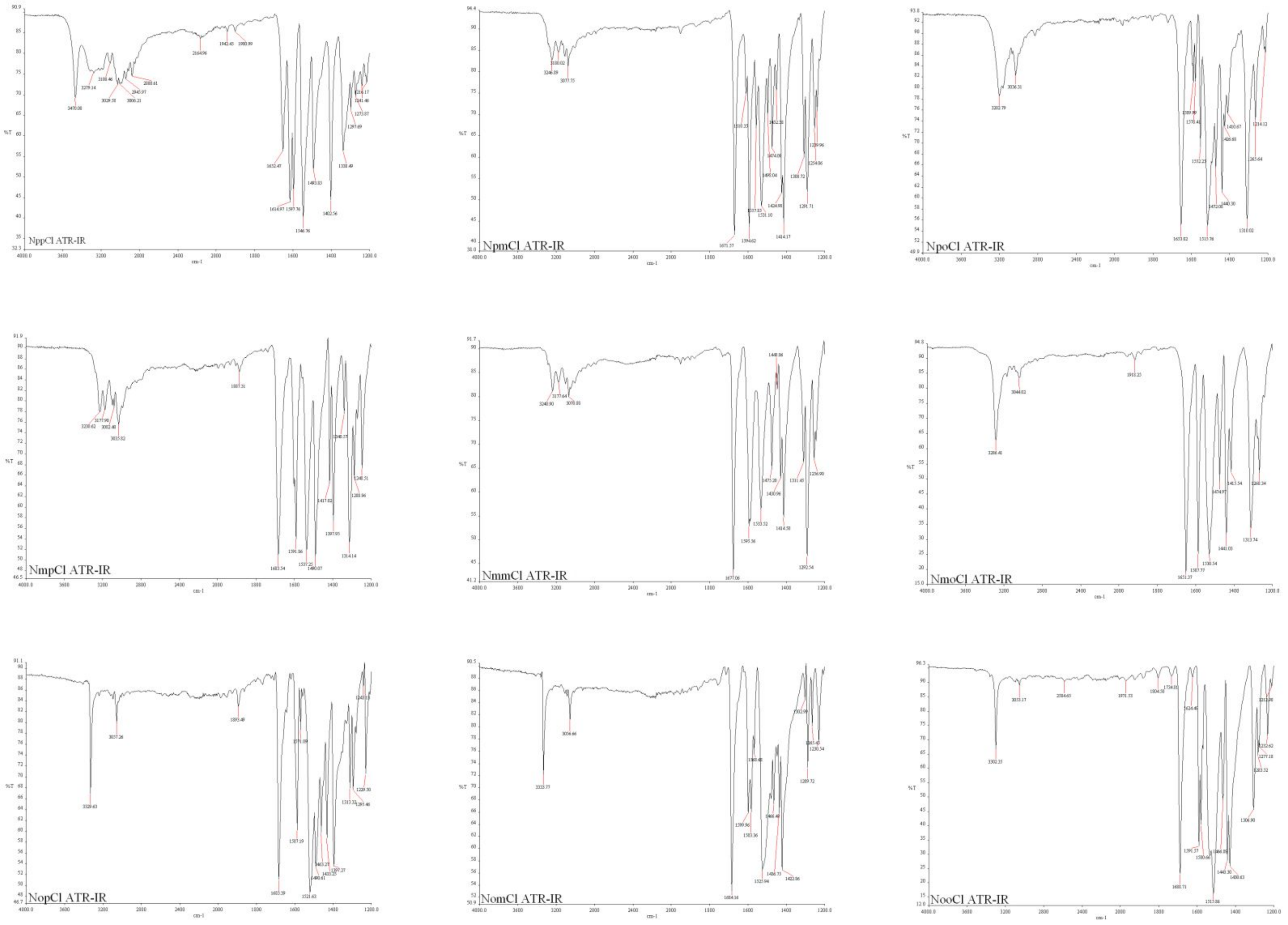

Section IV (Supplementary electrostatic energy diagrams).

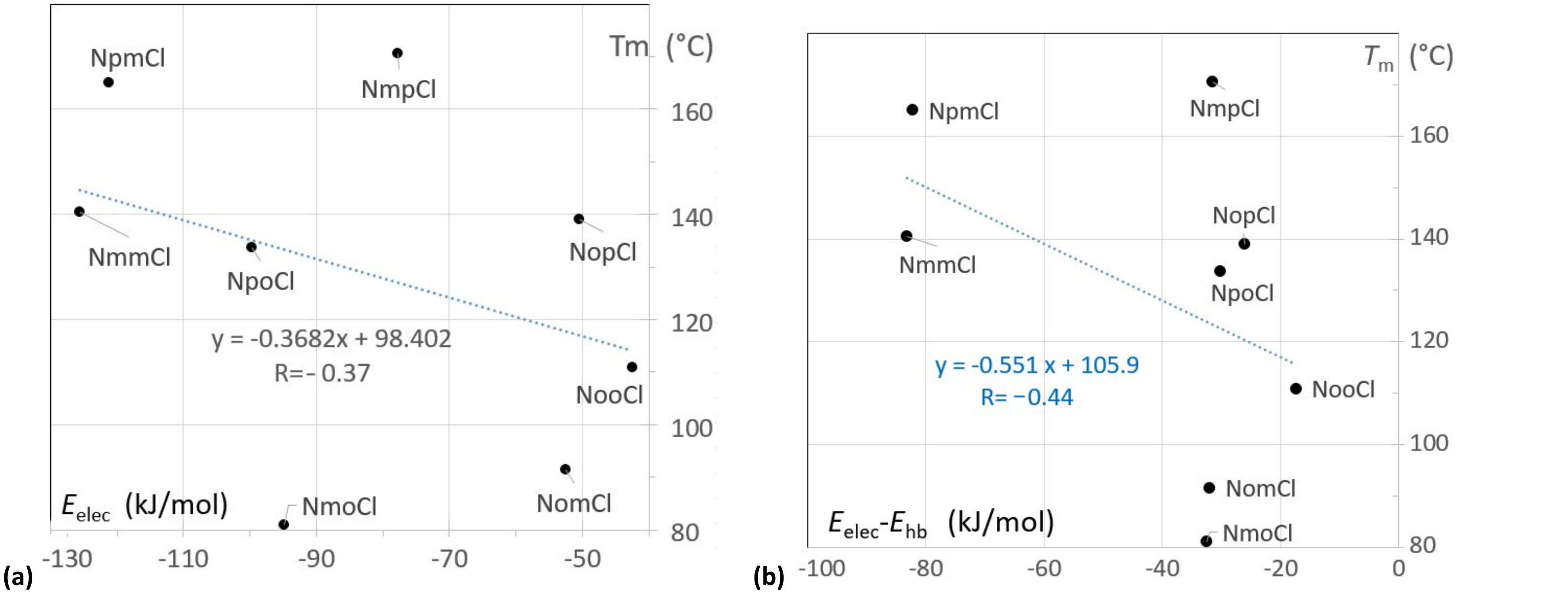

Figure S01: Scatterplot of melting points T_m and **(a)** the electrostatic lattice energies E_{elec}, **(b)** the differences E_{elec}–E_{HB}

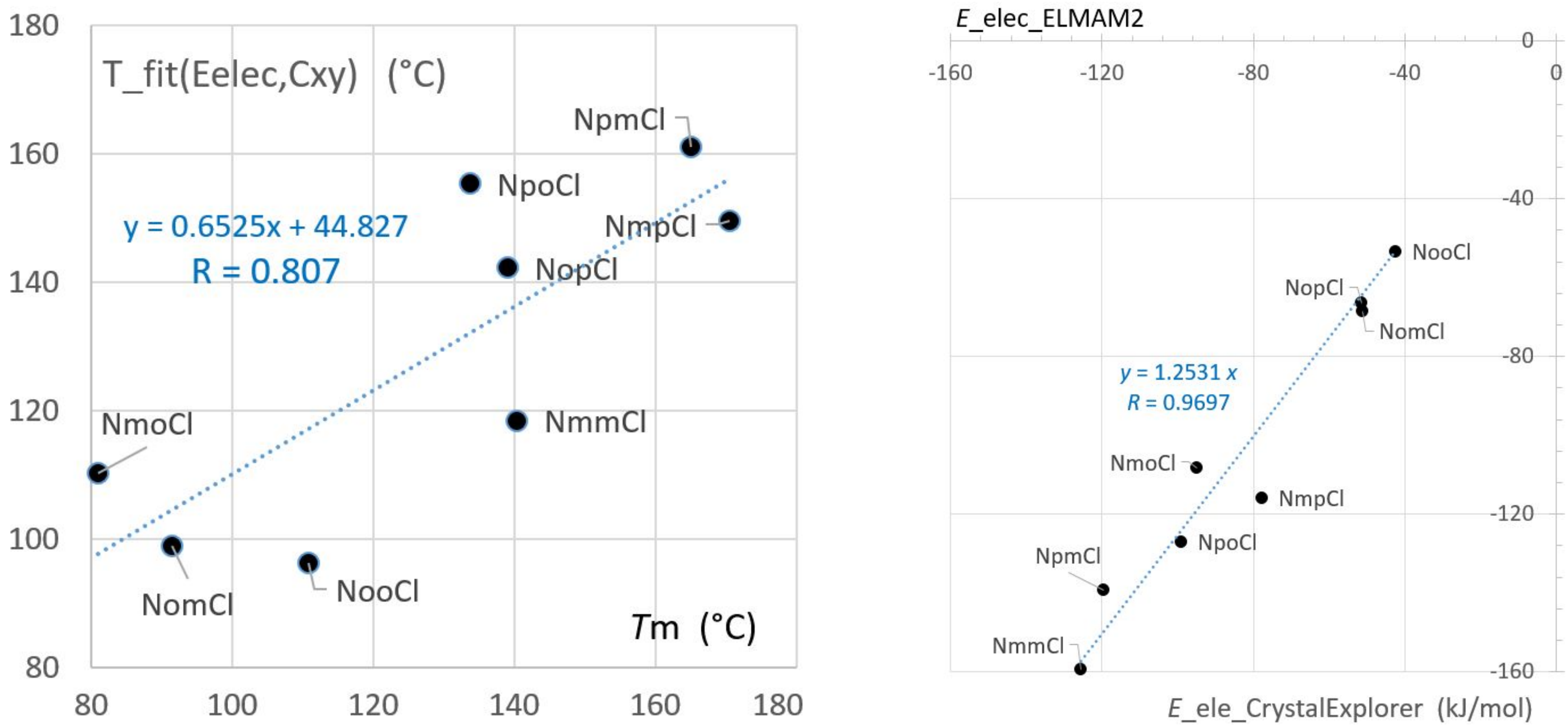

Figure S02: (left) Double linear regression of melting point T_m on the Carnelley molecule symmetry descriptor C_{XY} and the E_{elec}, the electrostatic lattice energy.

Figure S03: (right) Scatterplot of lattice electrostatic energy obtained from the multipolar atom model (ELMAM2 databank)[68] and from CrystalExplorer (non-scaled values).[71]

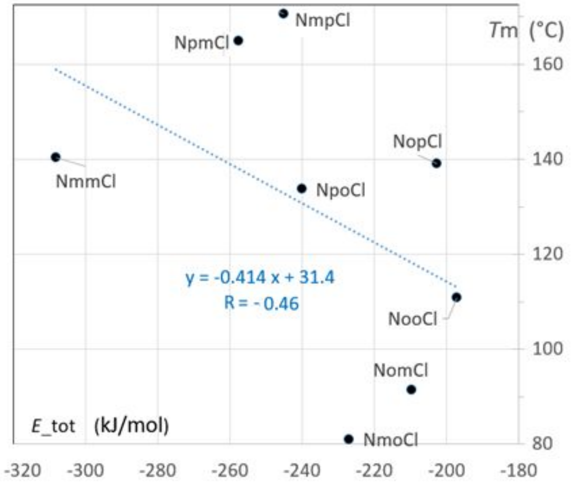

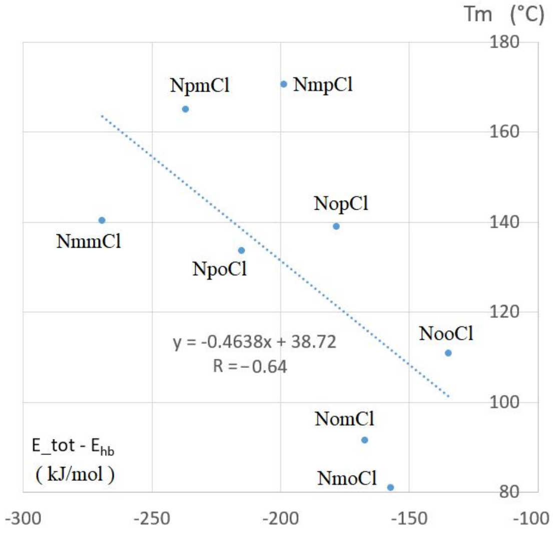

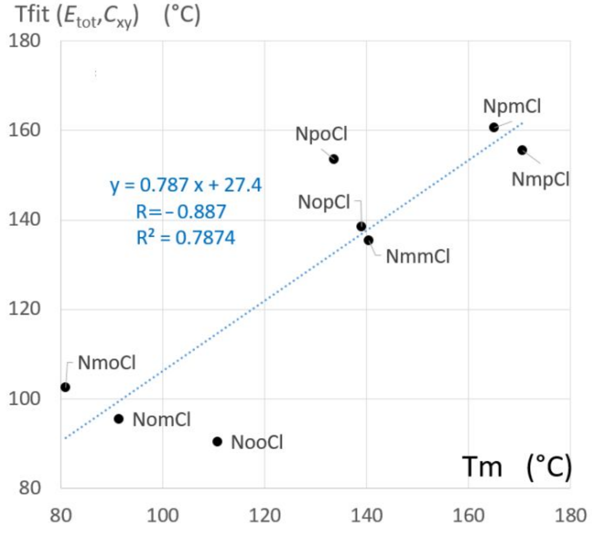

Figure S04: (left) Scatterplot of lattice total energy as calculated from CrystalExplorer (scaled summation)[71] and T_m melting points.

Figure S05: (middle) Scatterplot of total energy E_{tot} - E_{HB} strongest hydrogen bond electrostatic energy and T_m melting points.

Figure S06: (right) Double linear regression of T_m on E_{tot} and C_{XY} variables.

Section V

Contact enrichment studies for NxxCl

Table S5: The contact enrichment ratios for the eight anhydrous **NxxCl** crystal structures. Outlier numbers highlighted in **bold** are discussed.

Contact	NmmCl	NmoCl	NmpCl	NomCl	NooCl	NopCl	NpmCl	NpoCl
C...C	**0.69**	0.9	**1.71**	**1.75**	1.26	1.39	1.11	**0.45**
Hc...C	**1.42**	1.14	0.68	0.48	0.86	0.63	1.12	**1.56**
Hc...Hc	0.22	0.77	0.93	0.96	0.94	0.99	0.54	0.41
Cl...C	1.03	1.29	0.46	0.61	0.57	0.79	0.86	1.4
Hc...Cl	1.66	1.27	1.76	2.04	1.33	1.63	1.48	1.27
Cl...Cl	0	0.04	**1.26**	0	**1.66**	0.71	0.73	0
N...C	0.35	0.78	0.83	1.53	1.54	1.69	0.76	0.17
O...C	0.82	0.65	0.78	0.36	0.66	0.84	0.08	0.33
C...Hn	0.72	0.01	0.13	0.87	2.02	1.29	0.94	0.21
Cl...N	0.03	0	0	0	0.6	0	0.16	0.09
Cl...O	0.44	1.09	0.03	0	0	0	0.74	0.54
Hc...Hn	0.53	0.36	0.6	1.32	0.65	1.26	0.42	0.29
Cl...Hn	0	0	0	0	0.28	0	0	0
Hc...N	1.43	1.88	0.79	1.14	0.73	1	1.14	1.77
Hc...O	1.72	0.41	1.94	1.8	1.91	1.68	2.33	0.99
O...N	0.23	0	0	0.08	0	0.43	0	0.33
N...N	0.05	0	0.74	0.46	2.02	0.17	0.53	2.95
Hn...Hn	0	0	0.1	0	0	0	0	0
N...Hn	10.54	0	12.72	0	0	0	9.17	1.94
O...Hn	0	17.28	0	3.76	0	1.43	0	13.27
O...O	0	0	0.05	0.89	0	0	0	0

Table S6: Enrichment of contacts with chlorine in the **NxxCl**$^{\text{this work}}$ and with fluorine in the **NxxF** isomer series.[50]

Element	C	H_C	Cl/F	N	H_N	O
**Cl**	0.9(3)	1.6(3)	0.6(7)	0.1(2)	0.0(1)	0.4(4)
**F**	1.0(4)	1.3(3)	0.8(14)	0.4(4)	0.1(4)	0.1(3)
